# Inhibition of inflammatory signaling in *Pax5* mutant cells mitigates B-cell leukemogenesis

**DOI:** 10.1038/s41598-020-76206-y

**Published:** 2020-11-05

**Authors:** Marta Isidro-Hernández, Andrea Mayado, Ana Casado-García, Jorge Martínez-Cano, Chiara Palmi, Grazia Fazio, Alberto Orfao, Jordi Ribera, Josep Maria Ribera, Lurdes Zamora, Javier Raboso-Gallego, Oscar Blanco, Diego Alonso-López, Javier De Las Rivas, Rafael Jiménez, Francisco Javier García Criado, María Begoña García Cenador, Manuel Ramírez-Orellana, Giovanni Cazzaniga, César Cobaleda, Carolina Vicente-Dueñas, Isidro Sánchez-García

**Affiliations:** 1 Experimental Therapeutics and Translational Oncology Program, Instituto de Biología Molecular y Celular del Cáncer, CSIC-USAL, Campus M. de Unamuno s/n, Salamanca, Spain; 2 Institute for Biomedical Research of Salamanca (IBSAL), Salamanca, Spain; 3 Servicio de Citometría, Departamento de Medicina, Biomedical Research Networking Centre on Cancer CIBER- CIBERONC (CB16/12/00400), Institute of Health Carlos III, and Instituto de Biología Molecular y Celular del Cáncer, CSIC/Universidad de Salamanca, Salamanca, Spain; 4 Immune system development and function Unit, Centro de Biología Molecular Severo Ochoa (Consejo Superior de Investigaciones Científicas -Universidad Autónoma de Madrid), Madrid, Spain; 5 Centro Ricerca Tettamanti, Dept of Medicine, University of Milan Bicocca, Monza, Italy; 6 Josep Carreras Leukaemia Research Institute (IJC), Badalona, Spain; 7 Catalan Institute of Oncology-Germans Trias i Pujol, Badalona, Spain; 8 Departamento de Anatomía Patológica, Universidad de Salamanca, Salamanca, Spain; 9 Bioinformatics Unit, Cancer Research Center (CSIC-USAL), Salamanca, Spain; 10 Bioinformatics and Functional Genomics Research Group, Cancer Research Center (CSIC-USAL), Salamanca, Spain; 11 Departamento de Fisiología y Farmacología, Universidad de Salamanca, Edificio Departamental, Campus M. de Unamuno s/n, 37007 Salamanca, Spain; 12 Departamento de Cirugía, Universidad de Salamanca, Salamanca, Spain; 13 Department of Pediatric Hematology and Oncology, Hospital Infantil Universitario Niño Jesús, Universidad Autónoma de Madrid, Madrid, Spain

**Keywords:** Cancer, Cancer genetics, Cancer models, Cancer therapy

## Abstract

*PAX5* is one of the most frequently mutated genes in B-cell acute lymphoblastic leukemia (B-ALL), and children with inherited preleukemic *PAX5* mutations are at a higher risk of developing the disease. Abnormal profiles of inflammatory markers have been detected in neonatal blood spot samples of children who later developed B-ALL. However, how inflammatory signals contribute to B-ALL development is unclear. Here, we demonstrate that *Pax5* heterozygosis, in the presence of infections, results in the enhanced production of the inflammatory cytokine interleukin-6 (IL-6), which appears to act in an autocrine fashion to promote leukemia growth. Furthermore, in vivo genetic downregulation of *IL-6* in these *Pax5* heterozygous mice retards B-cell leukemogenesis, and in vivo pharmacologic inhibition of IL-6 with a neutralizing antibody in *Pax5* mutant mice with B-ALL clears leukemic cells. Additionally, this novel IL–6 signaling paradigm identified in mice was also substantiated in humans. Altogether, our studies establish aberrant IL6 expression caused by *Pax5* loss as a hallmark of Pax5-dependent B-ALL and the IL6 as a therapeutic vulnerability for B-ALL characterized by *PAX5* loss.

## Introduction

Hematopoietic development is a tightly regulated process requiring precise control, both at the cell-intrinsic (transcriptional and epigenetic) and extrinsic (cytokines and other permissive or inductive signals) levels, and any alteration of these controls leads to unfavorable outcomes. B-cell acute lymphoblastic leukemias (B-ALLs) are clonal malignancies caused by the loss of appropriate control over the proliferation and/or differentiation along B cell development^1^. Genetic alterations in regulators of B-lymphoid development are present in approximately two-thirds of cases of B-ALL^2^. PAX5 is arguably one of the most important transcription factors required for correct B cell development^3^, and it can be involved in B-ALL at different stages of the disease, being altered in more than one-third of B-ALL cases by deletion, sequence mutations, or translocation with different fusion partners^3–6^. Besides these somatically acquired *PAX5* mutations, it has been shown that inherited hypomorphic variants of the gene predispose the carriers to the development of familiar B-ALL^7,8^. Studies both in affected human carriers and in animals carrying an heterozygous null mutation of *Pax5* (*Pax5*^+*/-*^ mice, henceforth also called “*Pax5* mutant mice”) have shown that *Pax5* mutant mice tend to accumulate an expanded, aberrant, vulnerable, population of B cell progenitors prone to malignant transformation through the accumulation of secondary mutations in the presence of a selective pressure^7–9^. These accumulated cells are commonly defined as preleukemic cells^10^, and the selection and expansion of these preleukemic-B clones precede the development of B-ALL, both in the case of carriers of congenital *PAX5* mutations, and also in almost all other B-ALL-predisposing somatically arising mutations studied to date^10–12^. Preleukemic cells remain latent until they progress to the development of full-blown B-ALL through acquisition of additional somatic mutations over time^1^. We have recently shown that natural exposure to infectious pathogens contribute to the ‘‘switch’’ from a preleukemic state to a leukemic state in cells bearing these *PAX5* mutations^9,13^, and *Pax5* mutant mice go on to develop B-ALL with modest penetration when they are exposed to natural infections^9^, usually associated with the inactivation of the other copy of *Pax5*. In this context, inflammation has been hypothesized to play an essential role^14–16^, but precisely how inflammatory signals influence the *Pax5* mutant B-cell leukemogenesis process is poorly understood. Human populations with different ancestries have been naturally selected to present many allelic differences in the genes involved in immune system development and immune response^17^. All these variants allow specific responses (stronger or weaker) against certain types of infections^17^, but can also increase the chances of developing certain diseases; in the case of B-ALL, this can be caused by an imbalance in the immune system that helps disease progression. Given the limited genetic variation present in experimental mice, the results from these models suggest that the contribution of the rest of the individual genetic variations beyond the preleukemia-initiating one does not seem to play a major role in the transition from the preleukemic phase to B-ALL, a fact further supported by the possibility of triggering B-ALL conversion ex vivo with TLR ligands^18–20^. Considering that *Pax5*-mutant preleukemic cells give rise to infection-triggered B-ALL in both human and mice^7–9^, the relationship between inflammation and B-cell leukemogenesis is likely to be B-cell-dependent. Because preleukemic precursor B cells reside in the bone marrow, an inflammatory microenvironment can influence the growth of these cells in part by producing pro-inflammatory cytokines. In the present study, we asked whether the inflammatory signals contribute to B-ALL development triggered by environmental infection exposure as a result of *Pax5*-inherited susceptibility. The results from our experiments identify IL-6 as a key cytokine whose expression and secretion by mouse leukemic B-cells is induced when *Pax5* is lost during the course of B-ALL development, and whose inhibition therapeutically targets leukemic cells.

## RESULTS

### ***Pax5***^+***/-***^ mice developing B-ALL show enhanced expression of the proinflammatory cytokine IL-6

**Figure 1:**
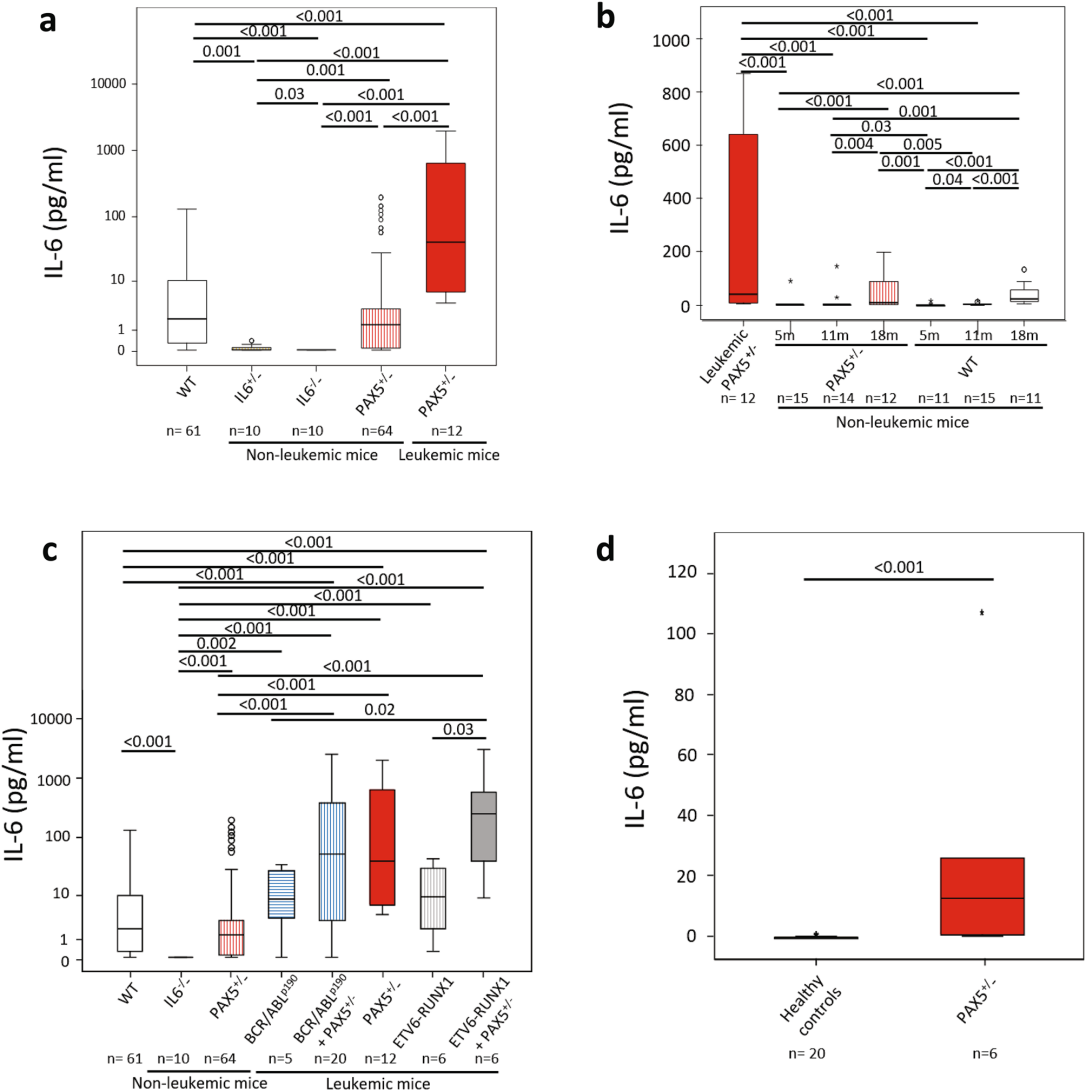
IL-6 serum levels in mice and humans with B-ALL. (**a**) IL-6 serum levels in IL-6^+*/-*^, IL-6^*-/-*^ and *Pax5*^+*/-*^ non-leukemic mice and *Pax5*^+*/-*^ mice that develop B-ALL vs control wild-type mice. All mice were exposed to an infectious environment as described in the Methods section. (**b**) IL-6 serum concentrations in *Pax5*^+*/-*^ and control wild type mice at different ages vs leukemic *Pax5*^+*/-*^ mice. (**c**) IL-6 levels in serum from *Sca1-BCR/ABL*^*p190*^ + *Pax5*^+*/*+^, *Sca1-BCR/ABL*^*p190*^ + *Pax5*^+*/-*^, *Sca1-ETV6-RUNX1* and *Sca1-ETV6-RUNX1* + *Pax5*^+*/-*^ mice. (**d**) Serum levels of IL-6 in human B-ALL patients who carry *PAX5* alterations vs healthy donors. Notched-boxes extend from the 25th to the 75th percentile values; the lines in the middle and vertical lines correspond to median values and the 10th and 90th percentiles, respectively. The Kruskal–Wallis test was used to interpret differences.

To determine whether the transformation of preleukemic B cells to full-blown B-ALL is in part due to dysregulated expression of inflammatory cytokines in *Pax5*^+*/-*^ mice, we measured concentrations of 7 inflammatory cytokines (IL-2, IL-4, IL-6, IL-10, IL-17a, TNF and IFNγ) in the serum of *Pax5*^+*/-*^ mice exposed to an infectious environment which developed B-ALL, exposed *Pax5*^+*/-*^ mice without B-ALL, and age-matched control wild-type mice. We found that leukemic *Pax5*^+*/-*^ mice had abnormal concentrations of IL-6 (Fig. 1a and Supplementary Fig. 1). The emergence of this increase in IL-6 levels could further be linked to disease onset, since IL-6 in serum samples taken at routine intervals confirmed lack of IL-6 increase in the exposed *Pax5*^+*/-*^ mice that never developed B-ALL (Fig. 1a-b, Supplementary Table S1 and Supplementary Fig. 1). However, IL-6 increase was not detectable in serum samples taken at routine intervals in healthy (non-leukemic) *Pax5*^+*/-*^ mice that later developed B-ALL, prior to the first phenotypic signs of illness (Fig. 1b and Supplementary Fig. 2). In addition, this increase in IL-6 was not observed in mouse models where the appearance of B-ALL is triggered by infection exposure but is not linked to a congenital *Pax5* alteration, like in *Sca1-BCR-ABLp190* and *Sca1-ETV6-RUNX1* mice where the second hit does not involve *Pax5* inactivation (Fig. 1c)^21^. However, elevated levels of IL-6 protein could be measured at the time of B-ALL diagnosis in the serum of *Pax5*^+*/-*^;*Sca1-ETV6-RUNX1* leukemic mice and *Pax5*^+*/-*^*;Sca1-BCR-ABLp190* leukemic mice^22^ (Fig. 1, Supplementary Table S1). These elevated levels of IL-6 protein could be recapitulated at the time of diagnosis in the serum of human B-ALL carrying *PAX5* alterations (and lacking known JAK/STAT mutations) compared to Healthy Donors (HD) (Fig. 1d, Supplementary Table S2). Thus, induction of a leukemogenic state by *Pax5*-loss is associated with an increase in IL-6 secretion in both human patients and mouse models, suggesting the IL-6 secretion may correspondingly be the product of oncogenic *Pax5* inactivation.

### *Pax5* controls IL-6 expression in B-cells

**Figure 2:**
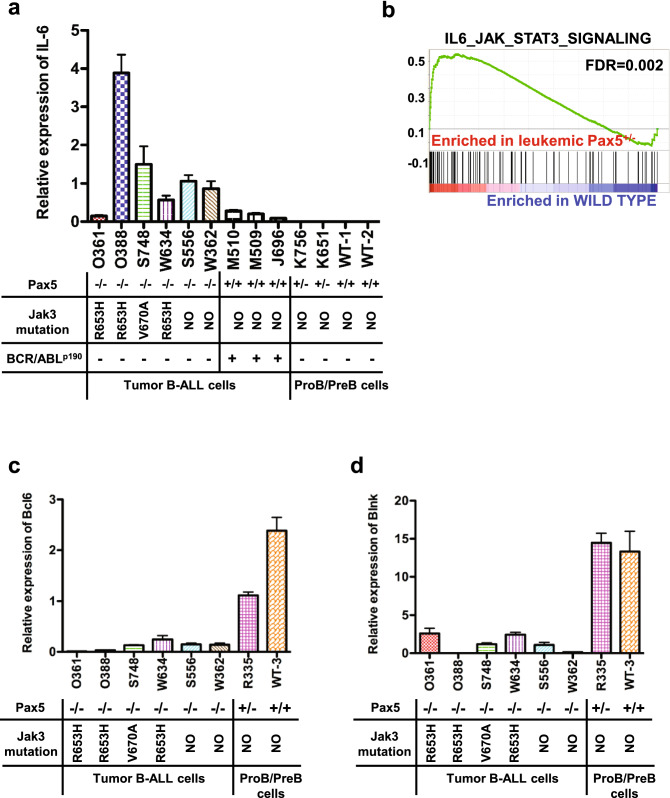
*Pax5* controls IL-6 expression in B-cells. (**a**) Relative expression of *mIL-6* in leukemic *Pax5*^+*/-*^ and *Pax5*^+*/*+^ (*BCR-ABL*^*p190*^ +) cells obtained from different individual mice (mouse codes shown on the X axis) compared to proB cells from healthy *Pax5*^+*/-*^ and WT proB cells. The total bone marrow of a WT mouse was used as a reference. Error bars represent the mean + /- the standard deviation of 3 replicates. (**b**) GSEA showing that leukemic *Pax5*^+*/-*^ cells are enriched in the IL-6_JAK_STAT3 signaling geneset (FDR = 0.002). (**c**) Relative expression of *mBcl6* in leukemic *Pax5*^*-/-*^ proB cells and compared with healthy *Pax5*^+*/-*^ and WT proB cells. The total bone marrow of a WT mouse was used as a reference. Error bars represent the mean + /- the standard deviation of 3 replicates. (**d**) Relative expression of *mBlnk* in leukemic *Pax5*^*-/-*^ proB cells and compared with healthy *Pax5*^+*/-*^ and WT proB cells. The total bone marrow of a WT mouse was used as a reference. Error bars represent the mean + /- the standard deviation of 3 replicates.

We investigated the mechanisms controlling IL-6 production using leukemic proB cells and both control wild-type and *Pax5*^+*/-*^ proB-cells. Leukemic proB cells, lacking *Pax5* activity due to secondary mutations of the WT *Pax5* allele^9^, displayed high levels of IL-6 mRNA cells (Fig. 2a), which was not significant in *Pax5*^+*/*+^ leukemic cells and it was not detectable neither in control wild-type proB-cells nor in control *Pax5*^+*/-*^ proB-cells (Fig. 2a). However, high levels of IL-6 mRNA are present in healthy *Pax5*^*-/-*^ precursor B cells (Supplementary Fig. 3). A microarray analysis of gene expression confirmed enrichment of the IL-6 signaling pathway geneset only in leukemic proB cells (Fig. 2b and Supplementary Fig. 4A-B) as well as an enrichment in inflammatory response and apoptosis gene sets (Supplementary Fig. 4C-D). We then investigated leukemic proB cells lacking *Pax5* activity for expression of a panel of effectors genes known to regulate IL-6 expression, and we found a significant downregulation of both *Blnk* (which is a Pax5 direct target^23^) and *Bcl6* expression (Fig. 2c-d). BCL6 is a direct transcriptional repressor of the *IL-6* gene^24^ and recent work has shown that STAT5 activation inhibits BCL6 expression^25^. Therefore, *Pax5*-deficient leukemic proB cells had decreased *Bcl6* levels and this, together with the STAT5 activation, could contribute to the upregulation of IL-6 expression observed in *Pax5*-deficient leukemic proB cells. These results indicate that *Pax5* activity controls IL-6 expression in proB cells, and suggest that both Bcl6 and STAT5 activation are involved in the complex molecular network mediating this effect. Taken together, these data suggest that IL-6 might be important for *Pax5*-deficient B-ALL development. Thus, we next examined whether IL-6 plays any role in *Pax5*-mediated B-cell leukemogenesis.

### Impairment of IL-6 signaling in ***Pax5***^+***/-***^ mice delays natural infection-driven B-ALL development

**Figure 3:**
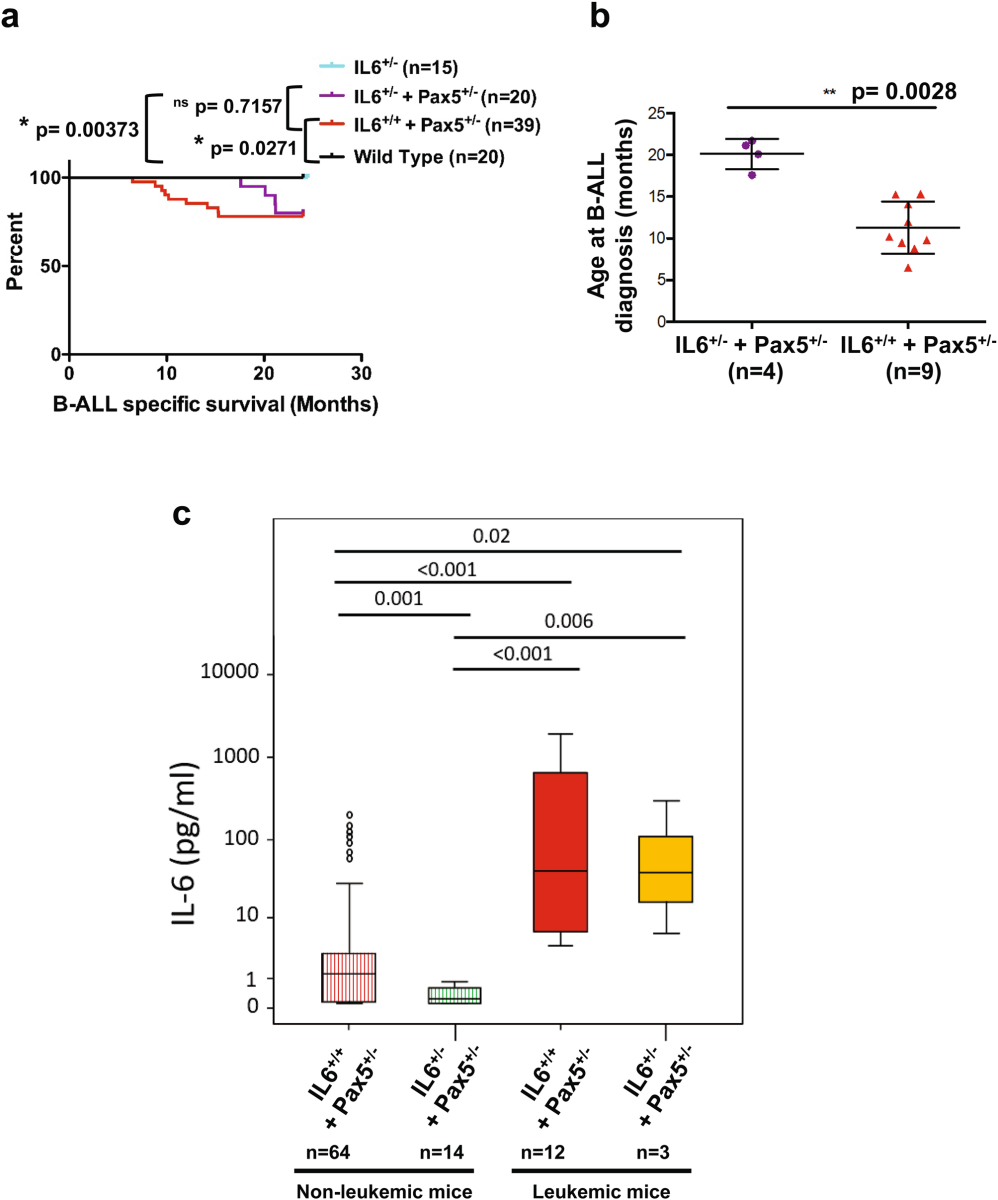
Impairment of IL-6 signaling in *Pax5*^+*/-*^ mice delays natural infection-driven B-ALL development. (**a**) B-ALL-specific survival of *IL-6*^+*/-*^ (light blue line, n = 15), *IL6*^+/+^/*Pax5*^+*/-*^ (red line, n = 39), *IL-6*^+*/-*^/*Pax5*^+*/-*^ (purple line, n = 20) and wild-type mice (black line, n = 20), all of them exposed to common infections. Log-rank (Mantel-Cox) test p-value = 0.00373 when comparing *IL-6*^+*/-*^/*Pax5*^+*/-*^ vs WT and p-value = 0.0271 when comparing *IL6*^+/+^/*Pax5*^+*/-*^ vs WT and p-value = 0.7157 when comparing *IL-6*^+*/-*^*/Pax5*^+*/-*^ vs *IL-6*^+*/*+^*/Pax5*^+*/-*^. (**b**) The median age of *IL-6*^+*/-*^*/Pax5*^+*/-*^ and *IL6*^+/+^/*Pax5*^+*/-*^ mice at B-ALL diagnosis. Error bars represent the mean and SD. For the significant differences, unpaired t-test p-values are indicated. **c**) IL-6 serum levels in non-leukemic mice (*IL-6*^+*/+*^ /*Pax5*^+*/-*^, *IL-6*^*+/-*^/*Pax5*^+*/-*^) and leukemic mice (*IL6*^+/+^/*Pax5*^+*/-*^, *IL-6*^+*/-*^/*Pax5*^+*/-*^) vs control wild-type mice. Notched-boxes extend from the 25th to the 75th percentile values; the lines in the middle and vertical lines correspond to median values and the 10th and 90th percentiles, respectively. The Kruskal–Wallis test was used to interpret differences.

**Figure 4:**
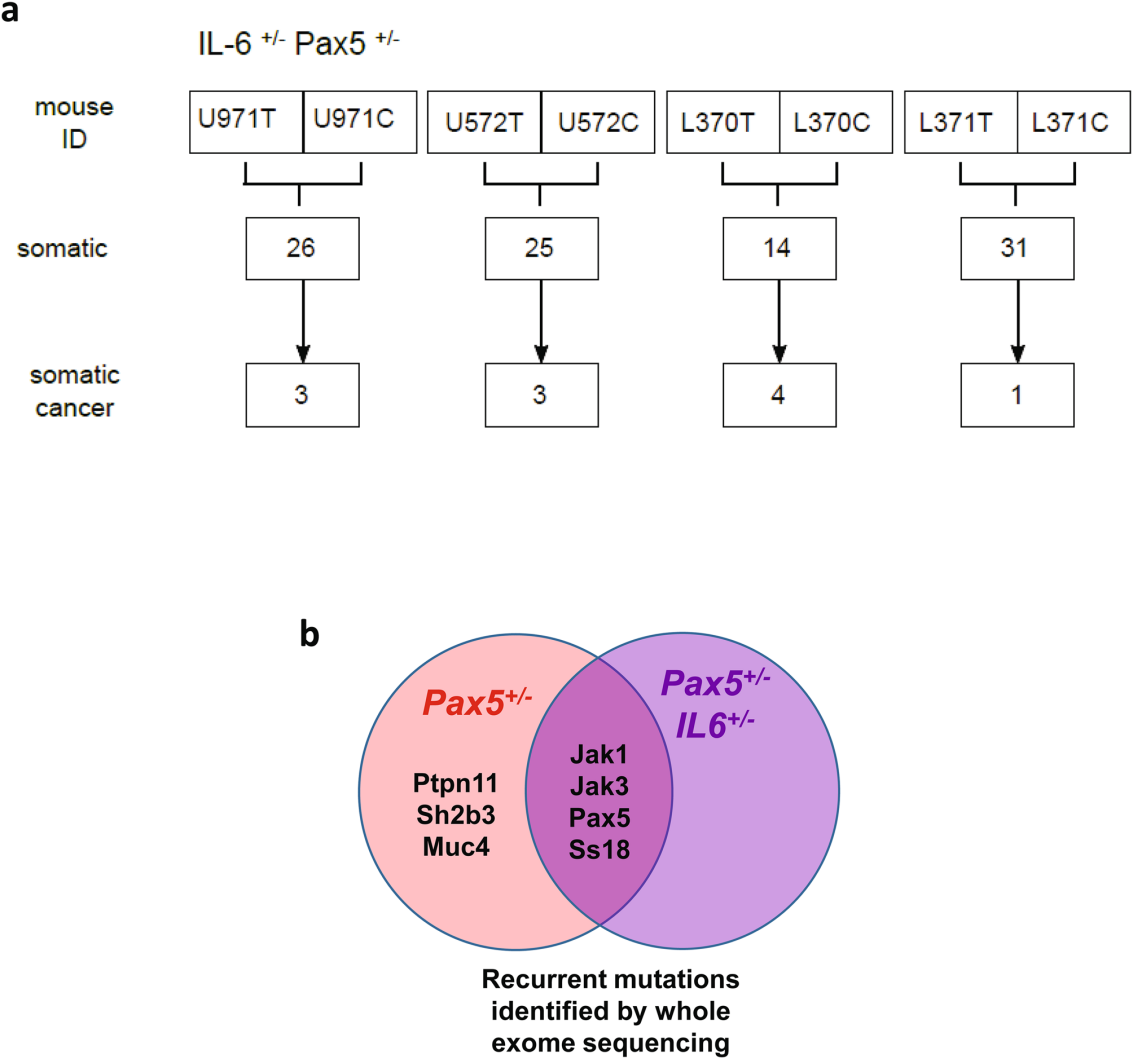
Mouse tumor exome sequencing in *IL-6*^+*/-*^/*Pax5*^+*/-*^ B-ALL. (**a**) Whole-exome sequencing analysis of tumor and control samples. Tumor-specific somatic mutations were determined by *mutect* and *varscan* analysis. The number of somatic cancer genes was calculated by using the cancer gene consensus list. The percentage of leukemic cells for each mouse was: 98% (U971) from total BM, 90% (U572) from total BM, 90% (L370) from total BM and 20% (L371) from total LN. (**b**) Genomic comparison between mutations driving native B-ALL as a result of natural infection exposure of *IL-6*^+*/*+^/*Pax5*^+*/-*^ (previously described in Martin-Lorenzo, A. et al*.*^9^) (orange) and *IL-6*^+*/-*^/*Pax5*^+*/-*^ mice (violet), respectively, showed that similar second hits were affected by recurrent mutations.

Given the observed upregulation of IL-6 in *Pax5*-deficient leukemic proB cells and mice, we next directly tested the requirement for IL-6 in *Pax5*-loss mediated leukemia growth in a system that recapitulates the spontaneous process of leukemogenesis. To functionally demonstrate the role of IL-6, we impaired the expression of IL-6 by breeding *Pax5*^+*/-*^ mice to *IL-6*^+/−^ mice, which have significantly lower IL-6 serum concentrations (Fig. 1a), with the aim of testing whether *IL-6*^+*/-*^/*Pax5*^+*/-*^ mice are more resistant to infection-induced B-ALL than *Pax5*^+*/-*^ mice. Accordingly, control *IL-6*^+/+^/*Pax5*^+*/-*^ and experimental *IL-6*^+*/-*^/*Pax5*^+*/-*^ mice were exposed to natural infections, and B-ALL development was monitored as previously described^9^. The results showed that the reduction on IL-6 levels significantly delayed the emergency of leukemias in *Pax5*^+*/-*^ mice upon exposure to natural infections (Fig. 3). B-ALL appeared between 6 and 16 months of age in *IL-6*^+/+^/*Pax5*^+*/-*^ mice (mean = 11.29 months) and, at the end of the 24 months’ experimental period, 22% of the mice had developed B-ALL (Fig. 3a-b), in line with previously reported results. In sharp contrast, B-ALL was significantly delayed in the *IL-6*^+*/-*^/*Pax5*^+*/-*^ mice, appearing between 18 and 22 months of age (mean = 20.13 months) (Fig. 3a-b), although by the termination of the experiment a similar percentage (20%) of the mice had developed B-ALL and IL-6 was elevated in their serum similarly to *IL-6*^+/+^/*Pax5*^+*/-*^ leukemias (Fig. 3c). These *IL-6*^+*/-*^/*Pax5*^+*/-*^ B-ALLs are histologically, phenotypically and genetically similar to *IL-6*^+/+^/*Pax5*^+*/-*^ B-ALLs, and FACS analyses revealed a CD19^+*/-*^B220^+^IgM^-^cKit^+*/-*^CD25^+*/-*^ cell surface phenotype for tumor cells that extended through bone marrow (BM), peripheral blood (PB), spleen and lymph nodes (Supplementary Fig. 5) and infiltrated non-lymphoid tissues like liver and intestine (Supplementary Fig. 6). All *IL-6*^+*/-*^/*Pax5*^+*/-*^ B-ALLs displayed clonal immature BCR rearrangements (Supplementary Fig. 7). We then characterized the global expression signature of *IL-6*^+*/-*^/*Pax5*^+*/-*^ B-ALLs and compared it with the expression signature of both healthy WT pro-B/pre-B cells and *IL-6*^+/+^/*Pax5*^+*/-*^ leukemias. The analysis showed a similar differential gene expression profile (FDR = 0.05) between expression patterns in *IL-6*^+*/-*^*/ Pax5*^+*/-*^ B-ALL, and *Pax5*^+*/-*^ B-ALL with just 196 probe-sets differentially expressed (Supplementary Fig. 8A and Supplementary Table S3) in contrast to the huge differences in terms of gene expression between *IL-6*^+*/-*^/*Pax5*^+*/-*^ B-ALL and healthy WT proB cells with 9160 probe-sets differentially expressed (Supplementary Fig. 8B and Supplementary Table S4). We next aimed to confirm that the specific significant delay in B-ALL development due to *IL-6*^+*/-*^ heterozygosity does not modify the B-cell susceptibility and genetic characteristics of B-ALL in *Pax5*^+*/-*^ mice; similar to *IL-6*^+/+^/*Pax5*^+*/-*^ mice, preleukemic *IL-6*^+*/-*^/*Pax5*^+*/-*^ littermates presented a significantly reduced amount of total B-cells in the PB when compared to *Pax5*^+*/*+^ (WT) littermates of the same breeding (Supplementary Fig. 9), but this PB B-cell decrease was similar to the one observed in *IL-6*^+/+^/*Pax5*^+*/-*^ mice (Supplementary Fig. 9). In order to further identify somatically acquired 2nd hits leading to leukemia development, we next performed whole exome sequencing of 4 *IL-6*^+*/-*^/*Pax5*^+*/-*^ B-ALLs and corresponding germline on a HiSeq 2500 (Illumina) platform. *IL-6*^+*/-*^/*Pax5*^+*/-*^ tumor DNA was derived from whole leukemic BM or lymph nodes, while tail DNA of the respective mouse was used as reference germline material. Similar to *IL-6*^+/+^/*Pax5*^+*/-*^ leukemias^9^, *IL-6*^+*/-*^/*Pax5*^+*/-*^ tumors showed recurrent mutations affecting *Pax5, Ss18, Jak1,* and *Jak3* (Fig. 4). Taken together, these data demonstrate that the decrease of IL-6 delays spontaneous formation of infection-driven B-ALL in *Pax5*^+*/-*^ mice. Collectively, our data suggest that knocking down amplified IL6 levels represents a formidable barrier to *Pax5*-dependent leukemogenesis, a finding of clear clinical relevance.

### *Pax5* mutant B-ALL is not sensitive to IL-6 inhibition in transplant-based mouse models

To test the hypothesis that IL-6 inhibition may represent a preferential target for *Pax5*-dependent B-ALLs, and to expand our studies to more clinically relevant settings, we first tested whether IL-6 contributes to Pax5-dependent B-ALL maintenance, since IL-6 is a secreted protein, and as such it is amenable to targeting by neutralizing antibodies^26^ and, moreover, such an antibody treatment has already been shown to be tolerated in humans^27^. In the past, the efficiency of experimental therapies against B-ALL has been routinely measured by transplanting leukemic B cells into recipient mice that had been preconditioned by irradiation^28,29^. However, a potential limitation of this approach to studies focused on how inflammatory signaling affects B-ALL, resides in the fact that irradiation triggers the production of pro-inflammatory cytokines such as IL-1, IL-6 and TNF^30,31^. Nevertheless, to be consistent with previous studies in the literature, we first tested the efficacy of the neutralizing IL-6 antibody in mice infused with leukemic *Pax5*^+*/−*^ proB cells harboring the *Jak3*^V670^^A^ mutation^9^ (Supplementary Fig. 10A). These leukemic proB cells were isolated from the BM of diseased mice, as B220^+^, by MACS-sorting, then cultured in medium containing IL-7 and, later, propagated in IL-7-independent culture conditions^9^. These leukemic *Pax5*^+*/−*^ pro-B cells harboring *Jak3*^V670A^ cells were injected into syngenic mice (n = 10), and the animals were randomized to treatment at day 14 when the disease was confirmed by the presence of blast cells in the PB (Supplementary Fig. 10). Mice were treated with two doses of the anti–IL-6 antibody at 10 mg/kg, 3 days apart. FACS analysis of the PB was used to verify disease remission during therapy. Anti–IL-6 antibody therapy provided no significant survival advantage and none of all mice treated (*n* = 5) showed a decrease in the percentage of blast cells (Supplementary Fig. 10B), even though the anti-IL-6 treatment significantly reduced the levels of IL-6 in the serum of treated mice (Supplementary Fig. 10C and Supplementary Table S5). However, an important caveat to take into account when interpreting this result is the fact that we found that the standard culture conditions with IL-7 used to isolate leukemic proB cells from mice abolished the IL-6 production both in vitro (Supplementary Fig. 11 and Supplementary Table S5) and in vivo (Supplementary Fig. 10C and Supplementary Table S5). These findings are in agreement with previous observations showing that cytokine-mediated survival signals do not compete between them^32^. These observations explain why the use of anti–IL-6 antibody therapy was not able of modifying the course of the disease, and underscore the limitations of the use of ex vivo functional studies involving leukemic pro-B cell in vitro expansion and bone marrow transplantation to identify how inflammatory signaling affects B-ALL development. Therefore, as previously suggested^33^, transplant-based mouse models need to be viewed with particular caution when trying to dissect the role of cytokine factors during oncogenic transformation.

### IL-6 inhibition therapeutically targets *Pax5*-dependent B-ALL in vivo

**Figure 5:**
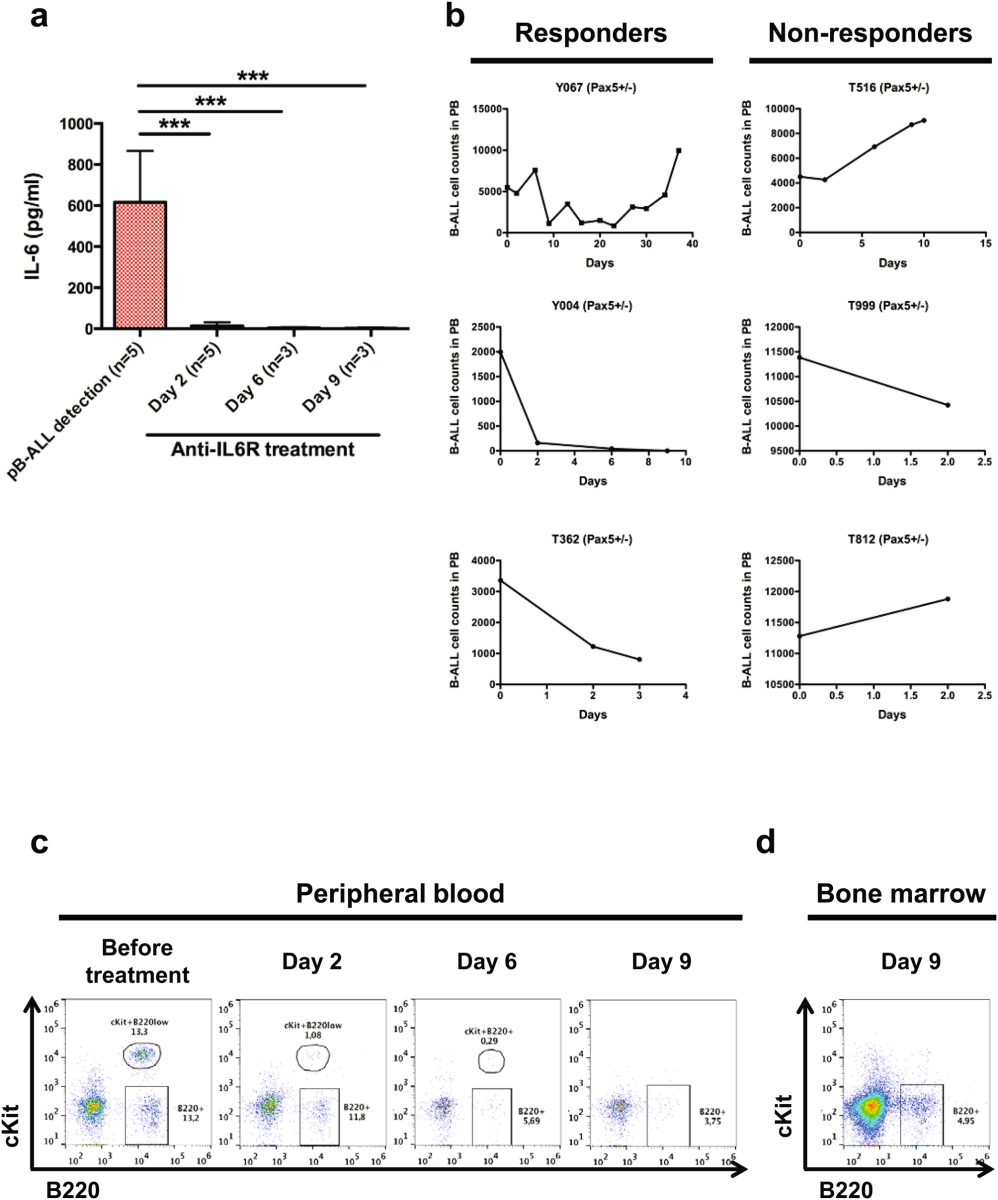
Anti-IL-6 antibody is able to eliminate blast cells in *Pax5*^+*/-*^ leukemic mice. (**a**) IL-6 serum levels in *Pax5*^+*/-*^ leukemic mice treated with anti-IL-6 (n = 5–3). Error bars represent the mean and SD. For the significant differences, an unpaired t-test was used (***; p-value < 0.0001). (**b**) B-ALL cells were decreased in 3 out of 6 *Pax5*^+*/-*^ mice after anti-IL-6 treatment. (**c–d**) FACs analysis of PB and BM showing the reduction of blast cells in a responder *Pax5*^+*/-*^ leukemic mouse due to anti-IL-6 treatment.

Our previous results indicate that a complete understanding of how the modulation of the inflammatory signaling affects *Pax5* mutant B-ALL can only come from the analysis of intact, unmanipulated animals. Thus, we next explored if the blockade of IL-6 could modify the course of a native non-transplant *Pax5*-dependent B-ALL disease. To this aim, *IL-6*^+/+^/*Pax5*^+*/-*^ mice were randomized to treatment with either immunoglobulin G (IgG) control antibody or an anti-IL-6 antibody, twice a week (10 mg/kg) once the B-ALL disease appeared as a result of natural infection exposure^9^, as confirmed by the presence of blast cells in the PB (Supplementary Fig. 12A). The IgG control-treatment did not modify the course of the disease with independence of the percentage of blasts at diagnosis. However, we found that the anti–IL-6 antibodies abolished the increase in IL-6 serum levels characteristic of *Pax5*-dependent B-ALL (Fig. 5a). Blockade of IL-6 in vivo reduced disease progression in 100% of the anti-IL-6-treated mice when the percentage of blast cells in PB was lower than 40% at the time of treatment (Fig. 5b-c and Supplementary Fig. 12B-D). However, disease progression could not be modified by IL-6 inhibition when the percentage of blast cells in PB was higher than 70% at the time of treatment (Fig. 5b-c and Supplementary Fig. 12E-G). In order to further clarify if the relapse (the reoccurrence of disease in the responders) after IL-6 inhibition is driven by pre-existing leukemic cells or by the selection of IL6-resistant clones through the acquisition of new mutations, we next performed whole exome sequencing of the 6 *IL-6*^+*/*+^/*Pax5*^+*/-*^ tumors before anti-IL-6-treatment and after relapse. Tumor DNA was derived from whole leukemic PB, while tail DNA of the respective mouse was used as reference germline material. Leukemias at relapse showed similar recurrent mutations than leukemias before anti-IL-6-treatment (Supplementary Fig. 13 and Supplementary Table S6). Thus, these data further suggest that *IL-6* retains driver functions in established leukemia and demonstrate the application of this in vivo native assay to identify the importance of players relevant for B-ALL development. These results show that IL-6-neutralizing antibodies may be useful therapeutic options in the treatment of *Pax5*-dependent leukemias.

## Discussion

### Proleukemic inflammatory environment in *Pax5*-mutant B-cell leukemogenesis

Our results uncover an essential mechanism involving the proinflammatory cytokine IL-6 in sustaining *Pax5*-dependent B-ALL development. Abnormal profiles of inflammatory markers, including higher concentrations of IL-6, IL-17, and IL-18, have already been detected in neonatal blood spot samples of children who later developed B-cell precursor ALL^34^, and in vitro studies have also shown that pro‐inflammatory cytokines like IL-6/IL-1β/TNFα^35^ or TGF-β dependent signaling^36,37^, can predispose pre‐leukemic B cells to malignant transformation. However, the real contribution of these inflammatory pathways to B-ALL development remains unclear. In this study, we examined the impact of inflammation on the conversion of *Pax5*^+*/-*^ preleukemic cells. *PAX5* is frequently mutated in a large percentage of B-ALL cases^3–6^, but these mutations can also be present in normal children who will never develop B-ALL^7,8^, very much like the *ETV6-RUNX1* translocation only causes B-ALL in a small percentage of carriers^38^. It is known that infection exposure pushes the progression from preleukemic state to full-blown B-ALL in a significant fraction of *Pax5*^+*/-*^ or *ETV6-RUNX1*^+^ cases^9,13,21^. Here, we have examined how the inflammatory profile changes the behavior of preleukemic cells in a *Pax5*^+*/-*^ mouse model. We identify IL-6 as a key cytokine whose expression and secretion by mouse leukemic B-cells is induced when *Pax5* is lost during the course of B-ALL development. Serum IL-6 levels and the expression of IL-6 are significantly upregulated in both *Pax5*-deficient leukemic and healthy B-cells. The presence of IL-6 was further confirmed in serum of B-ALL patients where *PAX5* function is lost. Mechanistically, we show unambiguously by a genetic approach in the *Pax5*^+*/-*^ mouse that impairment of IL-6 signaling delays B-ALL development, hence confirming the essential contribution of this pathway as a feedback loop supporting B-ALL development. An important aspect that should be taken into consideration is that, although our data show that Pax5 indeed regulates IL6 expression, it cannot be rouled out that the microenvironment, following infections and/or leukemia, might also contribute to the observed IL6 increase. Nevertheless, our findings, taken together, demonstrate that *Pax5*-dependent B-ALLs are profoundly affected by the proleukemic inflammatory environment in which leukemic progenitor cells reside, and also support the view that increased levels of the pro-inflammatory cytokine IL-6 are an essential trigger of the B-ALL disease observed in *Pax5*^+*/-*^ mice.

### IL-6 signaling as a target for *Pax5*-mutant B-ALL therapy

IL-6 has been previously implicated in the pathogenesis of hematological malignancies, like multiple myeloma^39^, Hodgkin’s lymphoma^40^, CML^41,42^, CMML^43^, and solid cancers, like breast^44^, prostate^45^, and pancreatic cancer^46^. Likewise, it has been suggested that increased autocrine IL-6 expression in *PAX5* − mantle cell lymphoma (MCL) cells may contribute to the reduction in *TP53* gene expression and could provide survival advantages to lymphoma cells^47^. In this study, we identify IL-6 as a major player in *Pax5*-dependent B-ALL pathogenesis, and we demonstrate that *Pax5* activity dictates the level of IL-6 produced by both mouse and human leukemic B-cells, regulating *IL-6* mRNA levels through BCL6 and the STAT5 pathway, which are two well known transcriptional regulators of the *IL-6* gene^24,48^. Furthermore, we find that disruption of the IL-6 loop through genetic downregulation of the *IL-6* gene in *Pax5*^+*/-*^ mice significantly delays B-ALL onset, and that blocking IL-6 signaling kills *Pax5*-dependent B-ALL in unmanipulated animals. All these results show that the IL-6 signaling pathway represents a therapeutic vulnerability in *Pax5*-dependent B-ALL, and that its targeting could be a promising therapy for this disease, which could also be extended to other hematological diseases, taking into account the high frequency of somatic *PAX5* losses-of-function in different types of B cell leukemias.

## Material and methods

### Mouse model for natural infection-driven leukemia

*Pax5*^+*/-*^ mice^49^ were crossed with *IL-6*^*-/-*^ mice^50^ to generate *Pax5*^+*/-*^*/IL-6*^+*/-*^ mice. These *Pax5*^+*/-*^*/ IL-6*^+*/-*^ and *Pax5*^+*/-*^ mice were bred and maintained in the SPF area of the animal house until the moment when they were relocated to an environment where natural infectious agents were present, as previously described^9^. Animal studies were performed in accordance with relevant guidelines and regulations and approved by the pertinent institutional committees of both University of Salamanca and Spanish Research Council (CSIC). *IL-6*^+*/-*^*, IL-6*^*-/-*^*, Pax5*^+*/-*^*/IL-6*^+*/-*^, and *Pax5*^+*/-*^ mice of a mixed C57BL/6 × CBA background were used in this study, with approximately equal representation of both males and females. For the experiments, *IL-6*^+*/-*^*, IL-6*^*-/-*^*, Pax5*^+*/-*^*/IL-6*^+*/-*^, and *Pax5*^+*/-*^ of the same litter were used. When the animals showed evidences of illness, they were humanely killed, and the main organs were extracted by standard dissection. All major organs were macroscopically inspected under the stereo microscope, and then representative samples of tissue were cut from the freshly dissected organs, and were immediately fixed. Differences in Kaplan–Meier survival plots of transgenic and WT mice were analyzed using the log-rank (Mantel-Cox) test. Also samples from *Pax5*^+*/*+^*;Sca1-BCR-ABLp190, Pax5*^+*/-*^*;Sca1-BCR-ABLp190, Pax5*^+*/*+^;*Sca1-ETV6-RUNX1 and Pax5*^+*/-*^;*Sca1-ETV6-RUNX1* leukemic mice were used to measured cytokine serum levels.

### Flow cytometry and cell sorting

Flow cytometry and cell sorting was carried out as previously described^9,13^. Total mouse BM cells were obtained by washing the long bones with PBS with 1% FCS, using a 27-G needle. Cells were as well collected from peripheral blood, and also from the thymus and spleen after disrupting these organs by passing them through a 70-μm cell strainer. Erythrocytes were osmotically lysed using RCLB buffer, and nucleated cells were then washed with PBS–1% FCS. Cells were stained with the appropriate antibodies against the indicated cellular markers for 20 min at 4 °C, washed once with PBS–1% FCS, and finally they were resuspended in PBS–1% FCS with 10 μg/mL propidium iodide (PI) to exclude dead cells during data acquisition; this was performed in an AccuriC6 Flow Cytometer, and data files were analyzed using Flowjo software. For this analysis, gates were set by employing the commonly used forward and perpendicular light-scattering properties of mouse hematopoietic cells, and the specific fluorescence of the staining dyes used [FITC, PE, PI, and APC excited at 488 nm (0.4 W) and 633 nm (30 mW), respectively]; an example of such a gating strategy is shown in Supplementary Fig. 14. The nonspecific binding of staining antibodies to the Fc receptors of immune cells was prevented by incubating the samples with anti-CD16/CD32 Fc-block solution (clone 2.4G2, cat. #553,142, BD Biosciences) for 20 min at 4 °C, previously to the addition of the staining antibodies. For each sample tube, a minimum of 50,000 living (i.e., PI-negative) cells were acquired and analyzed.

The antibodies used for flow cytometry were all from BD Biosciences: anti-B220 (clone RA3-6B2, cat. #103,212, used in 1:100 dilution), CD4 (clone RM4-5, cat. #100,516, used in 1:250 dilution), CD8a (clone 53–6.7, cat. #100,708, used in 1:250 dilution), CD11b/Mac1 (clone M1/70, cat. #553,310, used in 1:200 dilution), CD19 (clone 1D3, cat. #152,404, used in 1:100 dilution), CD117/c-Kit (clone 2B8, cat. 105,807, used in 1:200 dilution), Ly-6G/Gr1 (clone RB6-8C5; cat. #108,412, used in 1:100 dilution), IgM (clone R6-60.2, cat. #406,509, used in 1:100 dilution) and CD25 (clone PC61, cat. #553,866, used in 1:100 dilution). The gating strategy is exemplified in Supplementary Fig. 14.

### Real- time PCR quantification (Q-PCR) of* mIL-6, mBcl6* and *mBlnk*

Real- time PCR quantification (Q-PCR) was carried out as previously described^9,13^. We analyzed the expression of *mIL-6, mBcl6 and mBlnk* in leukemic *Pax5*^+*/-*^ cells as well as in healthy *Pax5*^+*/-*^ and WT proB cells by Q-PCR as follows: cDNA was synthesized using reverse transcriptase (Access RT-PCR System; Promega, Madison, WI) and genomic DNA was removed by DNAase treatment (Roche, 04 716 728 001). Real-time PCR reactions were performed in an Eppendorf MasterCycler Realplex machine. Commercially available assays for quantitative PCR from IDT (Integrated DNA Technologies) were used: *mIL-6* (Assay ID: Mm.PT.58.10005566), *mBcl6* (Assay ID: Mm.PT.58.32669842), *mBlnk* (Assay ID: Mm.PT.58.7821272) and *Gapdh* (Assay ID: Mm.PT.39a.1). Probes were specifically designed to prevent detection of genomic DNA by PCR. Measurement of *Gapdh* gene product expression was used as an endogenous control and the total bone marrow of a WT mouse was used as a reference to calculate the fold change. We also analyzed the murine IL6 expression in healthy precursor *Pax5*^*-/-*^ B cells using a qRT-PCR assay (Roche; cat. no. 04685032001) recognizing the Gene identified as ENSMUSG00000025746 (GRCm38.p6), and detecting all the existing 4 transcript isoforms IL6-201 (ENSMUSG00000026845.11), IL6-202 (ENSMUSG000000195978.4), IL6-203 (ENSMUSG000000199183.4), IL6-204 (ENSMUSG000000199765.1). The Taqman assay used the following primers: mIL6-L6 (5′-GCTACCAAACTGGATATAATCAGGA-3′) and mIL6-R6 (5′- CCAGGTAGCTATGGTACTCCAGAA-3′) and in this case measurement of *Hprt* gene product expression was used as an endogenous control and murine mesenquimal stem cells (mMSC) were used as a reference to calculate the fold change. All samples were run in triplicate. The comparative CT Method (ΔΔCt) was used to calculate relative expression of the transcript of interest and a positive control. The change in threshold cycle (ΔCt) of each sample was calculated as the Ct value of the tested gene (target) minus the Ct value of *Gadph* (endogenous control). The ΔΔCt of each sample was obtained by subtracting the ΔCt value of the reference from the ΔCt value of the sample. The ΔCt reference value used was the ΔCt obtained from total BM of a WT mouse. The fold change in each group, calculated as 2–ΔΔCt sample, was compared.

### Histology

Histology was carried out as previously described^9,13^. Animals were sacrificed by cervical dislocation; tissue samples were formalin-fixed and included in paraffin. Pathology assessment was performed on hematoxylin–eosin stained sections under the supervision of Dr. Oscar Blanco, an expert pathologist at the Salamanca University Hospital.

### Quantification of cytokine levels in serum

Quantification of cytokine levels in serum was carried out as previously described^41^. Serum cytokine levels were analysed using The Cytometric Bead Array immunoassay system (CBA) (BD Biosciences) which assesses simultaneously IL-2, IL-4, IL-6, IL-10, IL-17A, TNF alpha and IFN gamma in serum from the mice (Mouse Th1 Th2 Th17 Cytokine Kit #560,485; BDB); and serum from the patients and controls (Human Th1 Th2 Th17 Cytokine Kit #560,484; BDB). Human studies were performed in accordance with relevant guidelines and regulations and approved by the *Comisión de donación de muestras del Institute Josep Carreras (IJC)*. Serum was obtained from children and adults and all participants or their legal guardians provided written informed consent to take part in the study. Data acquisition was performed on a FACSCanto II flow cytometer (BDB) using the FACSDiva™ software program (BDB). For the evaluation of cytokine serum levels or cytokine secretion into the culture supernatants, 50 µl of serum was collected. Briefly, 50 µl of the serum was incubated at room temperature for 2 h at room temperature (RT) with 50 µl of anticytokine MAb-coated beads and with 50 µl of the appropriate phycoerythrin (PE)- conjugated anticytokine antibody detector. After this incubation period, samples were washed once (5 min at 200 g) in order to remove the excess of detector antibodies. Immediately afterwards, data acquisition was performed on a FACSCanto II flow cytometer (BDB) using the FACSDiva™ software program (BDB). During acquisition, information was stored for 3,000 events corresponding to each bead population analysed per sample (total number of beads > 9,000). For data analysis, FCAP Array Software v3.0 program (BDB) was used.

### Anti-IL-6R treatment

Anti-IL-6R antibody (Tocilizumab) was obtained from Chugai Pharmaceuticals Co. Ltd. (Shizuoka, Japan). Anti-IL-6R antibody was intraperitoneally administered at 10 mg/kg twice a week. Treatments were started after the establishment of B-ALL.

### ProB cell culture

ProB cell culture was carried out as previously described^9,13^. Pro-B cells were purified from BM using magnetic-activated cell sorting, selecting with anti-B220 beads (Milteny Biotec). Pro-B cells were maintained and expanded by culturing them in Iscove’s Modified Dulbecco’s Medium (IMDM) supplemented with 50 μM β-mercaptoethanol, 1 mM L-Gln, 2% heat-inactivated FCS, 1 mM penicillin–streptomycin (BioWhittaker), 0.03% (w/v) primatone RL (Sigma), and 5 ng/ml mrIL-7 (R&D Systems), in the presence of Mitomycin C-treated ST2-feeder cells. Tumor pro-B cells that could grow independently of IL-7 were grown in the same medium without this cytokine.

### Transplantation

Transplantation was carried out as previously described^9,13^. IL-7-independent leukemic pro-B cells were intravenously injected into 12-week-old male syngenic mice (C57BL/6 × CBA) that had previously been sublethally irradiated (4 Gy). Leukemia development in the injected mice was followed by regular analysis of peripheral blood, until the moment when leukemic blasts were detected in the blood; at this point, animals were treated with anti-IL-6R antibody.

### V(D)J recombination

V(D)J recombination analysis was carried out as previously described^9,13^. Immunoglobulin rearrangements were amplified by PCR using the primers below. Cycling conditions consisted of an initial heat-activation at 95 °C followed by 31–37 cycles of denaturation for 1 min at 95 °C, annealing for 1 min at 65 °C, and elongation for 1 min 45 s at 72 °C. This was followed by a final elongation for 10 min at 72 °C. The following primer pairs were used:

**Table:** 

V_H_J558	forward	CGAGCTCTCCARCACAGCCTWCATGCARCTCARC
	reverse	GTCTAGATTCTCACAAGAGTCCGATAGACCCTGG
V_H_7183	forward	CGGTACCAAGAASAMCCTGTWCCTGCAAATGASC
	reverse	GTCTAGATTCTCACAAGAGTCCGATAGACCCTGG
V_H_Q52	forward	CGGTACCAGACTGARCATCASCAAGGACAAYTCC
	reverse	GTCTAGATTCTCACAAGAGTCCGATAGACCCTGG
DH	forward	TTCAAAGCACAATGCCTGGCT
	reverse	GTCTAGATTCTCACAAGAGTCCGATAGACCCTGG
Cμ	forward	TGGCCATGGGCTGCCTAGCCCGGGACTT
	reverse	GCCTGACTGAGCTCACACAAGGAGGA

### Microarray data analysis

Microarray data analysis was carried out as previously described^9,13^. The total RNA was first isolated using TRIzol (Life Technologies), and then it was subjected to purification with the RNeasy Mini Kit (Qiagen) using also the On-Column DNase treatment option. Quality and quantification of RNA samples were determined by electrophoresis.

Determination of the expression of the different genes in the RNA samples was performed using Affymetrix Mouse Gene 1.0 ST arrays. All bioinformatic analyses of the array data were performed using R^51^ and Bioconductor^52^. First, we applied background correction, intra- and inter-microarray normalization, and expression signal calculation using the microarray analysis algorithm^53–55^, in order to determine the absolute expression signal for each gene in each array. Then, we used the significance analysis of microarray (SAM)^56^ method to identify the gene probe sets with differential expression between experimental and control samples, SAM uses a permutation algorithm to allow to statistically infer the significance of the differential expression, and it provides *P-*values adjusted to correct for the multiple testing problem, by using FDR^57^. An FDR cutoff of < 0.05 was used as a threshold to determine differential expression. The data discussed in this publication have been deposited in NCBI's Gene Expression Omnibus (GEO)^58^ and are accessible through GEO Series accession number GSE154589.

### Enrichment analysis

Enrichment analysis was carried out as previously described^9,13^. In order to identify potential signatures of gene expression associated with different biological processes, gene set enrichment analysis (GSEA) was performed using the MSigDB databases from the Broad Institute (GSEAv2.2.2)^59^ and hallmark collection of gene sets^60,61^.

### Mouse exome library preparation and NGS

Mouse exome library preparation and NGS was carried out as previously described^9,13^. DNA was purified from samples using the AllPrep DNA/RNA Mini Kit (Qiagen) according to the manufacturer’s instructions. The exome library was prepared using the Agilent SureSelectXT Mouse All Exon Kit with some modifications. Exome capture was performed by hybridization to an RNA library according to the manufacturer’s protocol. Then, the captured library was purified and enriched by binding to MyOne Streptavidin T1 Dynabeads (Life Technologies) and posterior off-bead PCR amplification in the linear range. Sequencing (2 × 100 bp) was carried out in a HiSeq2500 (Illumina) using the TruSeq SBS Kit v3 with a 6-bp index read.

### Data analysis

Data analysis was carried out as previously described^9,13^. Fastq files were generated with Illumina BcltoFastq 1.8.4. The alignment of the sequence data to the GRCm38.71 mouse reference genome was performed with BWA version 0.7.4. SAMtools was used for conversion steps and removal of duplicate reads. GATK 2.4.9 was used for local realignment around indels, SNP-calling, annotation, and recalibration. For recalibration, mouse dbSNP138 and dbSNP for the used mouse strains were used as training data sets. The variation calls obtained in this way were then annotated using the v70 Ensembl database with variant effect predictor (VEP), incorporating loss-of-function prediction scores for PolyPhen2 and SIFT. Afterward, the information was imported into an in-house MySQL database for further annotation, reconciliation, and data analysis by complex database queries if required.

Somatic calls were the output from MuTect^62^ and VarScan^63^. For VarScan2 results, false-positive filtering was used as indicated by the author. In order to increase the reliability of the results, only calls having at least a 9% difference in allele frequency between tumor and normal samples were considered for further analysis. Cancer-related genes were singled-out by using the information from the Catalogue of Somatic Mutations in Cancer (COSMIC)^64,65^ after having translated the cancer gene consensus from COSMIC by making use of Ensembl’s BioMart^66^.

### Statistical analysis

The Kruskal–Wallis test followed by Dunn's multiple comparison test was used for multiple groups to interpret differences in IL6 serum levels using the statistical software SPSS 23. Comparisons of survival curves estimated by Kaplan—Meier plots using Graph Pad Prism 5.0 were performed by the log-rank (Mantel-Cox) test. And the differences between two sample groups were made using an unpaired t-test with GrapPad Prism 5.0 software. The level of significance was set at p-value < 0.05.

## Supplementary information

Supplementary Information

Supplementary Information

Supplementary Information

Supplementary Information

Supplementary Information

Supplementary Information

Supplementary Information

## Data Availability

Authors can confirm that all relevant data are included in the paper and/or its supplementary information files. Gene Expression Data are accessible through GEO Series accession number GSE154589.
