# Supplementary material for: Inhibition of inflammatory signaling in Pax5 mutant cells mitigates B-cell leukemogenesis
Source: Sci Rep. 2020 Nov 5;10:19189. doi: 10.1038/s41598-020-76206-y (PMC7644722; doi:10.1038/s41598-020-76206-y)
Supplement: Supplementary file 1 — Supplementary Information [file 41598_2020_76206_MOESM1_ESM.docx]

**SUPPLEMENTAL DATA**

**Inhibition of Inflammatory Signaling in *Pax5* Mutant Cells Mitigates B-cell leukemogenesis**

Marta Isidro-Hernández^1,2*^, Andrea Mayado ^2,3*^, Ana Casado-García^1,2^, Jorge Martínez-Cano^4^, Chiara Palmi^5^, Grazia Fazio^5^, Alberto Orfao^2,3^, Jordi Ribera^6^, Josep Maria Ribera^6,7^ , Lurdes Zamora^6,7^, Javier Raboso-Gallego^1,2^, Oscar Blanco^2,8^, Diego Alonso-López^9^, Javier De Las Rivas ^2,10^, Rafael Jiménez^2,11^, Francisco Javier García Criado^2,12^, María Begoña García Cenador^2,12^, Manuel Ramírez-Orellana^13^, Giovanni Cazzaniga^5^, César Cobaleda^4#^, Carolina Vicente-Dueñas^2#^, and Isidro Sánchez-García^1,2#^

*(*, #) Should be considered equal first authors and senior authors, respectively.*

*^1^ Experimental Therapeutics and Translational Oncology Program, Instituto de Biología Molecular y Celular del Cáncer, CSIC-USAL, Campus M. de Unamuno s/n, Salamanca, Spain;  ^2^ Institute for Biomedical Research of Salamanca (IBSAL), Salamanca, Spain; ^3^Servicio de Citometría, Departamento de Medicina, Biomedical Research Networking Centre on Cancer CIBER- CIBERONC* *(CB16/12/00400), Institute of Health Carlos III, and Instituto de Biología Molecular y Celular del Cáncer, CSIC/Universidad de Salamanca, Salamanca, Spain; ^4^Immune system development and function Unit, Centro de Biología Molecular Severo Ochoa (Consejo Superior de Investigaciones Científicas -Universidad Autónoma de Madrid), Madrid, Spain; ^5^Centro Ricerca Tettamanti, Dept of Medicine, University of Milan Bicocca, Monza, Italy; ^6^Josep Carreras Leukaemia Research Institute (IJC), Badalona;  ^7^ Catalan Institute of Oncology-Germans Trias i Pujol, Badalona; ^8^Departamento de Anatomía Patológica, Universidad de Salamanca, Salamanca, Spain; ^9^ Bioinformatics Unit, Cancer Research Center (CSIC-USAL), Salamanca, Spain; ^10^ Bioinformatics and Functional Genomics Research Group, Cancer Research Center (CSIC-USAL), Salamanca, Spain; ^11^Departamento de Fisiología y Farmacología, Universidad de Salamanca, Edificio Departamental, Campus M. de Unamuno s/n, 37007, Salamanca, Spain; ^12^ Departamento de Cirugía, Universidad de Salamanca, Salamanca, Spain; ^13^Department of Pediatric Hematology and Oncology, Hospital Infantil Universitario Niño Jesús, Universidad Autónoma de Madrid, Madrid, Spain.*

**#Correspondence should be addressed to:**

*César Cobaledas Hernández (cesar.cobaleda@csic.es)*

*Carolina Vicente-Dueñas ([cvd@usal.es](mailto:cvd@usal.es)).*

*Isidro Sanchez-Garcia (isg@usal.es).*

**CONTENTS:**

- **SUPPLEMENTAL TABLES**
- **SUPPLEMENTAL FIGURES**

**SUPPLEMENTAL TABLES**

**Supplementary Table S1.** **Mouse serum samples description.** Genotype and IL6 serum levels of IL6^+/-^, IL6^-/-^, Pax5^+/-^ Non-Leukemic, IL6^+/-^ + Pax5^+/-^ Non-Leukemic, Pax5^+/-^ Leukemic, IL6^+/-^ + Pax5^+/-^ Leukemic, BCR/ABL^p190^ Leukemic BCR/ABL^p190^ + Pax5^+/-^ Leukemic, ETV6-RUNX1 Leukemic and ETV6-RUNX1 + Pax5^+/-^ Leukemic mice and wild-type controls. All the mice have been housed in a conventional facility where the mice are exposed to common infections.

**Supplementary Table S2**. **Human serum samples description**. Demographics, genotype and IL6 serum levels of PAX5^+/-^ (n=6), and healthy controls (n=20).

**Supplementary Table S3. Gene list of differentially expressed genes between leukemic bone marrow *IL6^+/-^+Pax5^+/-^* and *Pax5^+/-^* cells. (196 genes-probesets with FDR = 0.05).** Provided as an Excel file.

**Supplementary Table S4. Gene list of differentially expressed genes between leukemic bone marrow *IL6^+/-^+Pax5^+/-^* B-ALL and WT proB cells. (9160 genes-probesets with FDR = 0.05).** Provided as an Excel file.

**Supplementary Table S5.** **Samples description and IL6 serum levels of Pax5^+/-^ Leukemic mice, mice transplanted with leukemic Pax5 proB cells and *in vitro* samples supernatant from proB cell medium culture to test IL6 levels.** Already known IL6 levels in proB cell medium culture (50pg/mL, 500pg/mL, 5ng/mL and 50ng/mL) were used as positive controls for *in vitro* IL6 determination. NA: not applicable.

**Supplementary Table S6. Somatic mutation identified by whole-exome sequencing in leukemic Pax5+/- mice before anti-IL6 treatment and after anti-IL6 treatment at the time of relapse.**

**SUPPLEMENTAL FIGURES:**

**
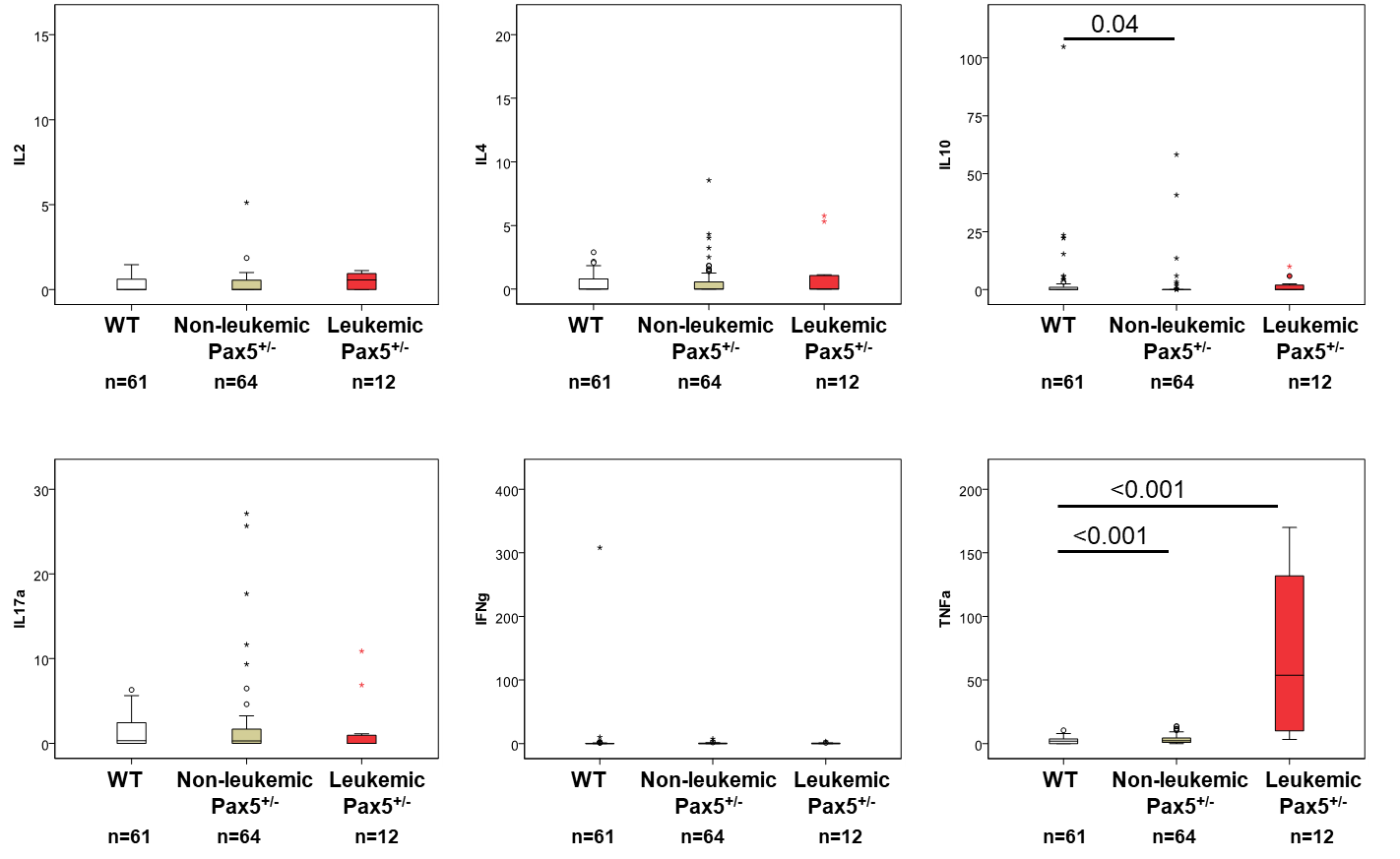
**

**Supplementary Figure 1. Inflammatory cytokines levels in Pax5^+/-^ mice.** IL-2, IL-4, IL-10, IL-17a, IFNγ and TNFα serum levels in non-leukemic (n=64) and leukemic leukemic (n=12) Pax5^+/-^ mice compared with control wild-type mice (n=61). All mice were exposed to an infectious environment as described in the Methods section.

**
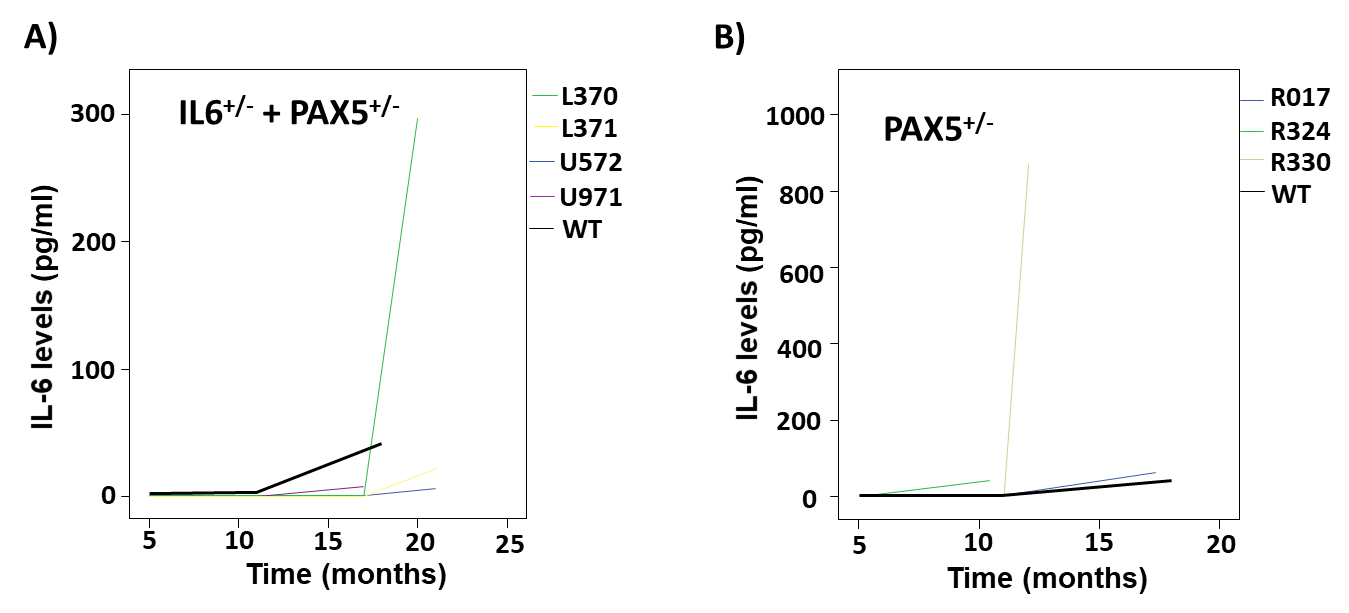
**

**Supplementary Figure 2.** **IL6 serum levels in IL6^+/-^+Pax5^+/-^ and Pax5^+/-^ that develop B-ALL.** IL6 was measured in IL6^+/-^+Pax5^+/-^ (n=4) and Pax5^+/-^ (n=3) mice before and at the time of leukemia onset. Each line corresponds to one individual mouse, whose code name is indicated in the legend.

**
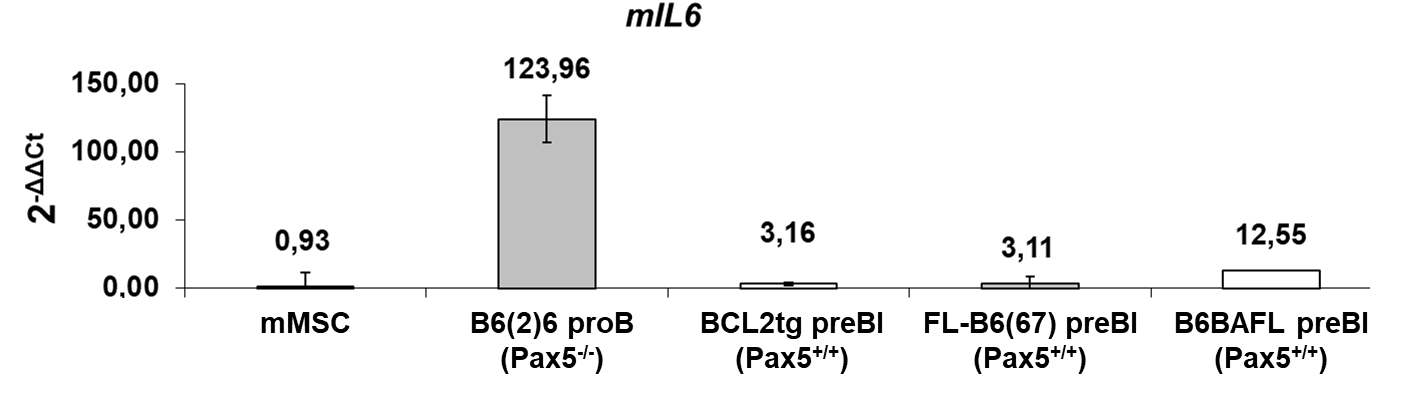
**

**Supplementary Figure 3. Pax5^-/-^ BM precursor B cells overexpress IL-6.** Relative expression of *mIL-6* gene measured by real-time PCR in murine Mesenchymal Stromal Cells (mMSC); BM proB cells knock out for Pax5 gene, isolated from *Pax5^-/-^* mouse, named B6(2)6; BM preBI cells wild type for *Pax5* gene transgenic for BCL2, isolated from transgenic mouse model, named BCL2tg; Fetal Liver preBI cells wild type for *Pax5* gene, isolated from two different B6 mouse models, named FLB6(67) and B6BA FL respectively. The *IL6* expression of mMSC was used as a reference. Error bars represent the mean +/- the standard deviation of at least 3 replicates.


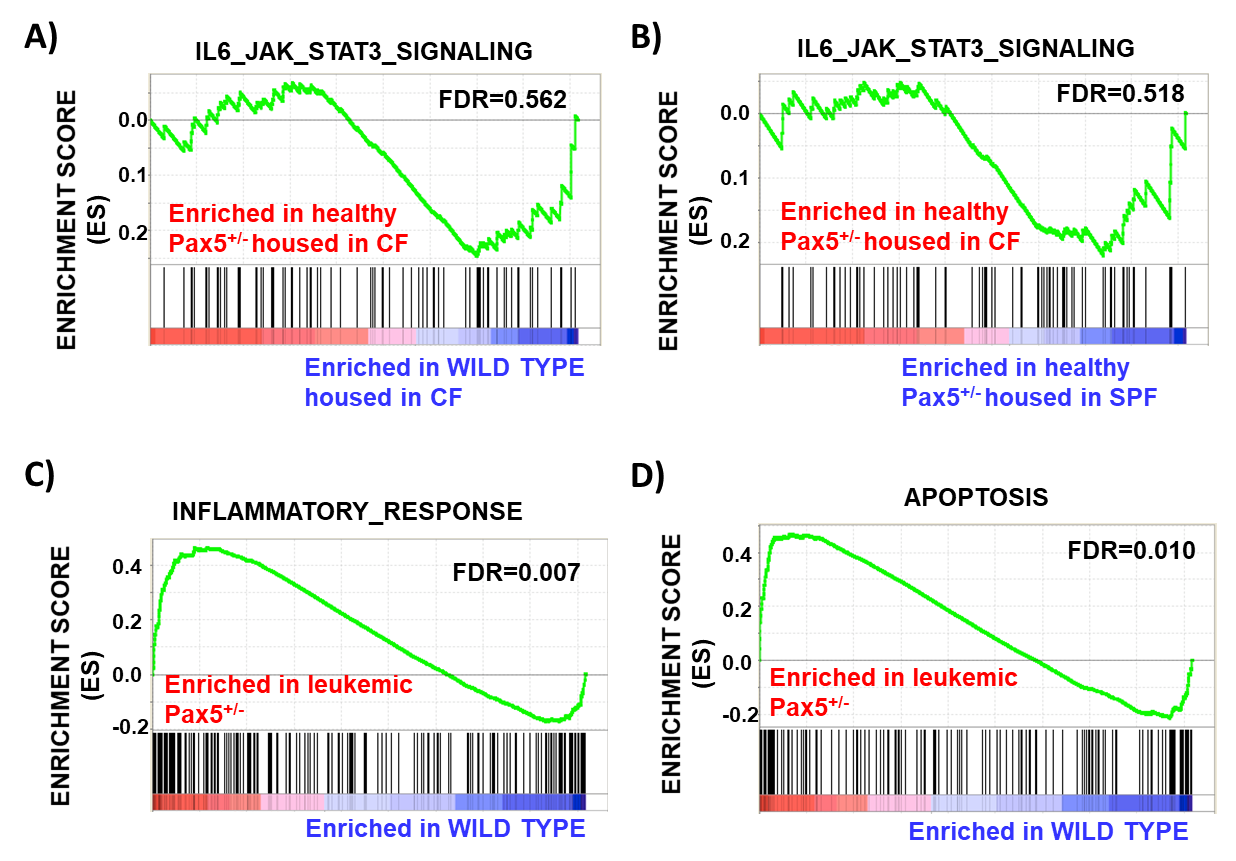


**Supplementary Figure 4. Preleukemic Pax5^+/-^ proB cells are not enriched in IL6 signaling.** Transcriptional signatures within preleukemic (**A-B**) and leukemic Pax5^+/-^ proB cells (**C**) from mice housed in CF conditions (Conventional Facility, where mice are exposed to common infections) were compared with control WT proB cells and Pax5^+/-^ proB cells from mice housed in SPF conditions (Specific Pathogen Free, where mice are not exposed to common infections). GSEA analysis shows that preleukemic Pax5^+/-^ proB cells are not enriched in IL6 signaling (**A-B**) and leukemic Pax5^+/-^ proB cells are significantly enriched in an inflammatory response and apoptosis gene sets **C-D**).

**
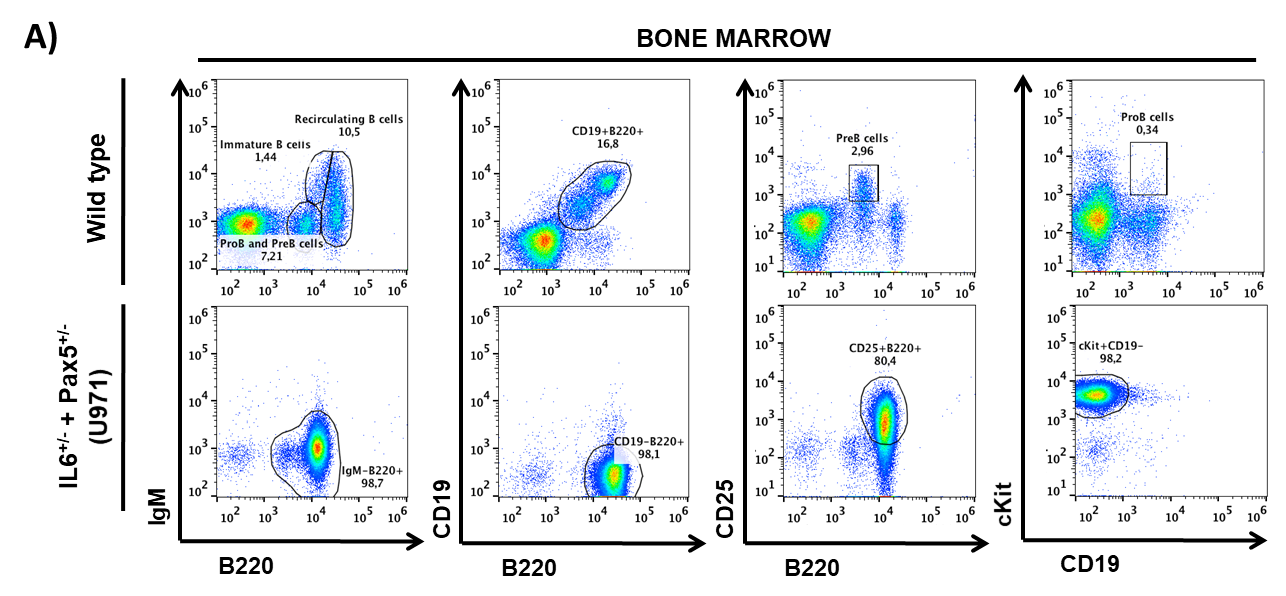
**

**
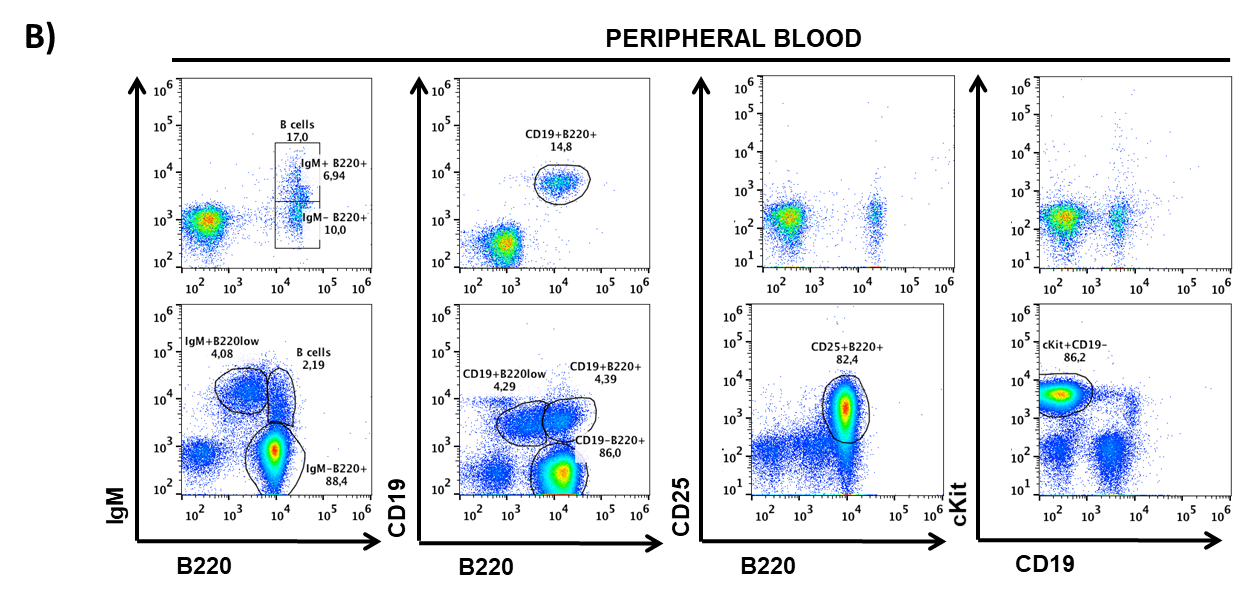
**

**
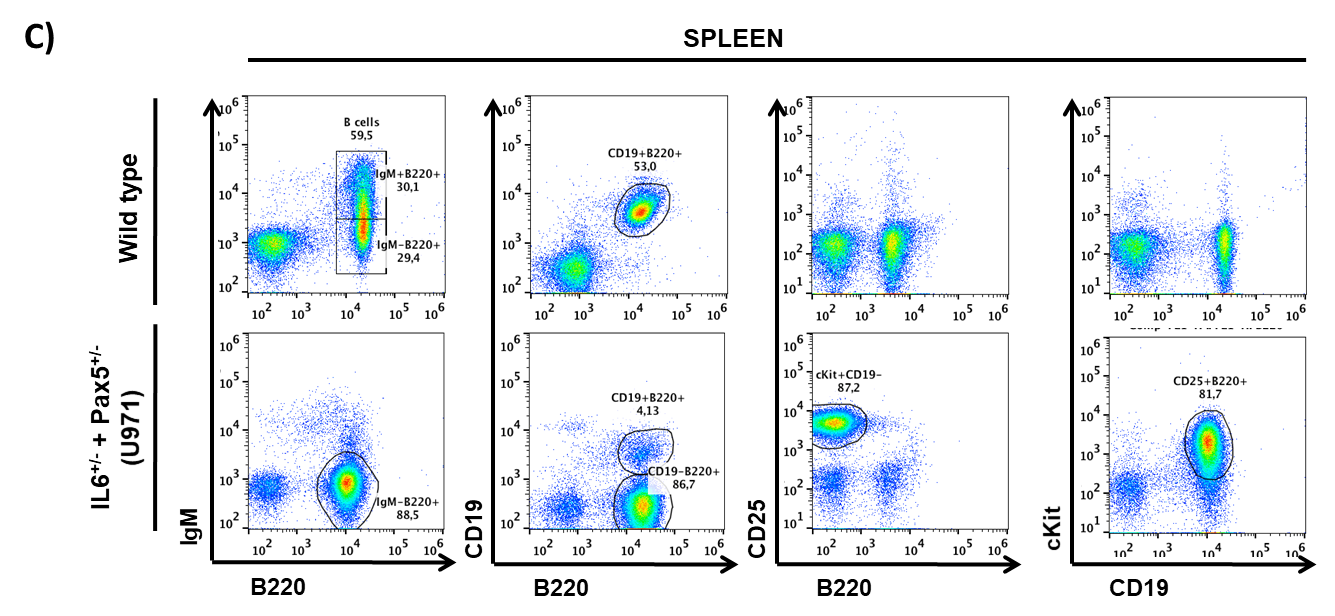
**

**Supplementary Figure 5. Flow cytometry analysis of leukemic IL6^+/-^+Pax5^+/-^ mice.** Representative plots of cell subsets from the bone marrow (**A**), peripheral blood (**B**) and spleen (**C**) show accumulation of blast B cells in leukemic IL6^+/-^+Pax5^+/-^ mouse (U971) compared to age-matched control wild-type littermate.

**
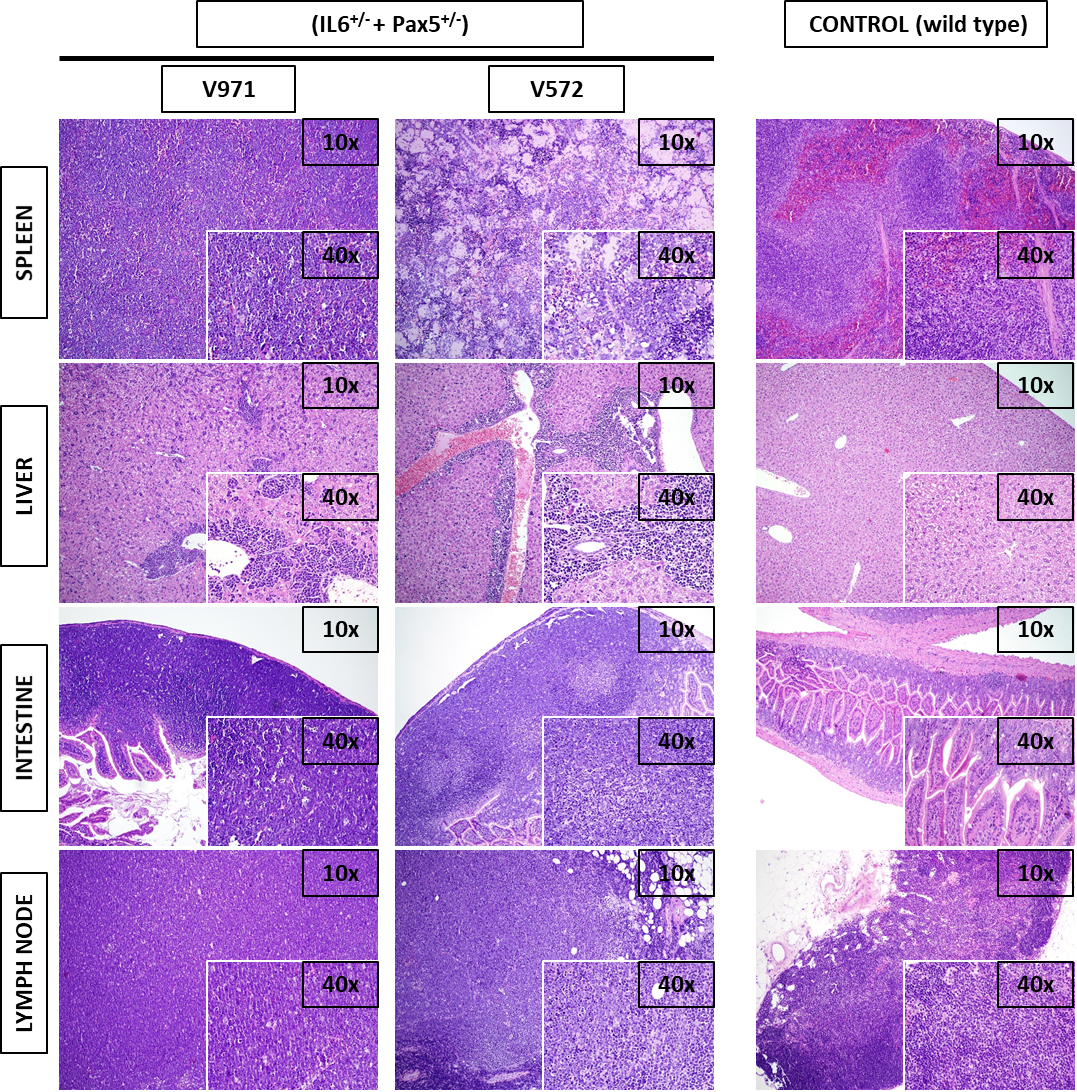
**

**Supplementary Figure 6. B-ALL development in *IL6^+/-^+Pax5^+/-^*** **mice.** Haematoxylin and eosin staining of WT mice and tumor-bearing *IL6^+/-^+Pax5^+/-^* mice, showing infiltrating blast cells in spleen, liver, small intestine and lymph nodes. Loss of normal architecture due to the accumulation of cells morphologically resembling lymphoblasts can be seen in diseased *IL6^+/-^+Pax5^+/-^* mice. Magnification is indicated in each case.

**
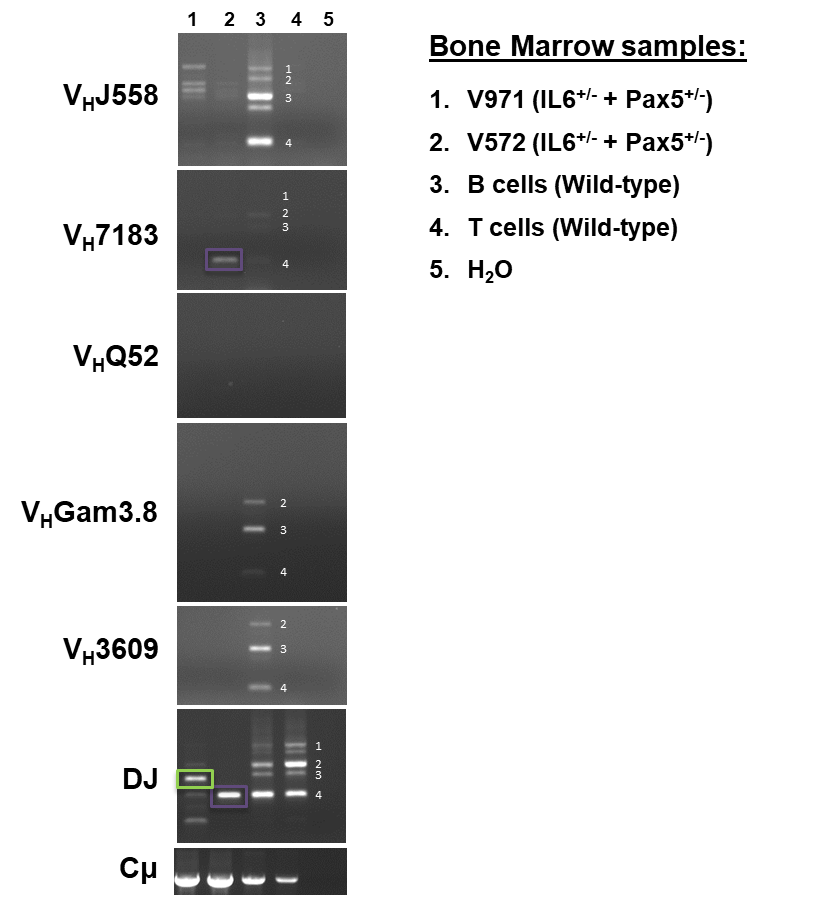
**

**Supplementary Figure 7. *IL6^+/-^+Pax5^+/-^* leukemias are clonal.** Analysis of BCR clonality of leukemias arising in *IL6^+/-^+Pax5^+/-^* mice housed in CF conditions. PCR analysis of BCR gene rearrangements in the bone marrow of diseased mice (V971 and V572). Sorted CD19+ splenic B cells (B cells) of healthy mice serve as a control for polyclonal BCR rearrangements. CD8+CD4+ T cells from the thymus of healthy mice served as a negative control. Bone marrow leukemic cells show increased clonality within their BCR repertoire. Colored boxes denote clonal bands of immunoglobulins. CF: Conventional facility (a facility where mice are exposed to common infections).

**
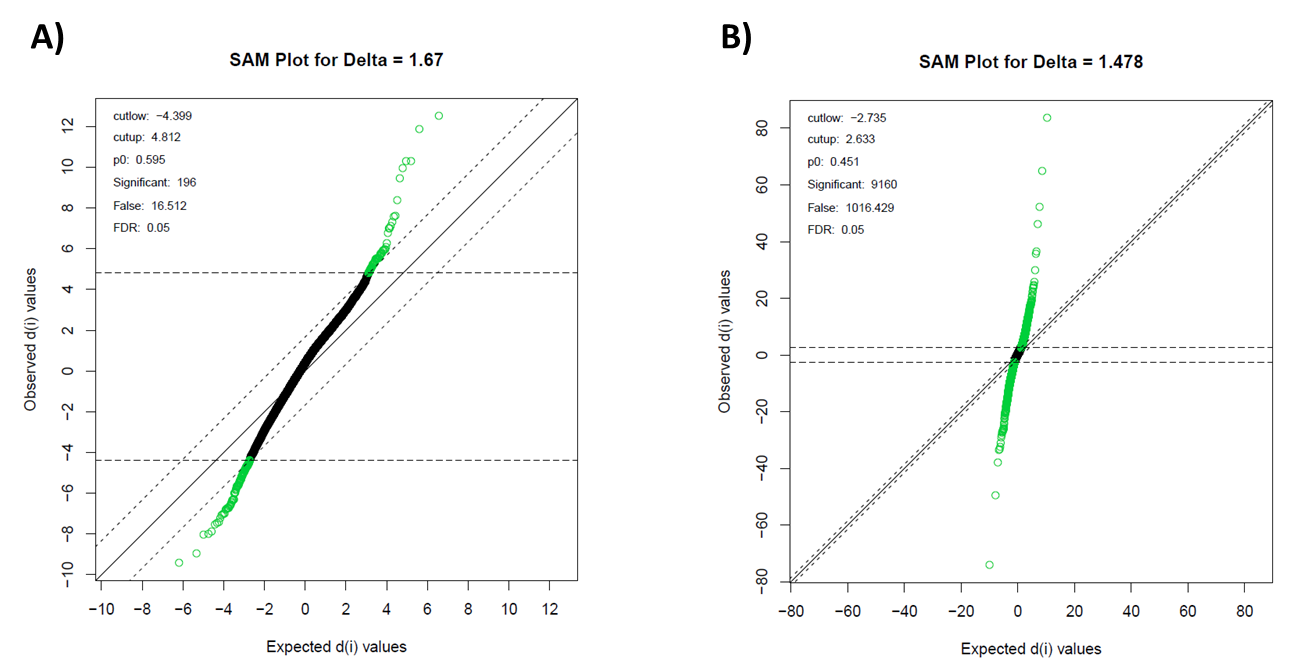
**

**Supplementary Figure 8. Comparison of gene expression profiles between *IL6^+/-^+Pax5^+/-^* B-ALL and Pax5^+/-^ B-ALL (A) and between *IL6^+/-^+Pax5^+/-^* B-ALL and WT proB cells (B).** SAM plot depicting the observed d-statistic versus the null distribution. The absence of a typical pronounced S-shape indicates that the difference between expression patterns in *IL6^+/-^ + Pax5^+/-^* B-ALL and *Pax5^+/-^* B-ALL is weak (**A**) in comparison to observed pronounced S-shape when comparing with healthy WT proB cells (**B**). Gene expression profile of *IL6^+/-^+Pax5^+/-^* and *Pax5^+/-^* B-ALL are quite similar with just 196 probe-sets differentially expressed (**A**) in contrast to the huge differences in terms of gene expression between *IL6^+/-^+Pax5^+/-^* B-ALL and healthy WT proB cells (**B**) with 9160 probe-sets differentially expressed. FDR: 0.05.

**
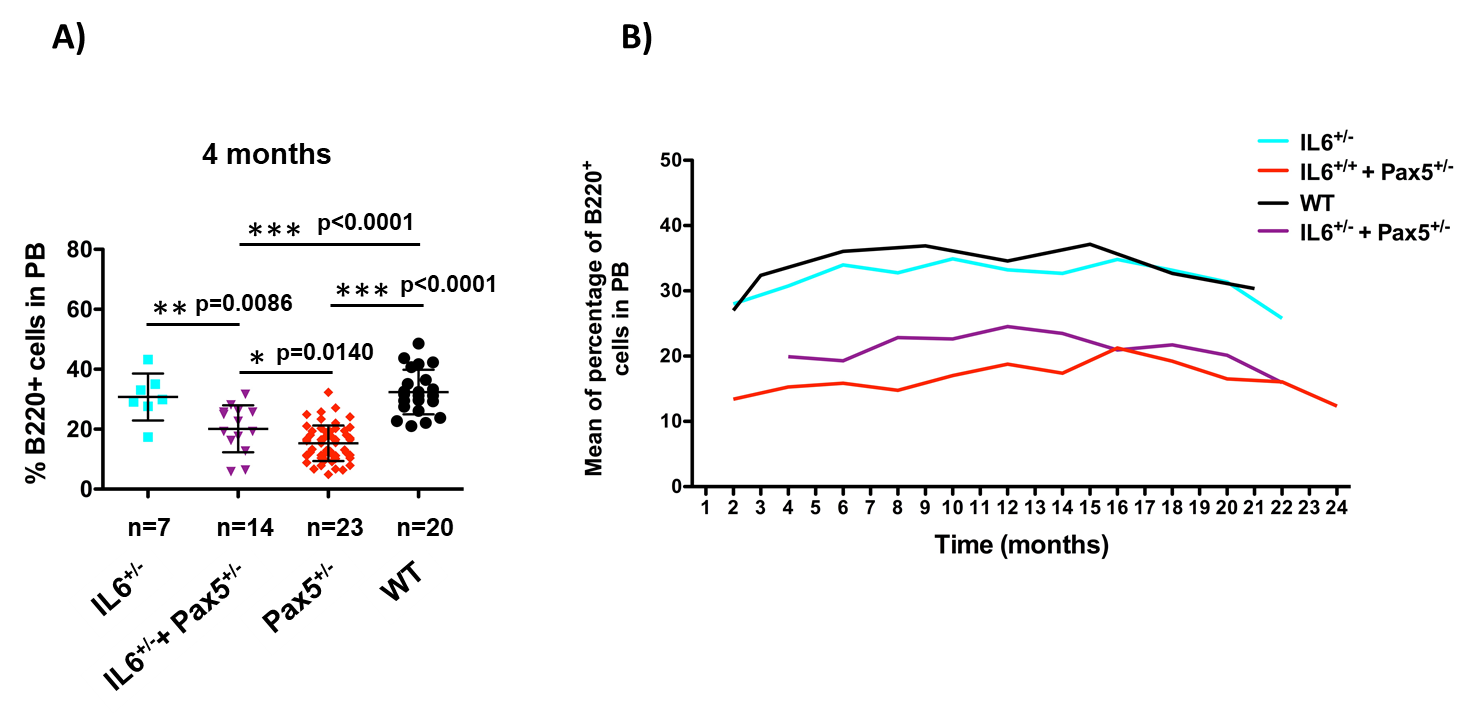
**

**
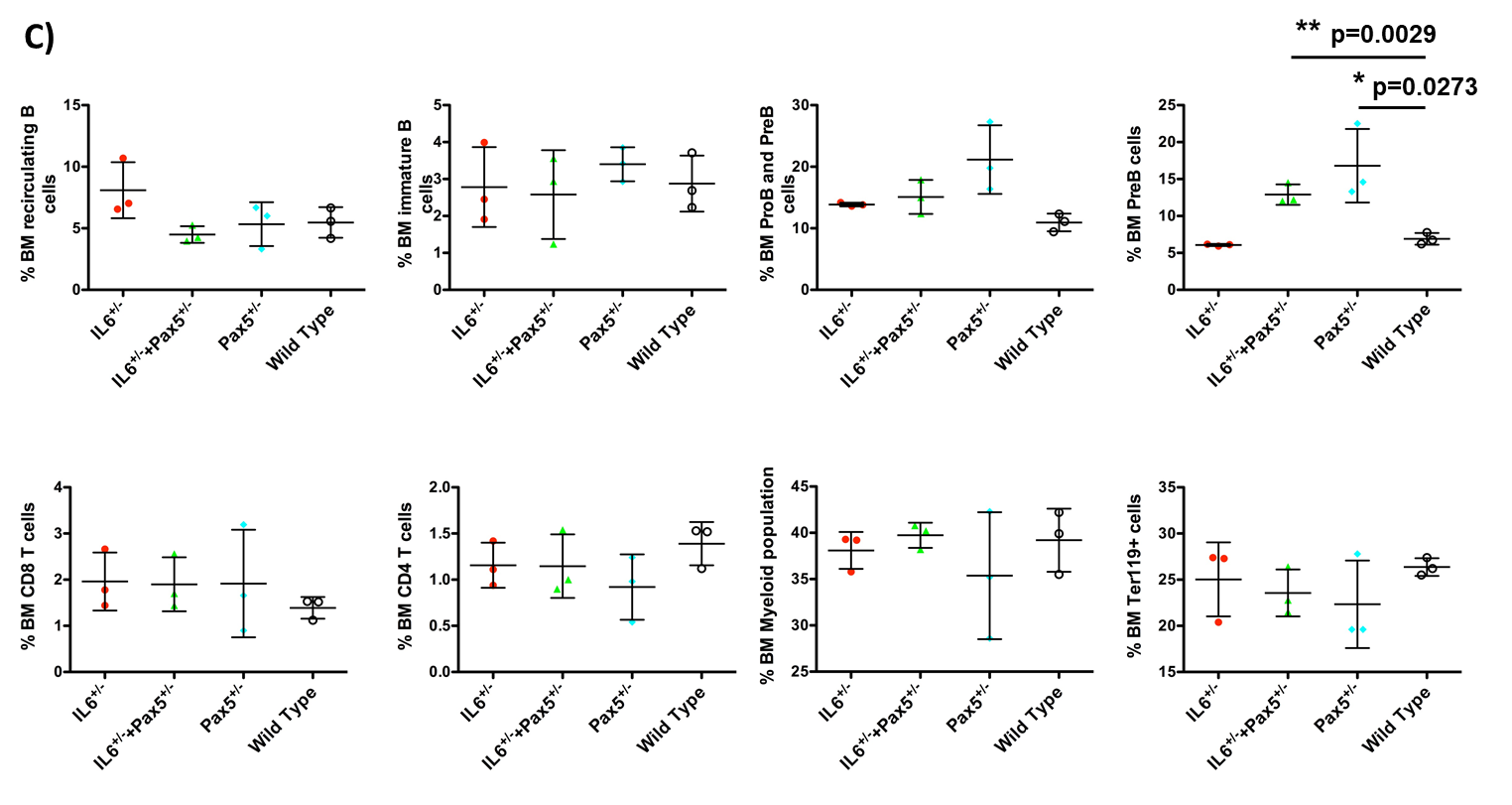
**

**Supplementary Figure 9. IL6^+/-^+ Pax5^+/-^ mice present similar B cell defects that Pax5^+/-^ mice. A**) B220+ cells in PB in pre-leukemic mice at 4 months old. **B**) Evolution of PB B cells in IL6^+/-^+ Pax5^+/-^ mice along time and compared with IL6^+/-^, Pax5^+/-^ and WT mice **C**) BM hematopoietic populations in pre-leukemic IL6^+/-^ + Pax5^+/-^ and compared with IL6^+/-^; Pax5^+/-^ and WT mice. Error bars represent the mean and SD. For the significant differences, an unpaired t-test was used.


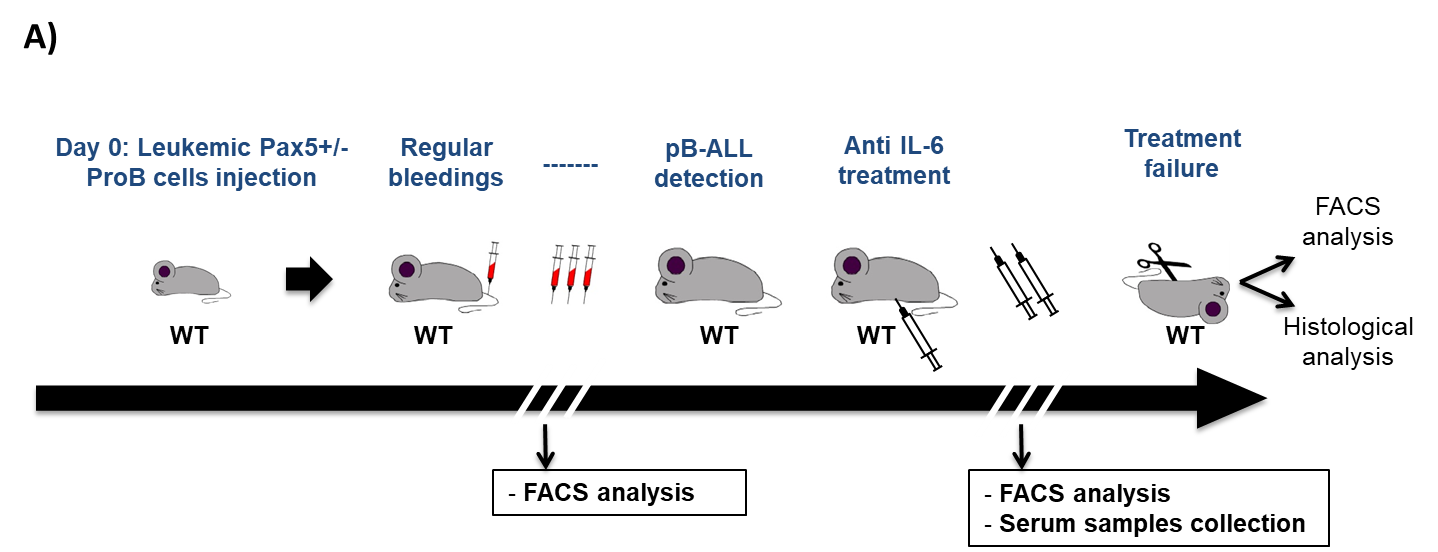


**
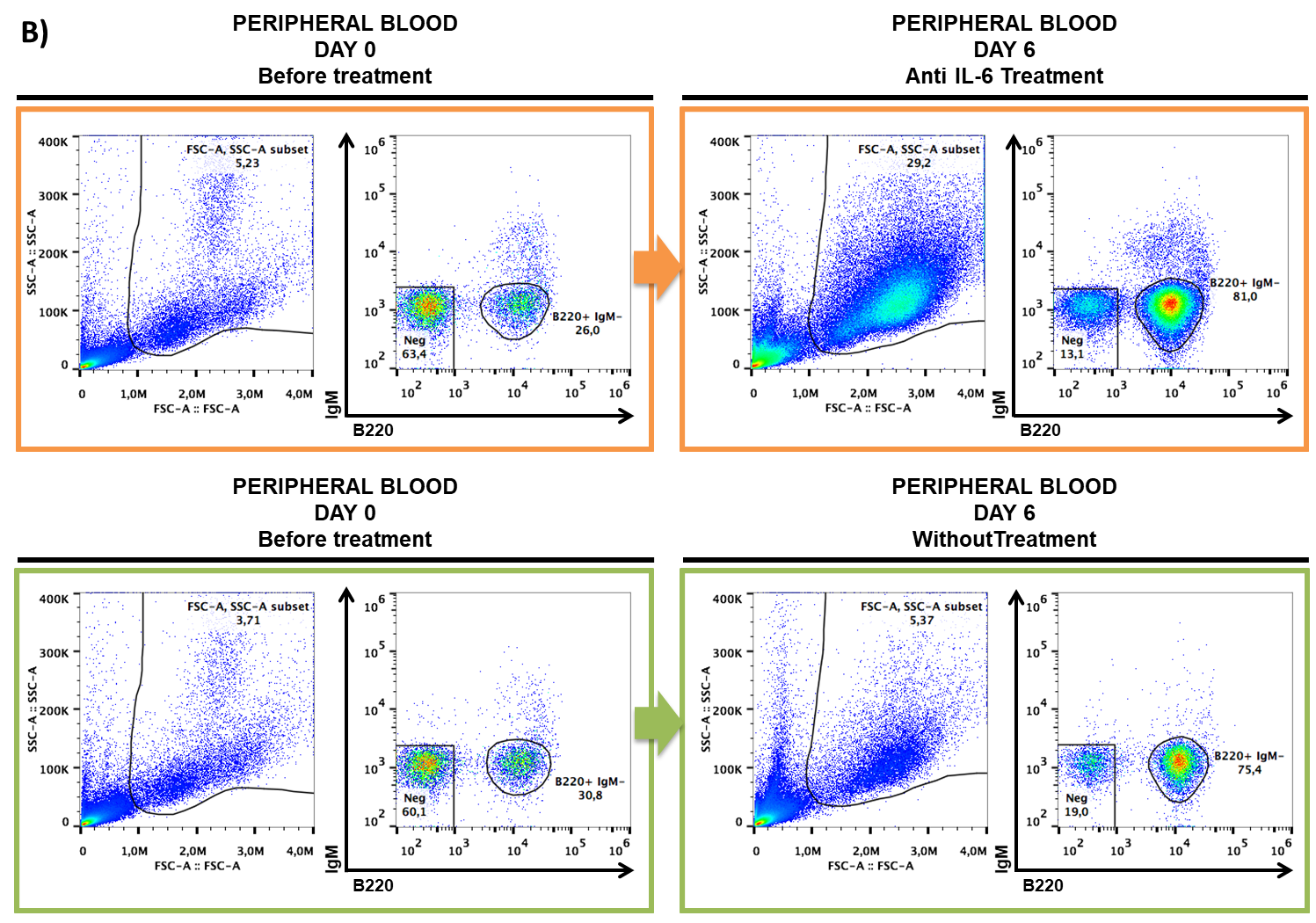
**

**
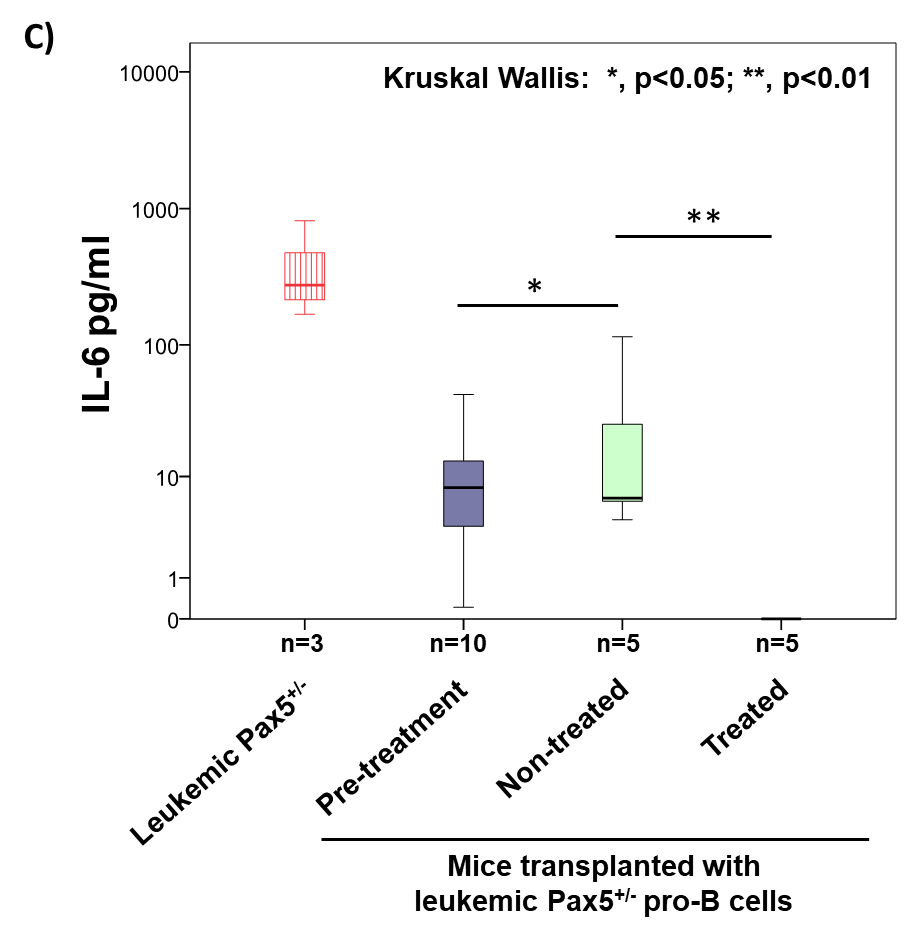
**

**Supplementary Figure 10. Anti-IL6 treatment into transplanted leukemic WT mice.**

**A)** Experimental set-up of the anti-IL6 treatment into transplanted leukemic WT mice. Sub-lethally irradiated WT mice (n=10) were injected with leukemic Pax5^+/-^ ProB cells and, after engraftment (14 days after the injection of leukemic Pax5+/- ProB cells), half of them were treated with Anti-IL6 antibody (n=5) (10 mg/kg twice a week). Serum samples were collected before and after anti-IL6 treatment. **B)** Example of PB cytometry analysis of transplanted mice before and after Anti-IL6 treatment. **C)** IL6 serum levels in mice transplanted with leukemic Pax5+/- proB cells before and after Anti-IL6 treatment. Sera from leukemic Pax5^+/-^ mice were used as a positive control. Notched-boxes extend from the 25th to the 75th percentile values; the lines in the middle and vertical lines correspond to median values and the 10th and 90th percentiles, respectively. The Kruskal–Wallis test was used to interpret differences.

**
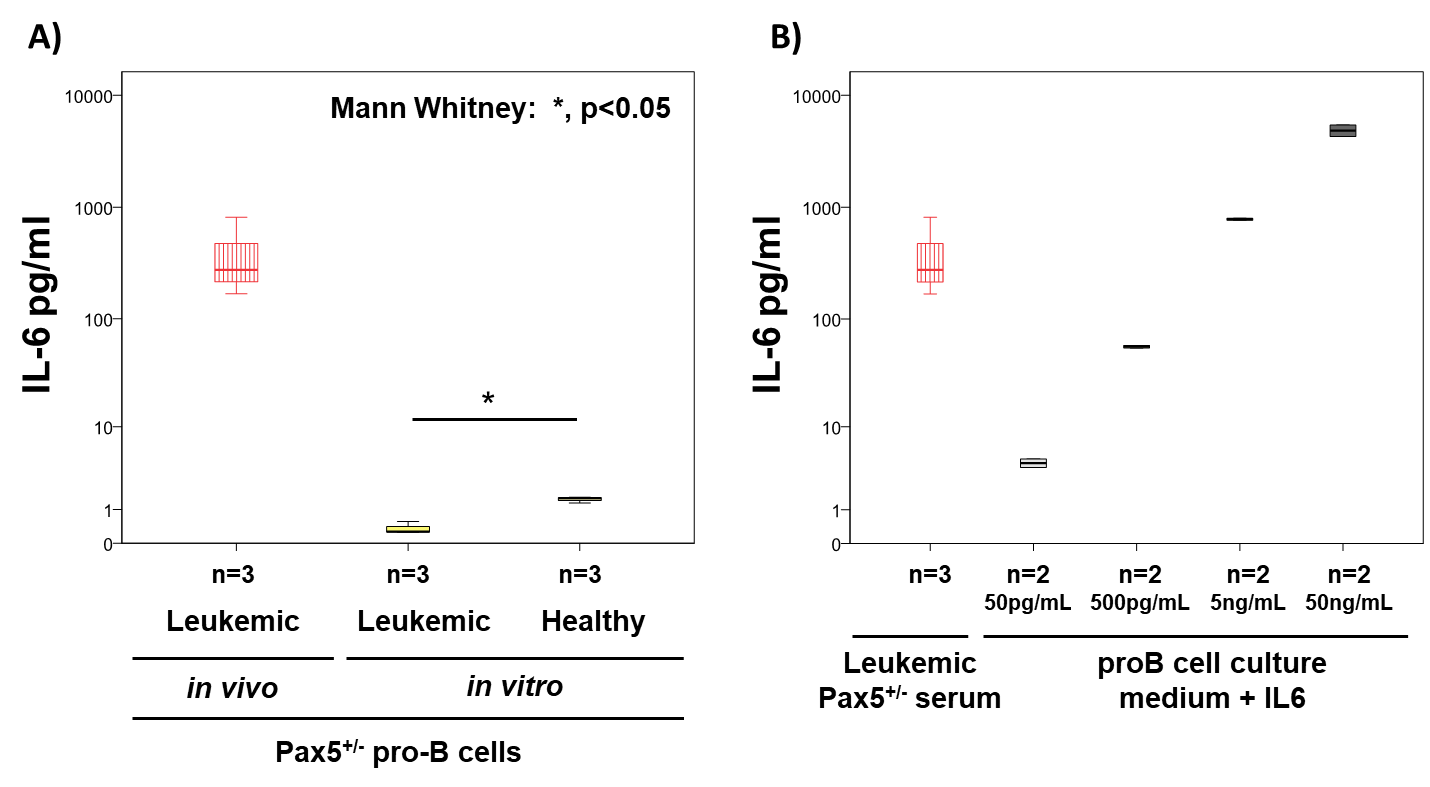
**

**Supplementary Figure 11. Quantification of IL6 levels within *in vitro* Pax5^+/-^ proB cells. A**) Supernatant from leukemic (n=3) and healthy (n=3) Pax5^+/-^ proB cells were grown *in vitro* were assessed to measure IL6 levels using the Cytometric Bead Array immunoassay system (CBA). Serum from leukemic Pax5+/- mice was used as a positive control (n=3). **B**) Dilutions of fixed amounts of IL6 into the proB cell culture medium were used as positive controls for the measurement of IL6 *in vitro*.

**
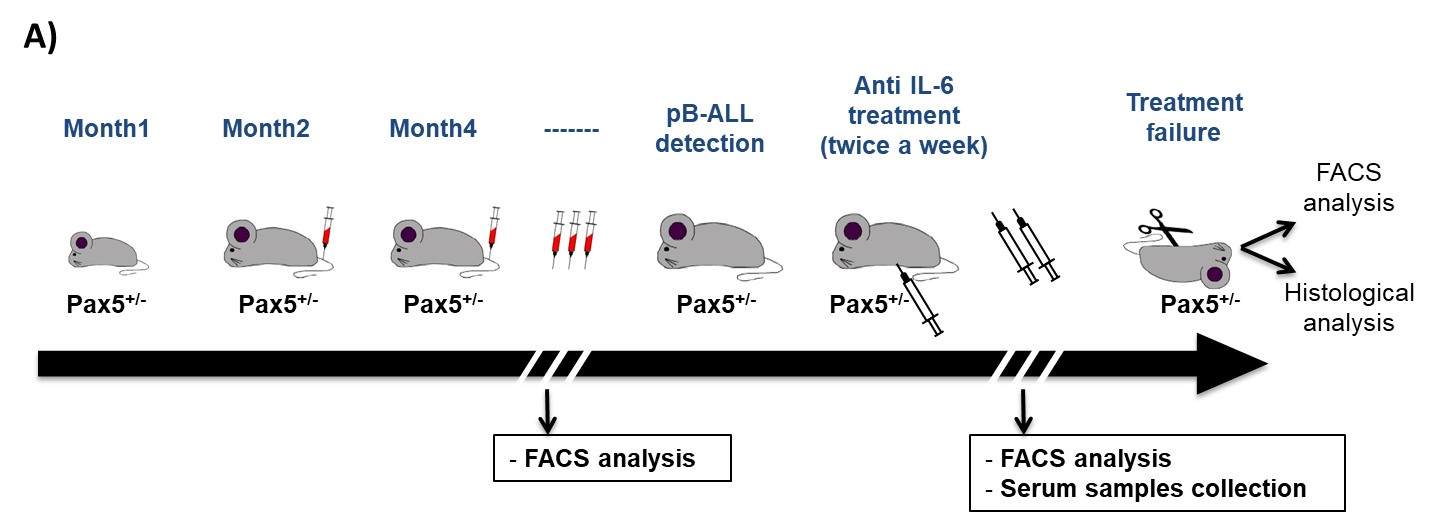
**

**
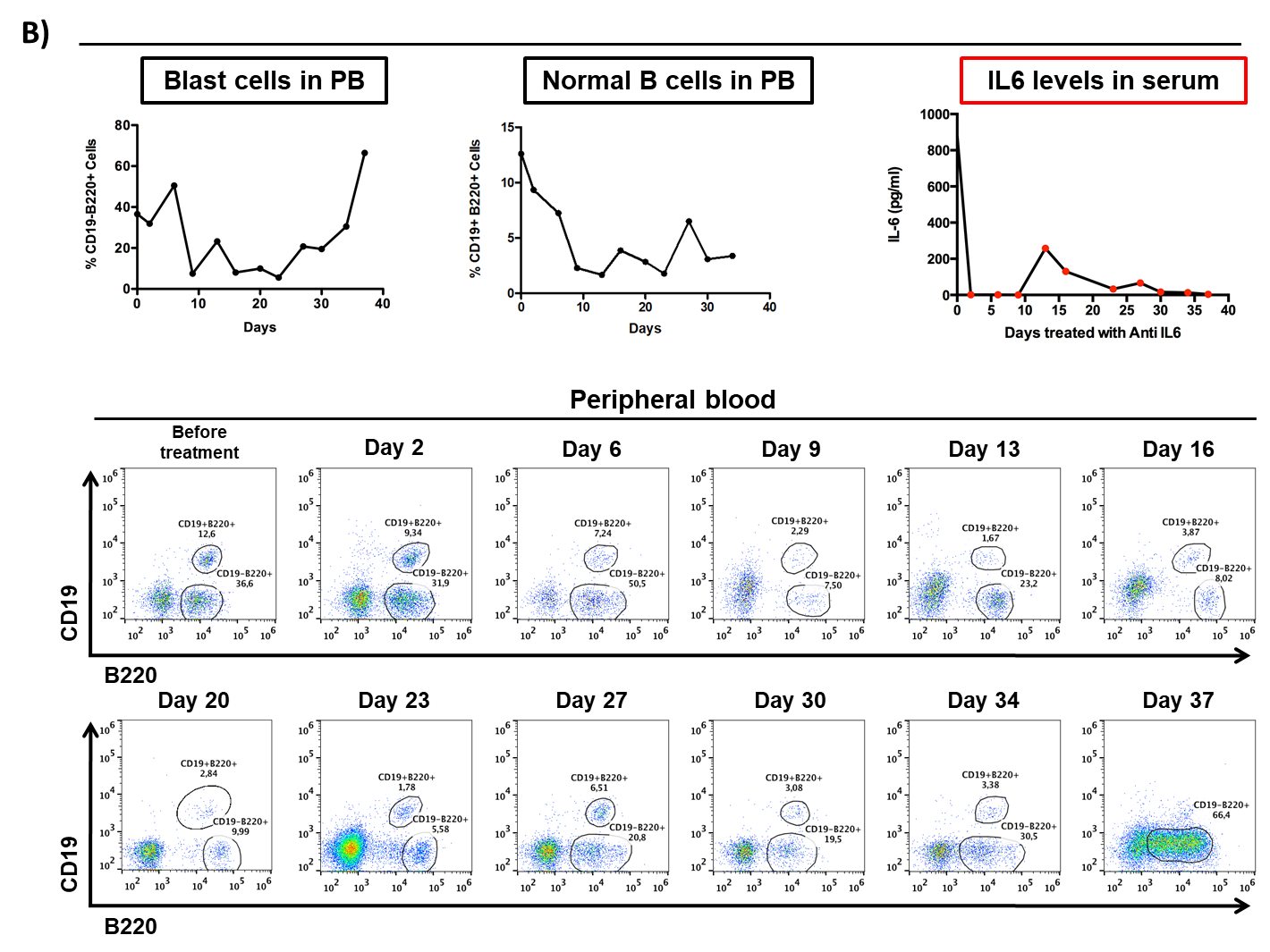
**

**
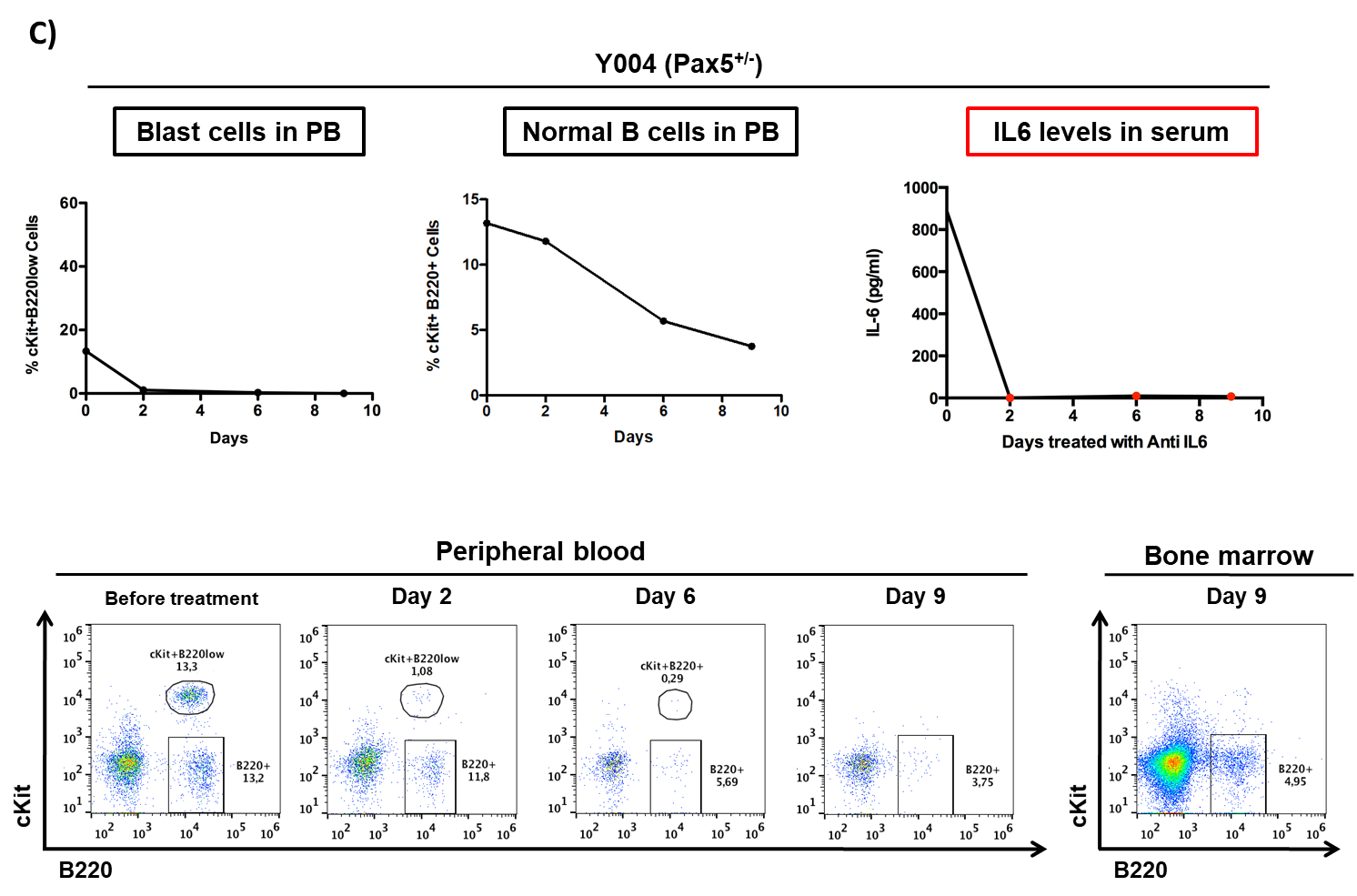
**

**
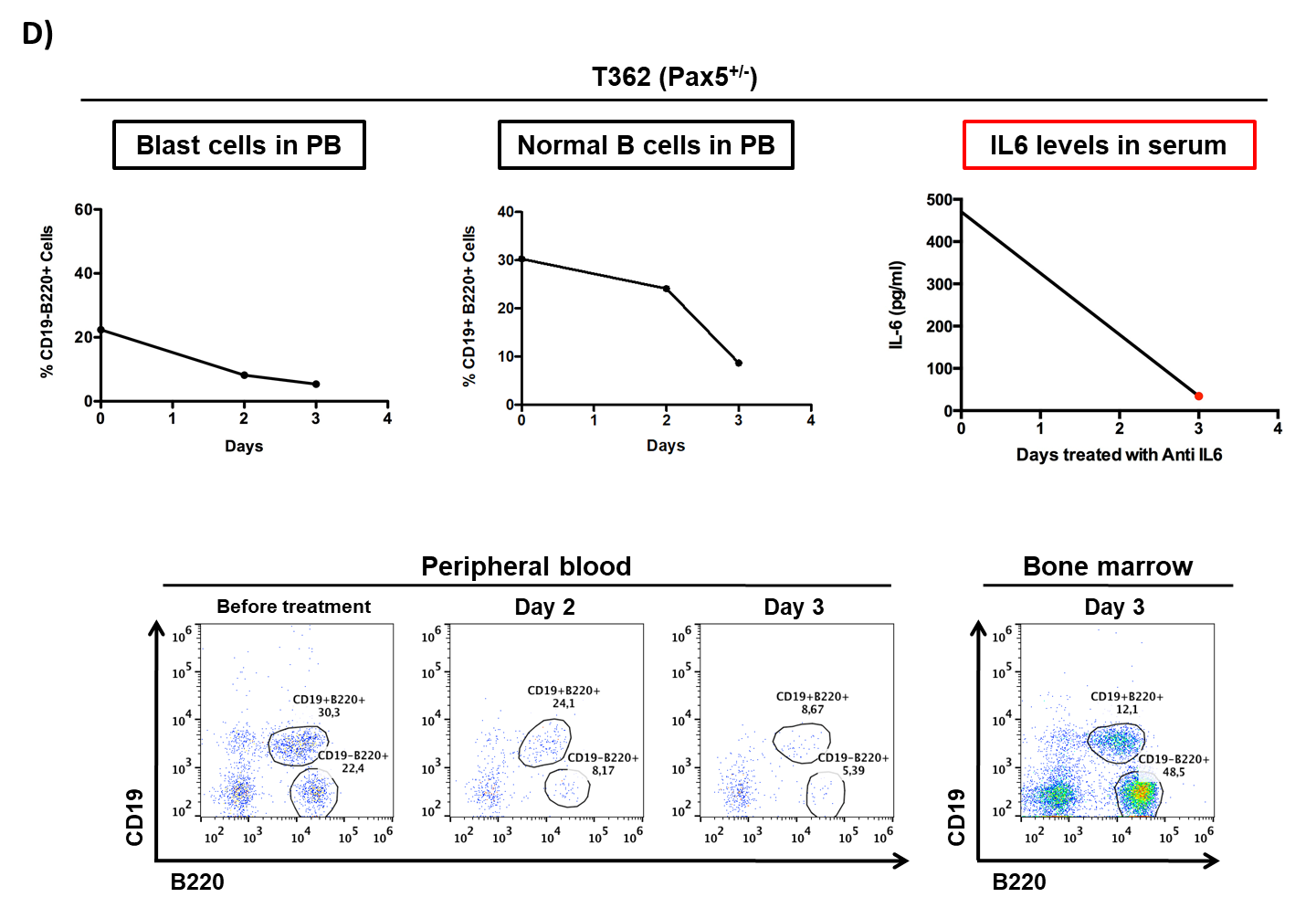
**

**
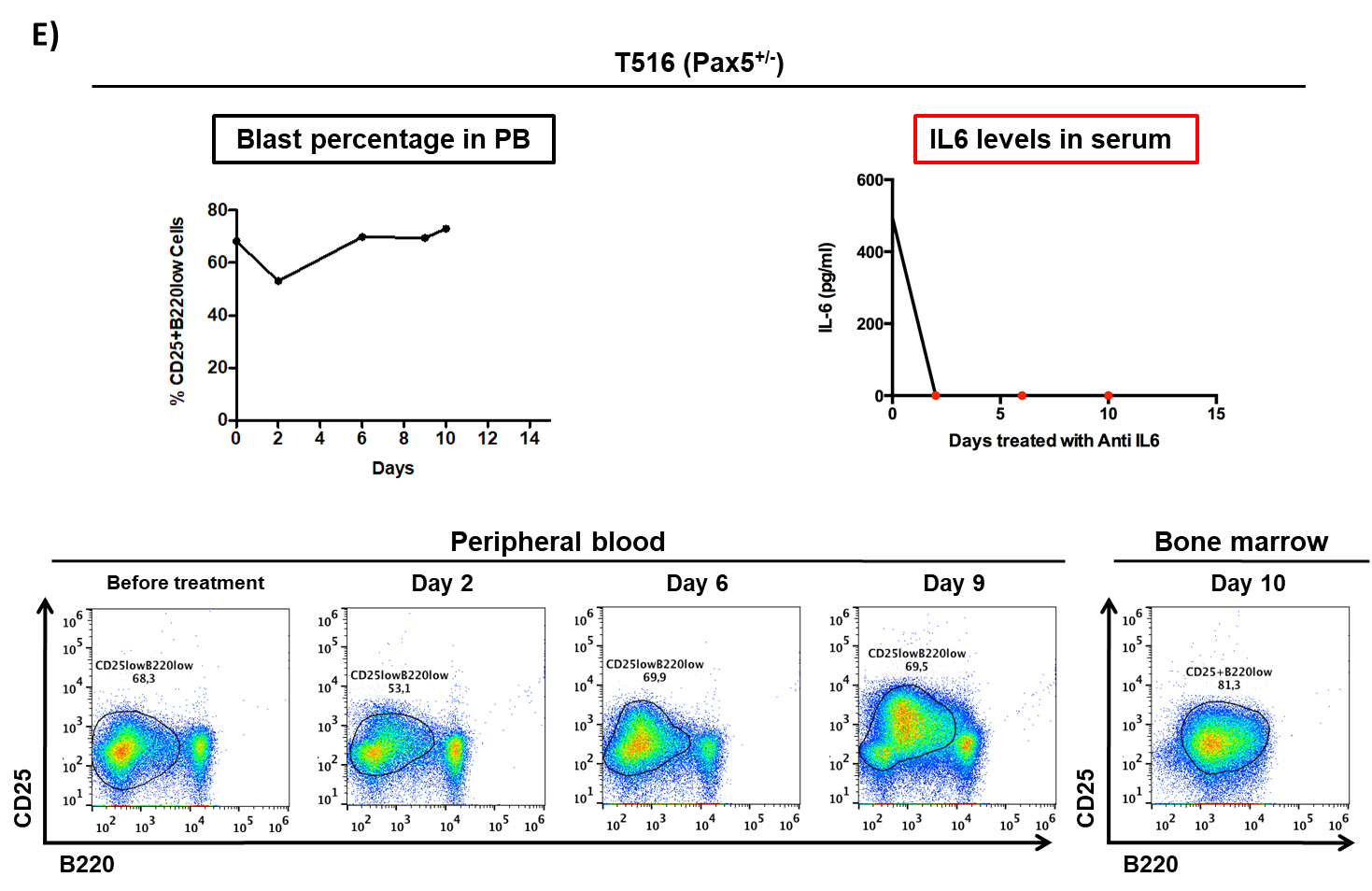
**

**
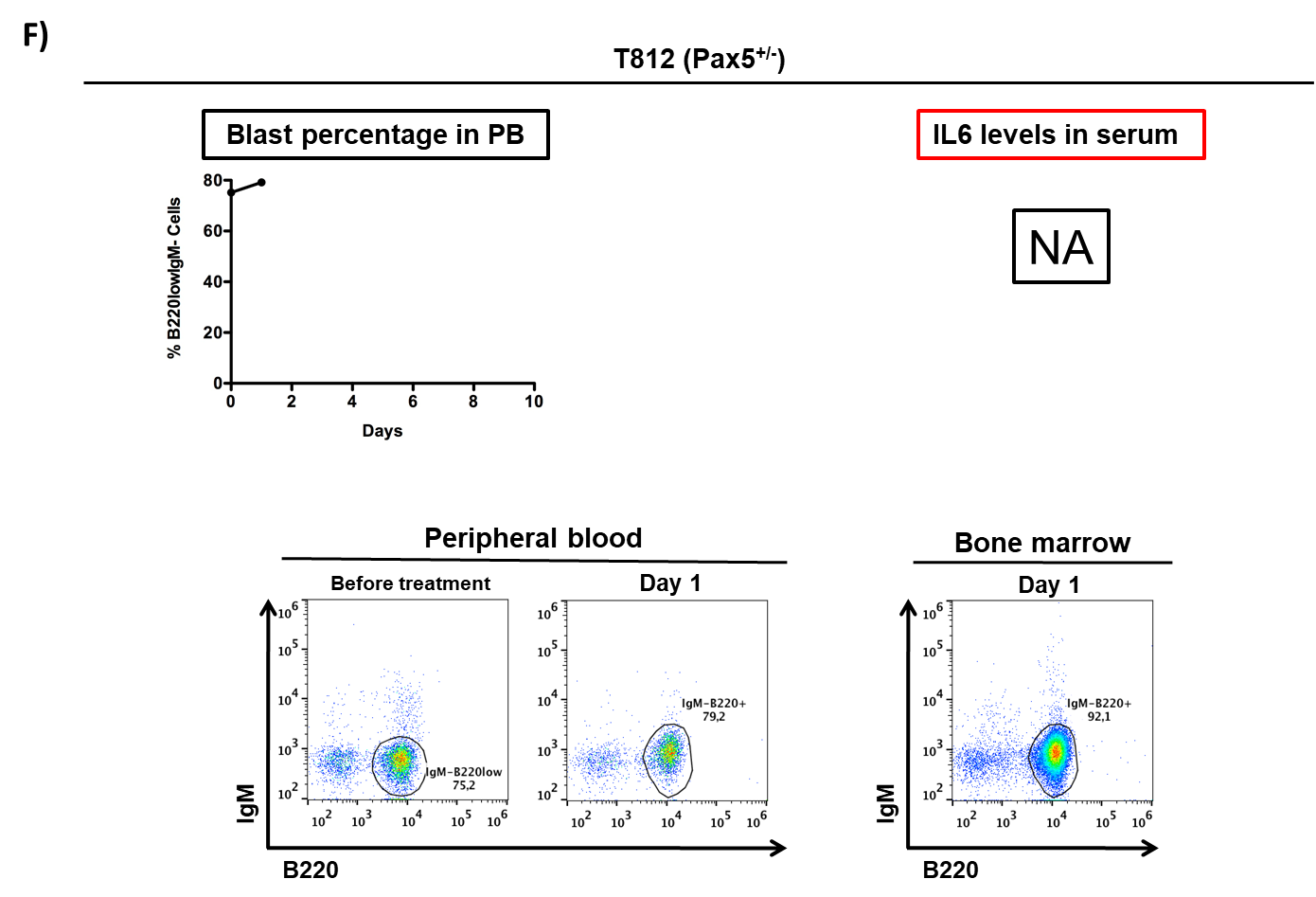
**

**
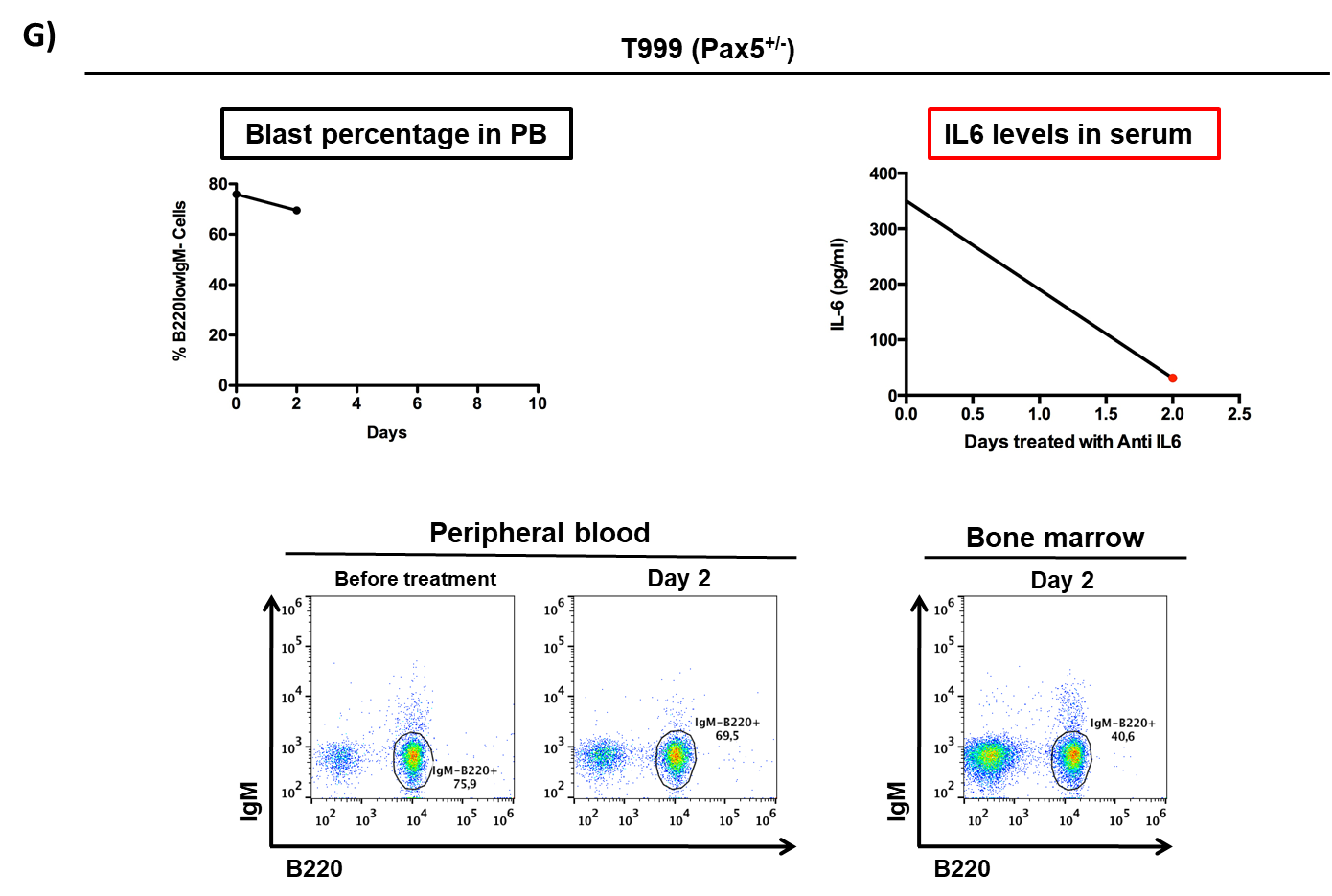
**

**Supplementary Figure 12. Anti-IL6 treatment into leukemic Pax5^+/-^ mice. A**) Experimental set-up of Anti-IL6 treatment into leukemic Pax5^+/-^ mice. Anti-IL6 antibody was given at 10 mg/kg twice a week once blast cells detection in PB by FACs analysis. Serum samples were collected before and during the anti-IL6 treatment. Anti-IL6 treatment into leukemic Pax5^+/-^ mice efficacy was assessed by monitoring blast cells, normal B cells and IL6 levels in PB by FACS analysis. Responder mice (to the treatment) are shown in panels **B-D** and non-responder mice are shown in panels **E-G**.

**
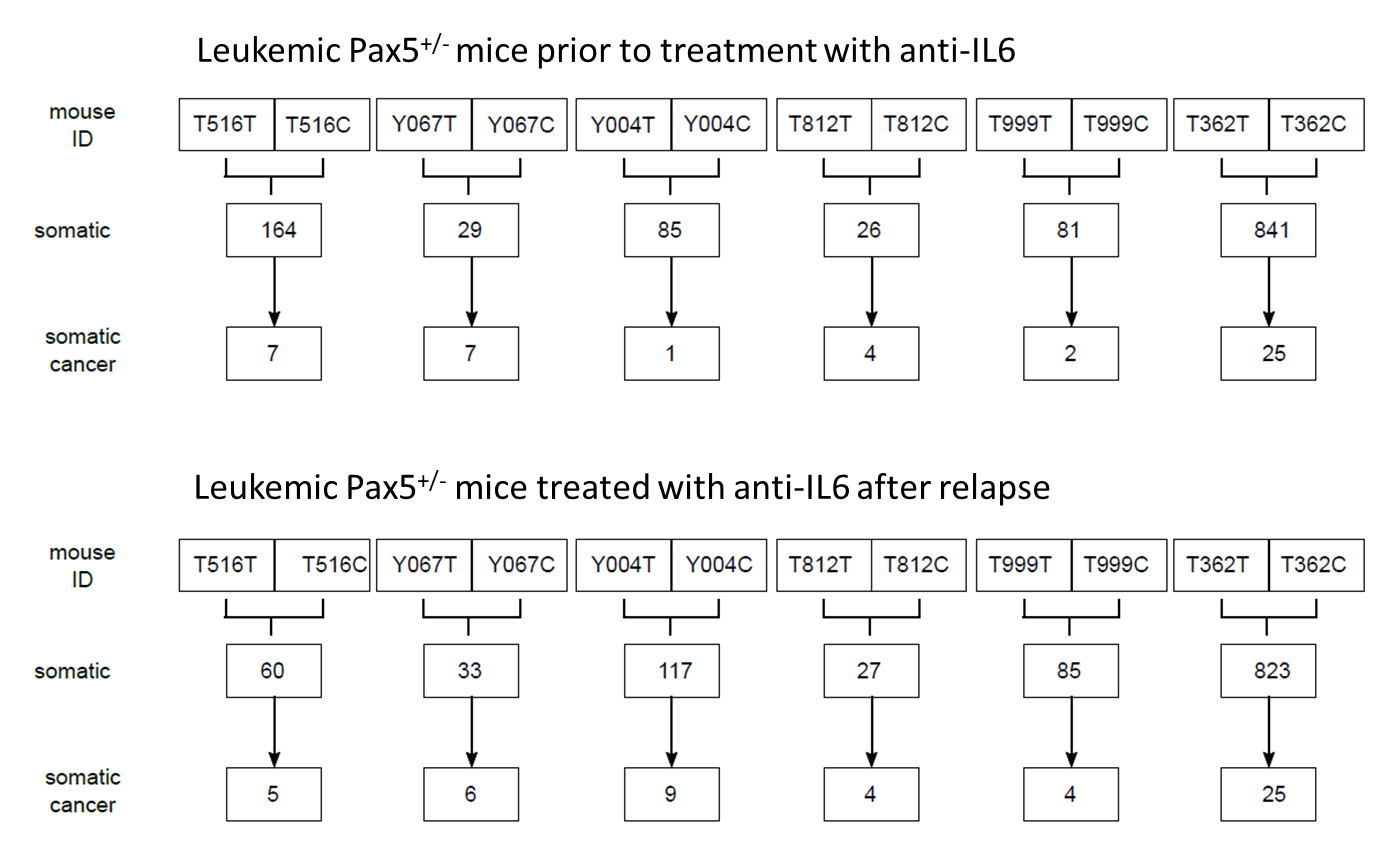
**

**Supplementary Figure 13. Exome sequencing in leukemic Pax5+/- mice before and after anti-IL6 treatment at the time of relapse.** Whole-exome sequencing analysis of tumor and control samples. Tumor cells prior to treatment were collected form peripheral blood (PB) and leukemic cells after relapse were collected from bone marrow (BM). Tumor-specific somatic mutations were determined by *mutect* and *varscan* analysis. The number of somatic cancer genes was calculated by using the cancer gene consensus list. The percentage of leukemic cells for each mouse were: T5167 PB: 30% and BM: 96%, Y067 PB: 36% and BM: 90%, Y004 PB: 13% and BM: 4%, T812 PB: 70% and BM: 94%, T999 PB: 75% and BM: 40% and T362 PB: 35% and BM: 48%.


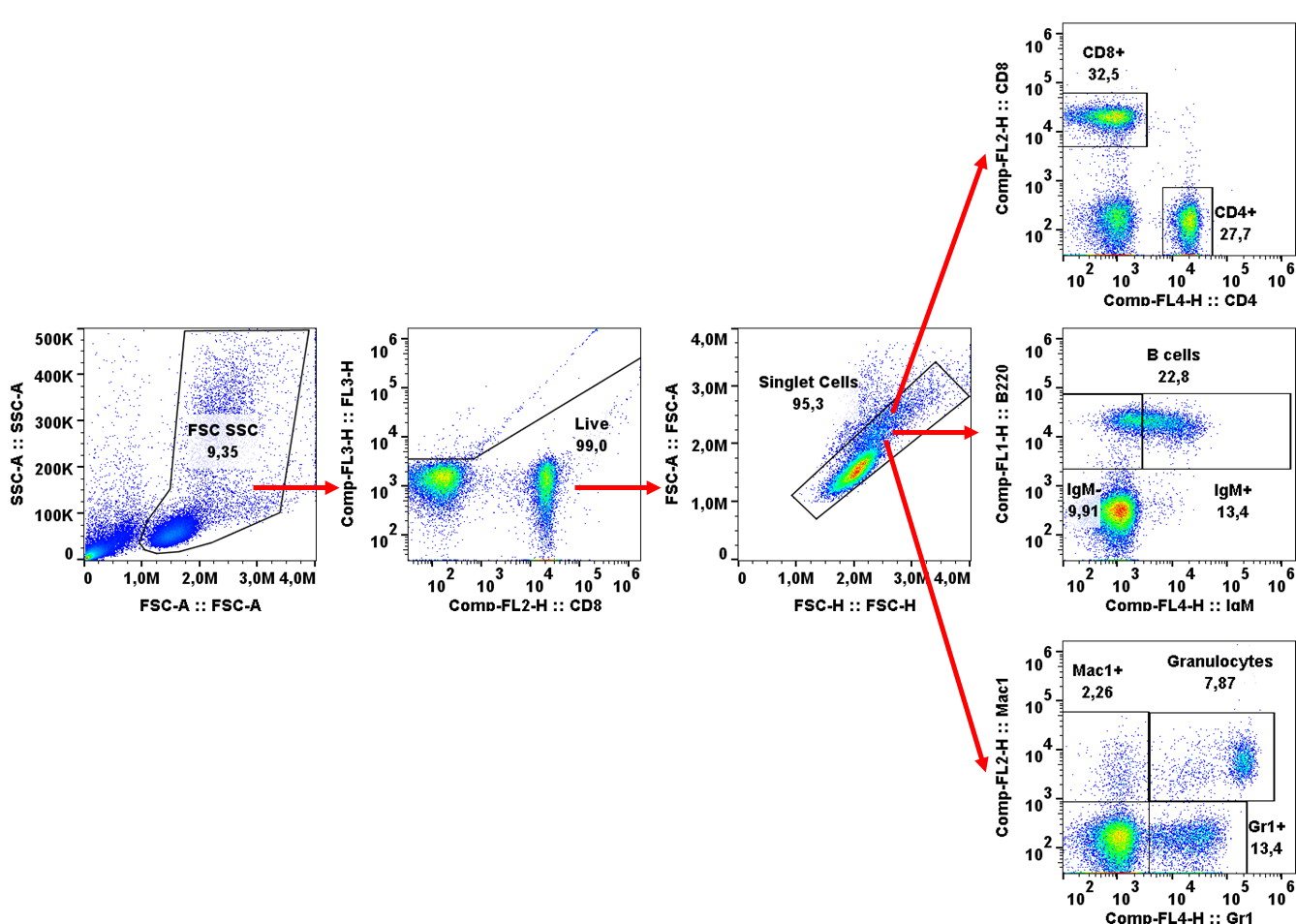


**Supplementary Figure 14. Gating strategy used in FACs analysis.** Figure exemplifying the gating strategy used in all cytometric analysis. For each analysis, a total of at least 50,000 viable cells (PI-; Propidium iodide negative cells) were assessed. Singlets were selected prior gating strategy that is specific for each population. It is shown as an example, peripheral blood cells stained with CD8-PE, CD4-APC, IgM-APC, B220-FITC, Mac1-PE and Gr1-APC. The same gating strategy has been used in all FACs analysis presented in Figure 5B-C, Supplementary Figure 5, Supplementary Figure 9-10 and Supplementary Figure 12.
