# Supplementary material for: Inhibition of inflammatory signaling in Pax5 mutant cells mitigates B-cell leukemogenesis
Source: Sci Rep. 2020 Nov 5;10:19189. doi: 10.1038/s41598-020-76206-y (PMC7644722; doi:10.1038/s41598-020-76206-y)
Supplement: Supplementary file 2 — Supplementary Information [file 41598_2020_76206_MOESM2_ESM.docx]

**Supplementary Table S1.** **Mouse serum samples description.** Genotype and IL6 serum levels of IL6^+/-^, IL6^-/-^, Pax5^+/-^ Non-Leukemic, IL6^+/-^ + Pax5^+/-^ Non-Leukemic, Pax5^+/-^ Leukemic, IL6^+/-^ + Pax5^+/-^ Leukemic, BCR/ABL^p190^ + Pax5^+/-^ Leukemic, ETV6-RUNX1 Leukemic and ETV6-RUNX1 + Pax5^+/-^ Leukemic mice and wild-type controls. All the mice have been housed in a conventional facility where the mice are exposed to common infections.

| CODE | GENDER | GENOTYPE | Age at time of determination | IL-2 levels (pg/ml) | IL-4 levels (pg/ml) | IL-6 levels (pg/ml) | IL-10 levels (pg/ml) | IL-17a levels (pg/ml) | IFNγ levels (pg/ml) | TNFα levels (pg/ml) |
| --- | --- | --- | --- | --- | --- | --- | --- | --- | --- | --- |
| W195 | MALE | WILD TYPE | 12.5 months | 0.46 | 0.00 | 0.29 | 0.00 | 0.43 | 0.00 | 3.01 |
| W542 | FEMALE | WILD TYPE | 10.9 months | 0.68 | 0.00 | 1.67 | 0.00 | 0.43 | 0.00 | 1.30 |
| W450 | FEMALE | WILD TYPE | 10.9 months | 0.57 | 1.57 | 2.04 | 0.00 | 2.81 | 0.55 | 5.43 |
| S949 | FEMALE | WILD TYPE | 13.3 months | 0.68 | 0.00 | 10.29 | 0.00 | 1.83 | 0.25 | 3.29 |
| C153 | FEMALE | WILD TYPE | 8 months | 0.61 | 0.00 | 0.57 | 0.00 | 0.84 | 0.12 | 0.00 |
| C183 | FEMALE | WILD TYPE | 8 months | 0.75 | 0.35 | 1.96 | 0.00 | 1.65 | 0.19 | 7.96 |
| C146 | MALE | WILD TYPE | 8 months | 0.43 | 2.15 | 2.26 | 0.00 | 3.53 | 1.36 | 6.64 |
| WT-1 | FEMALE | WILD TYPE | 4 months | 0.71 | 0.00 | 1.24 | 0.00 | 0.00 | 0.00 | 2.47 |
| WT-2 | FEMALE | WILD TYPE | 4 months | 0.68 | 0.00 | 2.49 | 0.00 | 0.00 | 0.00 | 1.56 |
| WT-3 | FEMALE | WILD TYPE | 4 months | 0.23 | 0.00 | 0.73 | 0.00 | 0.00 | 0.25 | 1.81 |
| WT-4 | FEMALE | WILD TYPE | 4 months | 0.23 | 0.00 | 1.53 | 0.00 | 0.00 | 0.00 | 2.34 |
| WT-5 | FEMALE | WILD TYPE | 4 months | 0.08 | 0.00 | 0.73 | 0.00 | 0.02 | 0.12 | 1.56 |
| WT-6 | FEMALE | WILD TYPE | 4 months | 0.43 | 0.00 | 3.47 | 0.00 | 0.17 | 0.00 | 2.07 |
| WT-7 | FEMALE | WILD TYPE | 4 months | 0.89 | 0.80 | 3.30 | 0.00 | 0.00 | 0.22 | 1.56 |
| WT-6 | MALE | WILD TYPE | 22 months | 0.93 | 2.07 | 5.16 | 2.08 | 3.76 | 1.05 | 18.59 |
| WT-20 | MALE | WILD TYPE | 22.3 months | 0.86 | 1.57 | 3.64 | 2.43 | 4.08 | 1.20 | 1.56 |
| W195 | MALE | WILD TYPE | 12.5 months | 0.00 | 0.00 | 0.00 | 0.00 | 1.94 | 0.00 | 0.15 |
| W542 | FEMALE | WILD TYPE | 10.9 months | 0.00 | 0.00 | 0.00 | 0.00 | 0.00 | 0.00 | 0.00 |
| W450 | FEMALE | WILD TYPE | 10.9 months | 0.00 | 8.27 | 10.07 | 15.35 | 12.58 | 2.62 | 0.00 |
| S949 | FEMALE | WILD TYPE | 8.8 months | 0.00 | 0.14 | 4.23 | 3.31 | 3.51 | 0.00 | 0.00 |
| C153 | FEMALE | WILD TYPE | 8 months | 0.00 | 0.00 | 0.00 | 0.00 | 0.05 | 0.00 | 0.00 |
| C183 | FEMALE | WILD TYPE | 8 months | 0.00 | 0.00 | 0.54 | 4.34 | 2.09 | 0.00 | 5.24 |
| C184 | FEMALE | WILD TYPE | 23 months | 0.00 | 0.00 | 0.00 | 0.00 | 0.13 | 0.00 | 0.00 |
| C146 | MALE | WILD TYPE | 24.3 months | 0.00 | 16.18 | 27.04 | 22.23 | 22.90 | 10.10 | 64.54 |
| R022 | MALE | WILD TYPE | 5.6 months | 0.00 | 0.00 | 0.00 | 0.00 | 0.00 | 0.00 | 0.00 |
| R104 | FEMALE | WILD TYPE | 5.3 months | 0.00 | 0.00 | 0.00 | 0.00 | 0.00 | 0.00 | 0.00 |
| R105 | FEMALE | WILD TYPE | 5.3 months | 0.00 | 0.00 | 0.00 | 0.00 | 0.00 | 0.76 | 1.19 |
| R106 | FEMALE | WILD TYPE | 5.3 months | 0.00 | 0.00 | 0.00 | 0.00 | 0.00 | 0.79 | 1.00 |
| R319 | MALE | WILD TYPE | 5.7 months | 0.00 | 0.00 | 0.00 | 0.00 | 0.00 | 0.00 | 0.00 |
| R320 | MALE | WILD TYPE | 5.7 months | 0.00 | 0.00 | 0.00 | 0.00 | 0.00 | 0.25 | 0.00 |
| R323 | MALE | WILD TYPE | 5.1 months | 0.00 | 0.00 | 15.36 | 0.00 | 0.00 | 0.00 | 1.89 |
| R328 | FEMALE | WILD TYPE | 5.1 months | 0.00 | 0.00 | 0.00 | 0.00 | 0.00 | 0.00 | 0.00 |
| R022 | MALE | WILD TYPE | 11.7 months | 0.00 | 0.00 | 1.91 | 0.00 | 0.00 | 0.00 | 1.99 |
| R104 | FEMALE | WILD TYPE | 11.5 months | 0.00 | 0.00 | 15.36 | 0.00 | 0.00 | 0.00 | 1.59 |
| R105 | FEMALE | WILD TYPE | 11.5 months | 0.00 | 0.80 | 0.00 | 0.00 | 0.37 | 0.25 | 1.00 |
| R106 | FEMALE | WILD TYPE | 11.5 months | 0.61 | 1.45 | 0.83 | 0.00 | 0.30 | 0.34 | 0.69 |
| R319 | MALE | WILD TYPE | 10.9 months | 0.81 | 0.90 | 4.49 | 0.00 | 2.44 | 0.00 | 0.00 |
| R320 | MALE | WILD TYPE | 10.9 months | 0.30 | 0.70 | 0.43 | 0.00 | 0.00 | 0.00 | 0.00 |
| R323 | MALE | WILD TYPE | 10.9 months | 0.38 | 0.00 | 2.61 | 104.82 | 0.00 | 0.00 | 3.66 |
| R328 | FEMALE | WILD TYPE | 10.9 months | 0.19 | 0.00 | 0.00 | 0.00 | 0.00 | 0.00 | 0.00 |
| R022 | MALE | WILD TYPE | 18.07 months | 0.65 | 1.20 | 10.65 | 0.00 | 0.00 | 0.00 | 0.00 |
| R104 | FEMALE | WILD TYPE | 17.9 months | 0.69 | 4.01 | 88.47 | 23.50 | 5.41 | 2.93 | 6.79 |
| R105 | FEMALE | WILD TYPE | 17.9 months | 0.15 | 1.30 | 17.90 | 0.00 | 4.06 | 0.90 | 3.66 |
| R106 | FEMALE | WILD TYPE | 17.9 months | 14.46 | 2.89 | 16.48 | 22.31 | 13.20 | 308.05 | 1.49 |
| R319 | MALE | WILD TYPE | 17.9 months | 0.00 | 0.00 | 58.00 | 0.00 | 5.64 | 0.16 | 3.42 |
| R320 | MALE | WILD TYPE | 17.9 months | 0.65 | 1.84 | 56.66 | 0.00 | 4.87 | 0.00 | 2.85 |
| R323 | MALE | WILD TYPE | 17.9 months | 0.42 | 1.50 | 22.19 | 0.00 | 0.22 | 0.12 | 0.00 |
| R328 | FEMALE | WILD TYPE | 17.8 months | 0.89 | 0.70 | 133.04 | 0.00 | 0.00 | 0.00 | 5.40 |
| WT (POS. 1) | FEMALE | WILD TYPE | 11 months | 0.00 | 0.70 | 0.51 | 1.01 | 0.90 | 0.50 | 2.62 |
| WT (POS. 8) | FEMALE | WILD TYPE | 12 months | 0.00 | 0.00 | 0.39 | 0.00 | 0.29 | 1.79 | 5.33 |
| WT (POS. 9) | FEMALE | WILD TYPE | 11 months | 0.00 | 0.00 | 12.40 | 1.79 | 2.01 | 0.29 | 3.44 |
| WT (POS. 10) | FEMALE | WILD TYPE | 11 months | 0.00 | 0.56 | 4.45 | 1.45 | 0.01 | 0.36 | 4.37 |
| WT-19 | FEMALE | WILD TYPE | 5 months | 0.00 | 0.54 | 0.00 | 0.50 | 0.00 | 0.00 | 2.17 |
| WT-21 | FEMALE | WILD TYPE | 5 months | 0.00 | 0.17 | 0.63 | 0.50 | 0.06 | 0.00 | 2.67 |
| WT-22 | FEMALE | WILD TYPE | 5 months | 0.00 | 0.00 | 4.21 | 0.00 | 0.94 | 0.28 | 2.43 |
| R020 | MALE | WILD TYPE | 11 months | 0.00 | 0.68 | 28.23 | 5.94 | 2.78 | 0.49 | 10.49 |
| WT-21 | FEMALE | WILD TYPE | 11 months | 0.00 | 0.00 | 1.22 | 0.35 | 0.58 | 0.00 | 3.25 |
| WT-22 | FEMALE | WILD TYPE | 11 months | 0.00 | 0.00 | 0.00 | 0.22 | 0.69 | 0.60 | 3.54 |
| WT-19 | FEMALE | WILD TYPE | 17 months | 0.00 | 0.87 | 39.11 | 5.52 | 6.32 | 1.88 | 40.72 |
| WT-21 | FEMALE | WILD TYPE | 17 months | 1.47 | 0.00 | 11.74 | 1.73 | 2.06 | 0.00 | 3.91 |
| WT-22 | FEMALE | WILD TYPE | 17 months | 0.00 | 0.62 | 3.51 | 4.41 | 5.26 | 1.88 | 5.58 |
| L575 | FEMALE | IL6+/- Non-Leukemic | 5 months | 0.00 | 0.00 | 0.00 | 0.00 | 0.00 | 0.00 | 5.66 |
| L575 | FEMALE | IL6+/- Non-Leukemic | 11 months | 0.00 | 0.00 | 0.08 | 2.08 | 3.14 | 0.00 | 18.92 |
| L575 | FEMALE | IL6+/- Non-Leukemic | 17 months | 0.00 | 0.00 | 0.40 | 0.00 | 0.86 | 0.00 | 4.65 |
| U370 | FEMALE | IL6+/- Non-Leukemic | 11 months | 0.00 | 0.00 | 0.00 | 0.45 | 0.00 | 0.22 | 0.85 |
| U372 | FEMALE | IL6+/- Non-Leukemic | 5 months | 0.00 | 0.00 | 0.00 | 0.82 | 0.00 | 1.03 | 5.41 |
| U372 | FEMALE | IL6+/- Non-Leukemic | 11 months | 0.00 | 0.01 | 0.00 | 1.28 | 0.00 | 7.11 | 5.33 |
| U372 | FEMALE | IL6+/- Non-Leukemic | 17 months | 0.00 | 0.14 | 0.11 | 1.92 | 0.65 | 1.50 | 3.42 |
| U578 | FEMALE | IL6+/- Non-Leukemic | 5 months | 0.00 | 0.00 | 0.00 | 5.83 | 0.06 | 0.64 | 3.91 |
| U578 | FEMALE | IL6+/- Non-Leukemic | 11 months | 0.00 | 0.00 | 0.00 | 3.11 | 0.02 | 0.97 | 3.03 |
| U578 | FEMALE | IL6+/- Non-Leukemic | 17 months | 0.00 | 0.00 | 0.22 | 1.11 | 0.49 | 0.47 | 2.21 |
| L164 | FEMALE | IL6-/- Non-Leukemic | 5 months | 0.00 | 0.00 | 0.00 | 0.00 | 0.32 | 1.15 | 2.93 |
| L164 | FEMALE | IL6-/- Non-Leukemic | 11 months | 0.00 | 0.00 | 0.00 | 0.00 | 0.01 | 0.00 | 3.37 |
| L164 | FEMALE | IL6-/- Non-Leukemic | 17 months | 0.00 | 0.10 | 0.00 | 1.82 | 0.10 | 0.66 | 2.77 |
| U417 | MALE | IL6-/- Non-Leukemic | 5 months | 0.00 | 0.00 | 0.00 | 0.00 | 0.01 | 0.00 | 1.87 |
| U417 | MALE | IL6-/- Non-Leukemic | 11 months | 0.01 | 0.20 | 0.00 | 1.68 | 0.60 | 0.00 | 3.91 |
| U417 | MALE | IL6-/- Non-Leukemic | 17 months | 0.00 | 0.00 | 0.00 | 1.73 | 0.00 | 0.00 | 2.17 |
| U418 | MALE | IL6-/- Non-Leukemic | 5 months | 0.00 | 0.00 | 0.00 | 0.50 | 1.87 | 0.04 | 1.87 |
| U418 | MALE | IL6-/- Non-Leukemic | 11 months | 0.00 | 0.00 | 0.00 | 4.58 | 0.06 | 0.00 | 2.62 |
| U418 | MALE | IL6-/- Non-Leukemic | 17 months | 0.00 | 0.38 | 0.00 | 2.42 | 0.24 | 0.00 | 3.20 |
| Y554 | MALE | IL6-/- Non-Leukemic | 11 months | 0.00 | 0.00 | 0.00 | 1.01 | 0.11 | 0.05 | 5.13 |
| R017 | FEMALE | PAX5+/- Non-Leukemic 5 months | 5.6 months | 0.50 | 0.80 | 1.73 | 0.00 | 2.22 | 1.08 | 4.97 |
| R018 | FEMALE | PAX5+/- Non-Leukemic 5 months | 5.6 months | 0.00 | 0.00 | 0.00 | 0.00 | 0.00 | 0.01 | 0.00 |
| R019 | MALE | PAX5+/- Non-Leukemic 5 months | 5.6 months | 0.07 | 0.00 | 1.39 | 0.00 | 0.00 | 0.00 | 3.91 |
| R020 | MALE | PAX5+/- Non-Leukemic 5 months | 5.6 months | 0.00 | 0.00 | 0.00 | 0.00 | 0.00 | 0.00 | 0.00 |
| R021 | FEMALE | PAX5+/- Non-Leukemic 5 months | 5.6 months | 0.00 | 0.00 | 0.00 | 0.00 | 1.01 | 0.41 | 0.00 |
| R103 | MALE | PAX5+/- Non-Leukemic 5 months | 5.3 months | 0.00 | 0.00 | 91.49 | 0.00 | 25.66 | 0.68 | 1.19 |
| R322 | MALE | PAX5+/- Non-Leukemic 5 months | 5.1 months | 0.00 | 0.00 | 1.08 | 0.00 | 0.82 | 0.00 | 1.29 |
| R324 | FEMALE | PAX5+/- Non-Leukemic 5 months | 5.1 months | 0.00 | 0.00 | 0.00 | 0.00 | 3.16 | 1.08 | 0.00 |
| R327 | FEMALE | PAX5+/- Non-Leukemic 5 months | 5.1 months | 0.00 | 0.00 | 0.00 | 0.00 | 0.00 | 0.00 | 0.00 |
| R329 | FEMALE | PAX5+/- Non-Leukemic 5 months | 5.1 months | 0.00 | 0.00 | 0.98 | 0.00 | 0.00 | 0.00 | 1.89 |
| R330 | FEMALE | PAX5+/- Non-Leukemic 5 months | 5.1 months | 0.00 | 0.00 | 0.00 | 0.00 | 0.00 | 0.00 | 1.19 |
| R331 | FEMALE | PAX5+/- Non-Leukemic 5 months | 5.1 months | 0.57 | 0.00 | 0.38 | 0.00 | 0.95 | 0.53 | 3.30 |
| R021 | FEMALE | PAX5+/- Non-Leukemic 5 months | 5 months | 0.00 | 0.00 | 0.00 | 0.12 | 0.35 | 0.00 | 4.87 |
| R016 | MALE | PAX5+/- Non-Leukemic 5 months | 5 months | 0.00 | 0.43 | 0.78 | 2.60 | 0.76 | 0.33 | 5.33 |
| R020 | MALE | PAX5+/- Non-Leukemic 5 months | 5 months | 0.00 | 0.00 | 0.40 | 0.56 | 0.24 | 0.00 | 3.20 |
| R017 | FEMALE | PAX5+/- Non-Leukemic 11 months | 11.8 months | 0.00 | 1.50 | 2.41 | 0.00 | 1.26 | 1.21 | 2.52 |
| R018 | FEMALE | PAX5+/- Non-Leukemic 11 months | 11.8 months | 0.00 | 1.25 | 0.00 | 0.00 | 0.00 | 0.74 | 0.00 |
| R019 | MALE | PAX5+/- Non-Leukemic 11 months | 11.8 months | 0.00 | 0.00 | 146.66 | 0.00 | 17.65 | 0.12 | 11.64 |
| R020 | MALE | PAX5+/- Non-Leukemic 11 months | 11.8 months | 0.00 | 0.00 | 1.08 | 0.00 | 0.00 | 0.00 | 0.00 |
| R021 | FEMALE | PAX5+/- Non-Leukemic 11 months | 11.8 months | 0.00 | 0.00 | 0.33 | 0.00 | 1.07 | 0.29 | 0.00 |
| R103 | MALE | PAX5+/- Non-Leukemic 11 months | 11.5 months | 0.00 | 0.00 | 3.17 | 0.00 | 9.50 | 0.03 | 7.81 |
| R322 | MALE | PAX5+/- Non-Leukemic 11 months | 10.9 months | 0.15 | 1.05 | 3.03 | 0.00 | 27.12 | 0.08 | 0.00 |
| R327 | FEMALE | PAX5+/- Non-Leukemic 11 months | 10.9 months | 0.26 | 0.26 | 0.93 | 0.00 | 0.00 | 0.00 | 3.42 |
| R329 | FEMALE | PAX5+/- Non-Leukemic 11 months | 10.9 months | 0.00 | 0.00 | 0.00 | 0.00 | 0.00 | 0.00 | 0.00 |
| R330 | FEMALE | PAX5+/- Non-Leukemic 11 months | 10.9 months | 0.00 | 0.00 | 0.16 | 0.00 | 0.00 | 0.00 | 5.69 |
| R331 | FEMALE | PAX5+/- Non-Leukemic 11 months | 10.9 months | 0.00 | 0.00 | 0.00 | 0.00 | 0.00 | 0.00 | 2.52 |
| R021 | FEMALE | PAX5+/- Non-Leukemic 11 months | 11 months | 0.00 | 0.00 | 0.82 | 3.32 | 1.25 | 0.00 | 5.26 |
| R016 | MALE | PAX5+/- Non-Leukemic 11 months | 11 months | 0.00 | 1.58 | 4.71 | 13.50 | 4.62 | 3.95 | 11.47 |
| R020 | MALE | PAX5+/- Non-Leukemic 11 months | 11 months | 0.00 | 0.68 | 28.23 | 5.94 | 2.78 | 0.49 | 10.49 |
| R018 | FEMALE | PAX5+/- Non-Leukemic 18 months | 18.1 months | 0.38 | 1.84 | 3.32 | 0.00 | 1.14 | 0.63 | 0.00 |
| R019 | MALE | PAX5+/- Non-Leukemic 18 months | 18.1 months | 1.01 | 1.05 | 197.84 | 0.00 | 6.48 | 1.08 | 13.55 |
| R020 | MALE | PAX5+/- Non-Leukemic 18 months | 18.1 months | 0.61 | 0.34 | 4.86 | 0.00 | 0.00 | 0.00 | 1.00 |
| R021 | FEMALE | PAX5+/- Non-Leukemic 18 months | 18.1 months | 0.26 | 2.51 | 3.25 | 0.00 | 3.25 | 1.95 | 0.59 |
| R103 | MALE | PAX5+/- Non-Leukemic 18 months | 17.9 months | 1.86 | 1.84 | 7.88 | 0.00 | 2.60 | 1.30 | 0.00 |
| R322 | MALE | PAX5+/- Non-Leukemic 18 months | 17.8 months | 5.12 | 4.01 | 124.63 | 0.00 | 2.67 | 1.05 | 4.70 |
| R327 | FEMALE | PAX5+/- Non-Leukemic 18 months | 17.8 months | 0.50 | 1.40 | 3.17 | 0.00 | 0.00 | 0.10 | 1.89 |
| R329 | FEMALE | PAX5+/- Non-Leukemic 18 months | 17.8 months | 0.00 | 3.23 | 58.00 | 0.00 | 2.07 | 0.55 | 2.41 |
| R331 | FEMALE | PAX5+/- Non-Leukemic 18 months | 17.8 months | 0.69 | 8.55 | 108.07 | 58.26 | 9.34 | 3.97 | 9.11 |
| R021 | FEMALE | PAX5+/- Non-Leukemic 18 months | 17 months | 0.00 | 0.00 | 1.01 | 0.05 | 0.38 | 0.97 | 2.25 |
| R016 | MALE | PAX5+/- Non-Leukemic 18 months | 17 months | 0.00 | 4.31 | 67.82 | 40.81 | 11.65 | 7.51 | 13.67 |
| R020 | MALE | PAX5+/- Non-Leukemic 18 months | 17 months | 0.00 | 0.00 | 3.51 | 1.68 | 0.41 | 0.00 | 3.66 |
| R022 | MALE | WILD TYPE 5 months | 5.6 months | 0.00 | 0.00 | 0.00 | 0.00 | 0.00 | 0.00 | 0.00 |
| R104 | FEMALE | WILD TYPE 5 months | 5.3 months | 0.00 | 0.00 | 0.00 | 0.00 | 0.00 | 0.00 | 0.00 |
| R105 | FEMALE | WILD TYPE 5 months | 5.3 months | 0.00 | 0.00 | 0.00 | 0.00 | 0.00 | 0.76 | 1.19 |
| R106 | FEMALE | WILD TYPE 5 months | 5.3 months | 0.00 | 0.00 | 0.00 | 0.00 | 0.00 | 0.79 | 1.00 |
| R319 | MALE | WILD TYPE 5 months | 5.1 months | 0.00 | 0.00 | 0.00 | 0.00 | 0.00 | 0.00 | 0.00 |
| R320 | MALE | WILD TYPE 5 months | 5.1 months | 0.00 | 0.00 | 0.00 | 0.00 | 0.00 | 0.25 | 0.00 |
| R323 | MALE | WILD TYPE 5 months | 5.1 months | 0.00 | 0.00 | 15.36 | 0.00 | 0.00 | 0.00 | 1.89 |
| R328 | FEMALE | WILD TYPE 5 months | 5.1 months | 0.00 | 0.00 | 0.00 | 0.00 | 0.00 | 0.00 | 0.00 |
| WT-19 | FEMALE | WILD TYPE 5 months | 5 months | 0.00 | 0.54 | 0.00 | 0.50 | 0.00 | 0.00 | 2.17 |
| WT-21 | FEMALE | WILD TYPE 5 months | 5 months | 0.00 | 0.17 | 0.63 | 0.50 | 0.06 | 0.00 | 2.67 |
| WT-22 | FEMALE | WILD TYPE 5 months | 5 months | 0.00 | 0.00 | 4.21 | 0.00 | 0.94 | 0.28 | 2.43 |
| R022 | MALE | WILD TYPE 11 months | 11.8 months | 0.00 | 0.00 | 1.91 | 0.00 | 0.00 | 0.00 | 1.99 |
| R104 | FEMALE | WILD TYPE 11 months | 11.5 months | 0.00 | 0.00 | 15.36 | 0.00 | 0.00 | 0.00 | 1.59 |
| R105 | FEMALE | WILD TYPE 11 months | 11.5 months | 0.00 | 0.80 | 0.00 | 0.00 | 0.37 | 0.25 | 1.00 |
| R106 | FEMALE | WILD TYPE 11 months | 11.5 months | 0.61 | 1.45 | 0.83 | 0.00 | 0.30 | 0.34 | 0.69 |
| R319 | MALE | WILD TYPE 11 months | 10.9 months | 0.81 | 0.90 | 4.49 | 0.00 | 2.44 | 0.00 | 0.00 |
| R320 | MALE | WILD TYPE 11 months | 10.9 months | 0.30 | 0.70 | 0.43 | 0.00 | 0.00 | 0.00 | 0.00 |
| R323 | MALE | WILD TYPE 11 months | 10.9 months | 0.38 | 0.00 | 2.61 | 104.82 | 0.00 | 0.00 | 3.66 |
| R328 | FEMALE | WILD TYPE 11 months | 10.9 months | 0.19 | 0.00 | 0.00 | 0.00 | 0.00 | 0.00 | 0.00 |
| WT (POS. 1) | FEMALE | WILD TYPE 11 months | 11 months | 0.00 | 0.70 | 0.51 | 1.01 | 0.90 | 0.50 | 2.62 |
| WT (POS. 8) | FEMALE | WILD TYPE 11 months | 12 months | 0.00 | 0.00 | 0.39 | 0.00 | 0.29 | 1.79 | 5.33 |
| WT (POS. 9) | FEMALE | WILD TYPE 11 months | 11 months | 0.00 | 0.00 | 12.40 | 1.79 | 2.01 | 0.29 | 3.44 |
| WT (POS. 10) | FEMALE | WILD TYPE 11 months | 11 months | 0.00 | 0.56 | 4.45 | 1.45 | 0.01 | 0.36 | 4.37 |
| WT-19 | FEMALE | WILD TYPE 11 months | 11 months | 0.00 | 0.26 | 3.59 | 0.30 | 1.50 | 0.00 | 4.24 |
| WT-21 | FEMALE | WILD TYPE 11 months | 11 months | 0.00 | 0.00 | 1.22 | 0.35 | 0.58 | 0.00 | 3.25 |
| WT-22 | FEMALE | WILD TYPE 11 months | 11 months | 0.00 | 0.00 | 0.00 | 0.22 | 0.69 | 0.60 | 3.54 |
| R022 | MALE | WILD TYPE 18 months | 18.1 months | 0.65 | 1.20 | 10.65 | 0.00 | 0.00 | 0.00 | 0.00 |
| R104 | FEMALE | WILD TYPE 18 months | 17.9 months | 0.69 | 4.01 | 88.47 | 23.50 | 5.41 | 2.93 | 6.79 |
| R105 | FEMALE | WILD TYPE 18 months | 17.9 months | 0.15 | 1.30 | 17.90 | 0.00 | 4.06 | 0.90 | 3.66 |
| R106 | FEMALE | WILD TYPE 18 months | 17.9 months | 14.46 | 2.89 | 16.48 | 22.31 | 13.20 | 308.05 | 1.49 |
| R319 | MALE | WILD TYPE 18 months | 18.5 months | 0.00 | 0.00 | 58.00 | 0.00 | 5.64 | 0.16 | 3.42 |
| R320 | MALE | WILD TYPE 18 months | 18.5 months | 0.65 | 1.84 | 56.66 | 0.00 | 4.87 | 0.00 | 2.85 |
| R323 | MALE | WILD TYPE 18 months | 17.9 months | 0.42 | 1.50 | 22.19 | 0.00 | 0.22 | 0.12 | 0.00 |
| R328 | FEMALE | WILD TYPE 18 months | 17.8 months | 0.89 | 0.70 | 133.04 | 0.00 | 0.00 | 0.00 | 5.40 |
| WT-19 | FEMALE | WILD TYPE 18 months | 17 months | 0.00 | 0.87 | 39.11 | 5.52 | 6.32 | 1.88 | 40.72 |
| WT-21 | FEMALE | WILD TYPE 18 months | 17 months | 1.47 | 0.00 | 11.74 | 1.73 | 2.06 | 0.00 | 3.91 |
| WT-22 | FEMALE | WILD TYPE 18 months | 17 months | 0.00 | 0.62 | 3.51 | 4.41 | 5.26 | 1.88 | 5.58 |
| S749 | MALE | PAX5+/- Non-Leukemic | 14.3 months | 0.71 | 0.00 | 3.05 | 0.00 | 1.24 | 0.06 | 2.87 |
| W560 | FEMALE | PAX5+/- Non-Leukemic | 10.8 months | 0.61 | 0.00 | 0.95 | 0.00 | 0.00 | 0.38 | 1.56 |
| C351 | MALE | PAX5+/- Non-Leukemic | 7.2 months | 0.54 | 0.00 | 0.00 | 0.00 | 0.30 | 0.06 | 4.78 |
| C352 | MALE | PAX5+/- Non-Leukemic | 7.2 months | 0.46 | 0.00 | 2.73 | 0.00 | 3.09 | 0.06 | 4.94 |
| C353 | MALE | PAX5+/- Non-Leukemic | 7.2 months | 0.23 | 0.00 | 0.73 | 0.00 | 0.00 | 0.00 | 2.87 |
| B475 | FEMALE | PAX5+/- Non-Leukemic | 3 months | 0.71 | 0.00 | 1.38 | 0.00 | 0.00 | 0.00 | 2.34 |
| B472 | FEMALE | PAX5+/- Non-Leukemic | 3 months | 0.00 | 0.00 | 1.67 | 0.00 | 0.00 | 0.00 | 1.94 |
| Z188 | MALE | PAX5+/- Non-Leukemic | 3 months | 0.39 | 0.00 | 2.26 | 0.00 | 0.78 | 0.48 | 4.01 |
| Z185 | MALE | PAX5+/- Non-Leukemic | 8.5 months | 0.00 | 0.00 | 0.88 | 0.00 | 0.00 | 0.00 | 3.29 |
| Z801 | MALE | PAX5+/- Non-Leukemic | 8.5 months | 0.79 | 0.00 | 1.96 | 0.00 | 0.17 | 0.83 | 1.69 |
| O923 | FEMALE | PAX5+/- Non-Leukemic | 9.8 months | 0.75 | 0.00 | 1.82 | 0.00 | 0.00 | 1.48 | 1.56 |
| G221 | FEMALE | PAX5+/- Non-Leukemic | 2-4 months | 0.68 | 0.00 | 0.00 | 0.00 | 0.00 | 0.58 | 1.94 |
| G222 | FEMALE | PAX5+/- Non-Leukemic | 2-4 months | 0.68 | 0.00 | 3.56 | 0.00 | 0.02 | 0.00 | 2.20 |
| G239 | FEMALE | PAX5+/- Non-Leukemic | 2-4 months | 0.93 | 0.68 | 2.49 | 0.19 | 0.00 | 0.32 | 2.47 |
| G266 | FEMALE | PAX5+/- Non-Leukemic | 2-4 months | 0.75 | 0.00 | 0.88 | 0.00 | 0.00 | 0.00 | 0.64 |
| C988 | MALE | PAX5+/- Non-Leukemic | 2-4 months | 0.19 | 0.00 | 3.82 | 0.00 | 2.40 | 0.22 | 1.69 |
| C989 | MALE | PAX5+/- Non-Leukemic | 2-4 months | 0.14 | 0.00 | 1.89 | 0.00 | 0.30 | 0.00 | 1.43 |
| G221 | FEMALE | PAX5+/- Non-Leukemic | 4-5 months | 0.46 | 0.00 | 0.00 | 0.00 | 0.00 | 0.00 | 0.78 |
| G222 | FEMALE | PAX5+/- Non-Leukemic | 4-5 months | 0.57 | 0.00 | 2.65 | 0.00 | 0.10 | 0.12 | 2.07 |
| G239 | FEMALE | PAX5+/- Non-Leukemic | 4-5 months | 0.00 | 0.00 | 0.00 | 0.00 | 0.17 | 0.02 | 2.20 |
| G266 | FEMALE | PAX5+/- Non-Leukemic | 4-5 months | 0.00 | 0.00 | 0.00 | 0.00 | 0.66 | 0.00 | 0.78 |
| C988 | MALE | PAX5+/- Non-Leukemic | 4-5 months | 0.00 | 0.00 | 0.00 | 0.00 | 0.00 | 0.02 | 2.20 |
| C989 | MALE | PAX5+/- Non-Leukemic | 4-5 months | 0.00 | 0.00 | 2.19 | 0.00 | 0.00 | 0.00 | 9.18 |
| R017 | FEMALE | PAX5+/- Non-Leukemic | 5.6 months | 0.50 | 0.80 | 1.73 | 0.00 | 2.22 | 1.08 | 4.97 |
| R018 | FEMALE | PAX5+/- Non-Leukemic | 5.6 months | 0.00 | 0.00 | 0.00 | 0.00 | 0.00 | 0.01 | 0.00 |
| R019 | MALE | PAX5+/- Non-Leukemic | 5.6 months | 0.07 | 0.00 | 1.39 | 0.00 | 0.00 | 0.00 | 3.91 |
| R020 | MALE | PAX5+/- Non-Leukemic | 5.6 months | 0.00 | 0.00 | 0.00 | 0.00 | 0.00 | 0.00 | 0.00 |
| R021 | FEMALE | PAX5+/- Non-Leukemic | 5.6 months | 0.00 | 0.00 | 0.00 | 0.00 | 1.01 | 0.41 | 0.00 |
| R103 | MALE | PAX5+/- Non-Leukemic | 5.3 months | 0.00 | 0.00 | 91.49 | 0.00 | 25.66 | 0.68 | 1.19 |
| R322 | MALE | PAX5+/- Non-Leukemic | 5.1 months | 0.00 | 0.00 | 1.08 | 0.00 | 0.82 | 0.00 | 1.29 |
| R324 | FEMALE | PAX5+/- Non-Leukemic | 5.1 months | 0.00 | 0.00 | 0.00 | 0.00 | 3.16 | 1.08 | 0.00 |
| R327 | FEMALE | PAX5+/- Non-Leukemic | 5.1 months | 0.00 | 0.00 | 0.00 | 0.00 | 0.00 | 0.00 | 0.00 |
| R329 | FEMALE | PAX5+/- Non-Leukemic | 5.1 months | 0.00 | 0.00 | 0.98 | 0.00 | 0.00 | 0.00 | 1.89 |
| R330 | FEMALE | PAX5+/- Non-Leukemic | 5.1 months | 0.00 | 0.00 | 0.00 | 0.00 | 0.00 | 0.00 | 1.19 |
| R331 | FEMALE | PAX5+/- Non-Leukemic | 5.1 months | 0.57 | 0.00 | 0.38 | 0.00 | 0.95 | 0.53 | 3.30 |
| R017 | FEMALE | PAX5+/- Non-Leukemic | 11.8 months | 0.00 | 1.50 | 2.41 | 0.00 | 1.26 | 1.21 | 2.52 |
| R018 | FEMALE | PAX5+/- Non-Leukemic | 11.8 months | 0.00 | 1.25 | 0.00 | 0.00 | 0.00 | 0.74 | 0.00 |
| R019 | MALE | PAX5+/- Non-Leukemic | 11.8 months | 0.00 | 0.00 | 146.66 | 0.00 | 17.65 | 0.12 | 11.64 |
| R020 | MALE | PAX5+/- Non-Leukemic | 11.8 months | 0.00 | 0.00 | 1.08 | 0.00 | 0.00 | 0.00 | 0.00 |
| R021 | FEMALE | PAX5+/- Non-Leukemic | 11.8 months | 0.00 | 0.00 | 0.33 | 0.00 | 1.07 | 0.29 | 0.00 |
| R103 | MALE | PAX5+/- Non-Leukemic | 11.5 months | 0.00 | 0.00 | 3.17 | 0.00 | 0.00 | 0.03 | 7.81 |
| R322 | MALE | PAX5+/- Non-Leukemic | 10.9 months | 0.15 | 1.05 | 3.03 | 0.00 | 27.12 | 0.00 | 0.00 |
| R327 | FEMALE | PAX5+/- Non-Leukemic | 10.9 months | 0.26 | 0.26 | 0.93 | 0.00 | 0.00 | 0.25 | 3.42 |
| R329 | FEMALE | PAX5+/- Non-Leukemic | 10.9 months | 0.00 | 0.00 | 0.00 | 0.00 | 0.00 | 0.00 | 0.00 |
| R330 | FEMALE | PAX5+/- Non-Leukemic | 10.9 months | 0.00 | 0.00 | 0.16 | 0.00 | 0.00 | 0.00 | 5.69 |
| R331 | FEMALE | PAX5+/- Non-Leukemic | 10.9 months | 0.00 | 0.00 | 0.00 | 0.00 | 0.00 | 0.00 | 2.52 |
| R018 | FEMALE | PAX5+/- Non-Leukemic | 18.1 months | 0.38 | 1.84 | 3.32 | 0.00 | 1.14 | 0.63 | 0.00 |
| R019 | MALE | PAX5+/- Non-Leukemic | 18.1 months | 1.01 | 1.05 | 197.84 | 0.00 | 6.48 | 1.08 | 13.55 |
| R020 | MALE | PAX5+/- Non-Leukemic | 18.1 months | 0.61 | 0.34 | 4.86 | 0.00 | 0.00 | 0.00 | 1.00 |
| R021 | FEMALE | PAX5+/- Non-Leukemic | 18.1 months | 0.26 | 2.51 | 3.25 | 0.00 | 3.25 | 1.95 | 0.59 |
| R103 | MALE | PAX5+/- Non-Leukemic | 17.9 months | 1.86 | 1.84 | 7.88 | 0.00 | 2.60 | 1.30 | 0.00 |
| R322 | MALE | PAX5+/- Non-Leukemic | 17.8 months | 5.12 | 4.01 | 124.63 | 0.00 | 2.67 | 1.05 | 4.70 |
| R327 | FEMALE | PAX5+/- Non-Leukemic | 17.8 months | 0.50 | 1.40 | 3.17 | 0.00 | 0.00 | 0.10 | 1.89 |
| R329 | FEMALE | PAX5+/- Non-Leukemic | 17.8 months | 0.00 | 3.23 | 58.00 | 0.00 | 2.07 | 0.55 | 2.41 |
| R331 | FEMALE | PAX5+/- Non-Leukemic | 17.8 months | 0.69 | 8.55 | 108.07 | 58.26 | 9.34 | 3.97 | 9.11 |
| R021 | FEMALE | PAX5+/- Non-Leukemic | 5 months | 0.00 | 0.00 | 0.00 | 0.12 | 0.35 | 0.00 | 4.87 |
| R016 | MALE | PAX5+/- Non-Leukemic | 5 months | 0.00 | 0.43 | 0.78 | 2.60 | 0.76 | 0.33 | 5.33 |
| R020 | MALE | PAX5+/- Non-Leukemic | 5 months | 0.00 | 0.00 | 0.40 | 0.56 | 0.24 | 0.00 | 3.20 |
| R021 | FEMALE | PAX5+/- Non-Leukemic | 11 months | 0.00 | 0.00 | 0.82 | 3.32 | 1.25 | 0.00 | 5.26 |
| R016 | MALE | PAX5+/- Non-Leukemic | 11 months | 0.00 | 1.58 | 4.71 | 13.50 | 4.62 | 0.97 | 11.47 |
| R020 | MALE | PAX5+/- Non-Leukemic | 11 months | 0.00 | 0.68 | 28.23 | 5.94 | 2.78 | 0.00 | 10.49 |
| R021 | FEMALE | PAX5+/- Non-Leukemic | 17 months | 0.00 | 0.00 | 1.01 | 0.05 | 0.38 | 0.97 | 2.25 |
| R016 | MALE | PAX5+/- Non-Leukemic | 17 months | 0.00 | 4.31 | 67.82 | 40.81 | 11.65 | 7.51 | 13.67 |
| R020 | MALE | PAX5+/- Non-Leukemic | 17 months | 0.00 | 0.00 | 3.51 | 1.68 | 0.41 | 0.00 | 3.66 |
| S748 | MALE | PAX5+/- Leukemic | 14.2 months | 1.12 | 5.75 | 1962.29 | 2.43 | 10.88 | 0.97 | 13.66 |
| O332 | FEMALE | PAX5+/- Leukemic | 15.3 months | 0.00 | 5.31 | 7.78 | 9.96 | 6.88 | 3.29 | 3.29 |
| S556 | MALE | PAX5+/- Leukemic | 6.5 months | 1.02 | 0.80 | 6.45 | 1.41 | 0.00 | 0.12 | 26.91 |
| O388 | MALE | PAX5+/- Leukemic | 9.5 months | 0.79 | 0.00 | 6.45 | 0.00 | 0.00 | 0.00 | 35.99 |
| O361 | FEMALE | PAX5+/- Leukemic | 8.8 months | 1.00 | 1.12 | 4.66 | 0.00 | 0.02 | 0.00 | 163.18 |
| S767 | MALE | PAX5+/- Leukemic | 15.3 months | 0.89 | 0.00 | 40.38 | 0.00 | 1.12 | 0.00 | 6.46 |
| W893 | MALE | PAX5+/- Leukemic | 9.8 months | 0.82 | 1.02 | 4.18 | 5.87 | 0.78 | 1.40 | 100.53 |
| R324 | FEMALE | PAX5+/- Leukemic | 10.4 months | 0.34 | 0.00 | 40.63 | 0.00 | 0.00 | 0.14 | 4.17 |
| R330 | FEMALE | PAX5+/- Leukemic | 12 months | 0.11 | 0.00 | 693.53 | 0.00 | 0.00 | 0.00 | 96.50 |
| T830 | MALE | PAX5+/- Leukemic | 9 months | 0.00 | 0.00 | 590.44 | 0.00 | 0.00 | 0.00 | 71.47 |
| R017 | FEMALE | PAX5+/- Leukemic | 17.3 months | 0.00 | 0.00 | 61.47 | 0.00 | 0.00 | 0.00 | 164.44 |
| R330 | FEMALE | PAX5+/- Leukemic | 12 months | 0.00 | 0.00 | 869.54 | 0.00 | 0.00 | 0.00 | 169.93 |
| J696 | FEMALE | BCR-ABLp190 | 16.6 months | 0.00 | 0.00 | 3.53 | 0.00 | 0.00 | 0.18 | 2.87 |
| M510 | MALE | BCR-ABLp190 | 14.2 months | 0.00 | 0.00 | 35.1 | 0.00 | 0.00 | 0.00 | 0.00 |
| M593 | FEMALE | BCR-ABLp190 | 14 months | 0.00 | 0.00 | 0.00 | 226.9 | 33.8 | 1.58 | 0.00 |
| P990 | MALE | BCR-ABLp190 | 18.5 months | 0.00 | 0.00 | 8.5 | 1.07 | 0.95 | 0.38 | 2.05 |
| W000 | MALE | BCR-ABLp190 | 6.8 months | 0.00 | 0.00 | 27.40 | 0.00 | 0.00 | 0.00 | 1.24 |
| C127 | MALE | BCR/ABLp190+PAX5+/- | 8.9 months | 0.25 | 0.00 | 2528.40 | 3.16 | 3.44 | 25.22 | 191.76 |
| C219 | MALE | BCR/ABLp190+PAX5+/- | 8.4 months | 0.00 | 3.37 | 1182.86 | 4.98 | 30.52 | 4.36 | 40.16 |
| C930 | FEMALE | BCR/ABLp190+PAX5+/- | 8.3 months | 0.00 | 0.00 | 0.00 | 0.00 | 0.00 | 0.00 | 8.65 |
| C932 | FEMALE | BCR/ABLp190+PAX5+/- | 11.7 months | 0.28 | 2.52 | 119.86 | 1.32 | 4.27 | 0.30 | 6.64 |
| O171 | FEMALE | BCR/ABLp190+PAX5+/- | 9 months | 0.00 | 0.00 | 63.39 | 17.77 | 0.00 | 4.93 | 135.62 |
| O172 | FEMALE | BCR/ABLp190+PAX5+/- | 9.6 months | 0.00 | 0.00 | 0.00 | 0.00 | 0.00 | 0.00 | 8.13 |
| O952 | MALE | BCR/ABLp190+PAX5+/- | 11.1 months | 0.00 | 0.00 | 8.67 | 1.07 | 1.85 | 0.01 | 0.82 |
| Q842 | MALE | BCR/ABLp190+PAX5+/- | 12.4 months | 0.00 | 0.00 | 2.97 | 0.00 | 0.00 | 0.00 | 70.10 |
| Q844 | FEMALE | BCR/ABLp190+PAX5+/- | 12.3 months | 0.00 | 0.00 | 3.19 | 0.00 | 0.00 | 0.00 | 2.66 |
| S314 | FEMALE | BCR/ABLp190+PAX5+/- | 8.8 months | 0.00 | 0.00 | 3.76 | 0.00 | 0.00 | 0.00 | 7.13 |
| S650 | MALE | BCR/ABLp190+PAX5+/- | 7 months | 0.00 | 0.00 | 414.42 | 0.00 | 257.22 | 4.48 | 14.11 |
| W555 | MALE | BCR/ABLp190+PAX5+/- | 6.2 months | 0.00 | 0.30 | 42.44 | 0.00 | 0.00 | 0.08 | 55.76 |
| W556 | MALE | BCR/ABLp190+PAX5+/- | 8.5 months | 0.00 | 0.00 | 27.74 | 0.00 | 12.74 | 0.00 | 13.79 |
| W558 | FEMALE | BCR/ABLp190+PAX5+/- | 9.5 months |  |  | 145.7 |  |  |  |  |
| W870 | MALE | BCR/ABLp190+PAX5+/- | 12.3 months |  |  | 188.7 |  |  |  |  |
| W871 | MALE | BCR/ABLp190+PAX5+/- | 7.2 months |  |  | 1367.6 |  |  |  |  |
| X402 | FEMALE | BCR/ABLp190+PAX5+/- | 9.7 months |  |  | 2.3 |  |  |  |  |
| X403 | FEMALE | BCR/ABLp190+PAX5+/- | 9.7 months |  |  | 0 |  |  |  |  |
| X669 | FEMALE | BCR/ABLp190+PAX5+/- | 8.7 months |  |  | 354.9 |  |  |  |  |
| Z595 | MALE | BCR/ABLp190+PAX5+/- | 10.10 months |  |  | 1172.2 |  |  |  |  |
| J406 | FEMALE | ETV6-RUNX1 | 18.3 months |  |  | 0.29 |  |  |  |  |
| J408 | FEMALE | ETV6-RUNX1 | 18.3 months |  |  | 2.89 |  |  |  |  |
| K210 | MALE | ETV6-RUNX1 | 20.5 months |  |  | 27.3 |  |  |  |  |
| K213 | MALE | ETV6-RUNX1 | 17.6 months |  |  | 30.22 |  |  |  |  |
| R899 | MALE | ETV6-RUNX1 | 14.4 months |  |  | 44.2 |  |  |  |  |
| S825 | FEMALE | ETV6-RUNX1 | 15.4 months |  |  | 2.04 |  |  |  |  |
| G549 | FEMALE | ETV6-RUNX1 PAX5+/- | 14.80 months |  |  | 590.6 |  |  |  |  |
| K373 | FEMALE | ETV6-RUNX1 PAX5+/- | 11.27 months |  |  | 2958 |  |  |  |  |
| K488 | MALE | ETV6-RUNX1 PAX5+/- | 11.53 months |  |  | 360.5 |  |  |  |  |
| K772 | FEMALE | ETV6-RUNX1 PAX5+/- | 14.57 months |  |  | 40.1 |  |  |  |  |
| S238 | FEMALE | ETV6-RUNX1 PAX5+/- | 17.8 months |  |  | 180.3 |  |  |  |  |
| S372 | FEMALE | ETV6-RUNX1 PAX5+/- | 9.6 months |  |  | 8.8 |  |  |  |  |
| U572 | MALE | IL6+/- + PAX5+/- Non-Leukemic | 5 months |  |  | 0.56 |  |  |  |  |
| L370 | MALE | IL6+/- + PAX5+/- Non-Leukemic | 5 months |  |  | 0.67 |  |  |  |  |
| U580 | FEMALE | IL6+/- + PAX5+/- Non-Leukemic | 5 months |  |  | 0 |  |  |  |  |
| U572 | MALE | IL6+/- + PAX5+/- Non-Leukemic | 11 months |  |  | 0.85 |  |  |  |  |
| L370 | MALE | IL6+/- + PAX5+/- Non-Leukemic | 11 months |  |  | 0.31 |  |  |  |  |
| U580 | FEMALE | IL6+/- + PAX5+/- Non-Leukemic | 11 months |  |  | 0 |  |  |  |  |
| U572 | MALE | IL6+/- + PAX5+/- Non-Leukemic | 17 months |  |  | 0.6 |  |  |  |  |
| L370 | MALE | IL6+/- + PAX5+/- Non-Leukemic | 17 months |  |  | 0.31 |  |  |  |  |
| U580 | FEMALE | IL6+/- + PAX5+/- Non-Leukemic | 17 months |  |  | 0.53 |  |  |  |  |
| U971 | FEMALE | IL6+/- + PAX5+/- Non-Leukemic | 5 months |  |  | 0 |  |  |  |  |
| U971 | FEMALE | IL6+/- + PAX5+/- Non-Leukemic | 11 months |  |  | 0 |  |  |  |  |
| L371 | MALE | IL6+/- + PAX5+/- Non-Leukemic | 5 months |  |  | 0 |  |  |  |  |
| L371 | MALE | IL6+/- + PAX5+/- Non-Leukemic | 11 months |  |  | 0 |  |  |  |  |
| L371 | MALE | IL6+/- + PAX5+/- Non-Leukemic | 17 months |  |  | 0 |  |  |  |  |
| U971 | FEMALE | IL6+/- + PAX5+/- Leukemic | 18 months |  |  | 39.11 |  |  |  |  |
| U572 | FEMALE | IL6+/- + PAX5+/- Leukemic | 21 months |  |  | 6.25 |  |  |  |  |
| L370 | MALE | IL6+/- + PAX5+/- Leukemic | 20 months |  |  | 297.45 |  |  |  |  |
