# Supplementary material for: Inhibition of inflammatory signaling in Pax5 mutant cells mitigates B-cell leukemogenesis
Source: Sci Rep. 2020 Nov 5;10:19189. doi: 10.1038/s41598-020-76206-y (PMC7644722; doi:10.1038/s41598-020-76206-y)
Supplement: Supplementary file 3 — Supplementary Information [file 41598_2020_76206_MOESM3_ESM.docx]

**Supplementary Table S2.** **Human serum samples description.** Demographics, genotype and IL6 serum levels of PAX5^+/-^ (n=6) and healthy

controls (n=20).

| CODE | GENDER | GENOTYPE | Age at time of determination | IL-6 levels (pg/ml) | White blood cells (x 10^9^/L) |
| --- | --- | --- | --- | --- | --- |
| 682 | FEMALE | CONTROL | 49 years | 0 |  |
| 88 | MALE | CONTROL | 53 years | 0 |  |
| 904 | MALE | CONTROL | 57 years | 0 |  |
| 368 | MALE | CONTROL | 59 years | 0 |  |
| 441 | FEMALE | CONTROL | 45 years | 0 |  |
| 675 | MALE | CONTROL | 46 years | 0 |  |
| 542 | FEMALE | CONTROL | 54 years | 0 |  |
| 629 | FEMALE | CONTROL | 56 years | 0 |  |
| 862 | FEMALE | CONTROL | 84 years | 1 |  |
| 666 | MALE | CONTROL | 79 years | 1.1 |  |
| 257 | FEMALE | CONTROL | 78 years | 1 |  |
| 893 | FEMALE | CONTROL | 70 years | 0 |  |
| 927 | FEMALE | CONTROL | 67 years | 0 |  |
| 908 | FEMALE | CONTROL | 63 years | 0 |  |
| 513 | MALE | CONTROL | 59 years | 0.3 |  |
| 941 | MALE | CONTROL | 58 years | 0 |  |
| 292 | FEMALE | CONTROL | 37 years | 0 |  |
| 922 | FEMALE | CONTROL | 39 years | 0 |  |
| 993 | FEMALE | CONTROL | 40 years | 0 |  |
| 705 | MALE | CONTROL | 41 years | 0.3 |  |
| 945/10 SP | FEMALE | PAX5+/- | 21 years | 0.3 | 58,3 |
| 1274/11 SP | FEMALE | PAX5+/- | 68 years | 107.2 | 8,5 |
| 1315/12 SP | FEMALE | PAX5+/- | 38 years | 0.9 | 45 |
| AAA0442817 | FEMALE | PAX5+/- | 27 years | 23.6 | 2,2 |
| AAA4021844 | FEMALE | PAX5+/- | 22 years | 26.3 | 190 |
| AAA4021889 | MALE | PAX5+/- | 39 years | 1.9 | 23 |
