# Supplementary material for: Inhibition of inflammatory signaling in Pax5 mutant cells mitigates B-cell leukemogenesis
Source: Sci Rep. 2020 Nov 5;10:19189. doi: 10.1038/s41598-020-76206-y (PMC7644722; doi:10.1038/s41598-020-76206-y)
Supplement: Supplementary file 6 — Supplementary Information [file 41598_2020_76206_MOESM6_ESM.docx]

**Supplementary Table S5.** **Samples description and IL6 serum levels of Pax5^+/-^ Leukemic mice, mice transplanted with leukemic Pax5 proB cells and *in vitro* samples supernatant from proB cell medium culture to test IL6 levels.** Already known IL6 levels in proB cell medium culture (50pg/mL, 500pg/mL, 5ng/mL and 50ng/mL) were used as positive controls of *in vitro* IL6 determination. NA: not applicable.

| CODE | GENDER | GENOTYPE | Age at time of determination (months) | IL-6 levels (pg/ml) |
| --- | --- | --- | --- | --- |
| R017 | MALE | PAX5+/- Leukemic | 17,27 | 275,3086 |
| L220 | FEMALE | PAX5+/- Leukemic | 8,76 | 167,8220 |
| L643 | FEMALE | PAX5+/- Leukemic | 8,3 | 815,7584 |
| WT-1 | FEMALE | WT transplanted with leukemic Pax5+/- proB cells | 7,1 | 7,0941 |
| WT-2 | FEMALE | WT transplanted with leukemic Pax5+/- proB cells | 6,33 | 13,2895 |
| WT-3 | FEMALE | WT transplanted with leukemic Pax5+/- proB cells | 6,33 | 3,7608 |
| WT-4 | FEMALE | WT transplanted with leukemic Pax5+/- proB cells | 6,33 | 4,3009 |
| WT-5 | FEMALE | WT transplanted with leukemic Pax5+/- proB cells | 5,6 | 0,2168 |
| WT-6 | FEMALE | WT transplanted with leukemic Pax5+/- proB cells | 5,6 | 0,8745 |
| WT-7 | FEMALE | WT transplanted with leukemic Pax5+/- proB cells | 5,6 | 11,5685 |
| WT-8 | FEMALE | WT transplanted with leukemic Pax5+/- proB cells | 5,6 | 15,6126 |
| WT-9 | FEMALE | WT transplanted with leukemic Pax5+/- proB cells | 5,6 | 42,8517 |
| WT-10 | FEMALE | WT transplanted with leukemic Pax5+/- proB cells | 5,6 | 9,2859 |
| WT-1 | FEMALE | WT transplanted with leukemic Pax5+/- proB cells | 7,1 | 114,6818 |
| WT-2 | FEMALE | WT transplanted with leukemic Pax5+/- proB cells | 6,33 | 0,0000 |
| WT-3 | FEMALE | WT transplanted with leukemic Pax5+/- proB cells | 6,33 | 0,0000 |
| WT-4 | FEMALE | WT transplanted with leukemic Pax5+/- proB cells | 6,33 | 0,0000 |
| WT-5 | FEMALE | WT transplanted with leukemic Pax5+/- proB cells | 5,6 | 4,3259 |
| WT-6 | FEMALE | WT transplanted with leukemic Pax5+/- proB cells | 5,6 | 6,6569 |
| WT-7 | FEMALE | WT transplanted with leukemic Pax5+/- proB cells | 5,6 | 25,5286 |
| WT-8 | FEMALE | WT transplanted with leukemic Pax5+/- proB cells | 5,6 | 0,0000 |
| WT-9 | FEMALE | WT transplanted with leukemic Pax5+/- proB cells | 5,6 | 0,2184 |
| WT-10 | FEMALE | WT transplanted with leukemic Pax5+/- proB cells | 5,6 | 6,2587 |
| 24 | NA | proB cell medium culture + IL6 (50pg/mL) | NA | 3,7629 |
| 25 | NA | proB cell medium culture + IL6 (50pg/mL) | NA | 4,6881 |
| 26 | NA | proB cell medium culture + IL6 (500pg/mL) | NA | 57,5885 |
| 27 | NA | proB cell medium culture + IL6 (500pg/mL) | NA | 54,6452 |
| 28 | NA | proB cell medium culture + IL6 (5ng/mL) | NA | 798,1938 |
| 29 | NA | proB cell medium culture + IL6 (5ng/mL) | NA | 767,0316 |
| 30 | NA | proB cell medium culture + IL6 (50ng/mL) | NA | 5432,4566 |
| 31 | NA | proB cell medium culture + IL6 (50ng/mL) | NA | 4290,6519 |
| 4A-1 | NA | proB cell medium supernatant from Pax5+/- proB cells | NA | 1,5725 |
| 4A-2 | NA | proB cell medium supernatant from Pax5+/- proB cells | NA | 1,5355 |
| 4A-3 | NA | proB cell medium supernatant from Pax5+/- proB cells | NA | 1,2906 |
| S748-1 | NA | proB cell medium supernatant from Pax5+/- proB cells | NA | 0,2514 |
| S748-2 | NA | proB cell medium supernatant from Pax5+/- proB cells | NA | 0,2697 |
| S748-3 | NA | proB cell medium supernatant from Pax5+/- proB cells | NA | 0,5633 |
